# Supplementary material for: Effects of presurgical interventions on chronic pain after total knee replacement: a systematic review and meta-analysis of randomised controlled trials
Source: BMJ Open. 2020 Jan 20;10(1):e033248. doi: 10.1136/bmjopen-2019-033248 (PMC7045074; doi:10.1136/bmjopen-2019-033248)
Supplement: Supplementary data [file bmjopen-2019-033248supp001.pdf]

Appendix 1. Search strategy as applied in MEDLINE (tailored as relevant to other databases)

1 randomized controlled trial/ or randomized controlled trial.pt.

2 controlled clinical trial.pt.

3 randomized.ab.

4 placebo.ab.

5 randomly.ab

6 trial.ab

7 randomised.tw

8 1 or 2 or 3 or 4 or 5 or 6 or 7

9 review/

10 'systematic review$'.mp

11 9 or 10

12 8 or 11

13 Arthroplasty, Replacement, Knee/

14 Knee Prosthesis/

15 (arthoplast$ adj3 knee$).mp. [mp=title, abstract, original title, name of substance word, subject heading word, keyword heading word, protocol supplementary concept word, rare disease supplementary concept word, unique identifier]

16 (knee$ adj3 replac$).mp. [mp=title, abstract, original title, name of substance word, subject heading word, keyword heading word, protocol supplementary concept word, rare disease supplementary concept word, unique identifier]

17 (knee adj3 implant$).mp. [mp=title, abstract, original title, name of substance word, subject heading word, keyword heading word, protocol supplementary concept word, rare disease supplementary concept word, unique identifier]

18 13 or 14 or 15 or 16 or 17

19 12 and 18
