# Supplementary material for: Effects of presurgical interventions on chronic pain after total knee replacement: a systematic review and meta-analysis of randomised controlled trials
Source: BMJ Open. 2020 Jan 20;10(1):e033248. doi: 10.1136/bmjopen-2019-033248 (PMC7045074; doi:10.1136/bmjopen-2019-033248)
Supplement: Author's manuscript [file bmjopen-2019-033248.draft_revisions.pdf]

BMJ Open

BMJ Open is committed to open peer review. As part of this commitment we make the peer review history of every article we publish publicly available.

When an article is published we post the peer reviewers' comments and the authors' responses online. We also post the versions of the paper that were used during peer review. These are the versions that the peer review comments apply to.

The versions of the paper that follow are the versions that were submitted during the peer review process. They are not the versions of record or the final published versions. They should not be cited or distributed as the published version of this manuscript.

BMJ Open is an open access journal and the full, final, typeset and author-corrected version of record of the manuscript is available on our site with no access controls, subscription charges or pay-per-view fees (http://bmjopen.bmj.com).

If you have any questions on BMJ Open's open peer review process please email info.bmjopen@bmj.com

BMJ Open

The effects of pre-surgical interventions on chronic pain after total knee replacement: A systematic review and meta-analysis of randomised controlled trials

Journal:	*BMJ Open*
Manuscript ID	bmjopen-2019-033248
Article Type:	Research
Date Submitted by the Author:	30-Jul-2019

Complete List of Authors:	Dennis, Jane; University of Bristol, Musculoskeletal Research Unit<br>Wylde, Vikki; University of Bristol, Musculoskeletal Research Unit<br>Gooberman-Hill, Rachael; University of Bristol, Bristol Medical School<br>Blom, AW; University of Bristol,<br>Beswick, Andrew; University of Bristol, Musculoskeletal Research Unit

Keywords:	chronic post-surgical pain, prevention, systematic review, total knee replacement

SCHOLARONE™
Manuscripts

The effects of pre-surgical interventions on chronic pain after total knee replacement: A systematic review and meta-analysis of randomised controlled trials

Jane Dennis[1]

Vikki Wylde[1,2]

Rachael Gooberman-Hill[1,2]

Ashley W Blom[1,2]

Andrew D Beswick[1]

[1] Musculoskeletal Research Unit, Translational Health Sciences, Bristol Medical School, University of Bristol

[2] National Institute for Health Research Bristol Biomedical Research Centre, University Hospitals Bristol NHS Foundation Trust and University of Bristol

jane.dennis@bristol.ac.uk,v.wylde@bristol.ac.uk,r.gooberman-hill@bristol.ac.uk,ashley.blom@bristol.ac.uk,andy.beswick@bristol.ac.uk

Corresponding author: Jane Dennis

Word count: 4497

Musculoskeletal Research Unit
Translational Health Sciences
Bristol Medical School
University of Bristol
Learning and Research Building, Level 1
Southmead Hospital
Bristol
BS10 5NB

Keywords: chronic post-surgical pain; prevention; systematic review; total knee replacement

For peer review only

For peer review only - http://bmjopen.bmj.com/site/about/guidelines.xhtml

Abstract

Objective

Nearly 100,000 primary total knee replacements [TKR] are performed in the UK annually. The primary aim of TKR is pain relief, but 10-34% of patients report chronic pain. This aim of this systematic review was to evaluate the effectiveness of pre-surgical interventions in preventing chronic pain after TKR.

Design

MEDLINE, Embase, CINAHL, *The Cochrane Library* and PsycINFO were searched from inception to December 2018. Screening and data extraction were performed by two reviewers. Meta-analysis was conducted using a random effects model. Risk of bias was assessed using the Cochrane tool and quality of evidence assessed by GRADE

Primary and secondary outcomes

Pain at six months or longer; adverse events.

Interventions

Presurgical interventions aimed at improving TKR outcomes.

Results

Eight RCTs with data from 960 participants were included. The studies involved nine eligible comparisons. We found moderate quality evidence of no effect of exercise programmes on chronic pain after TKR, based on a meta-analysis of 6 studies with 229 participants (SMD 0.20, 95% CI -0.06 to 0.47). There was no statistical heterogeneity and sensitivity analysis restricted to studies at overall low risk of bias confirmed findings. Studies evaluating exercise (n=1), combined exercise and education intervention (n=1) and education alone (n=1) suggested similar findings. Adverse event data were reported by most studies, but events were too few to draw conclusions.

Conclusions

We found low to moderate quality evidence to suggest that neither pre-operative exercise, education nor a combination of both is effective in preventing chronic pain after TKR. This review also identified a lack of evaluation of other pre-operative interventions, such as multi-modal pain management, which may improve long-term pain outcomes after TKR.

PROSPERO REGISTRATION NUMBER: CRD42017041382.

For peer review only - http://bmjopen.bmj.com/site/about/guidelines.xhtml

Strengths and limitations of this study

- This systematic review evaluates recent evidence of the effectiveness of presurgical interventions in preventing long-term chronic pain after total knee replacement
- Synthesis of adverse events data was not possible
- We did not include studies that extended beyond the knee replacement

For peer review only

For peer review only - http://bmjopen.bmj.com/site/about/guidelines.xhtml

Background

Osteoarthritis is the most common condition that affects the knee joint and causes considerable pain and disability. Effective treatments noted in guidelines include pain medications, exercise, and weight loss when appropriate[1],[2]. If symptoms do not respond to pharmacological and conservative treatments, people may receive a total knee replacement (TKR). Annually in the UK, nearly 100,000 primary TKRs are performed [3 4] and in the USA in 2010, about five million people were estimated to be living with a TKR [5].

Knee pain at rest and during walking are important indications which guide decisions for joint replacement in people with osteoarthritis [6]. Outcomes are good for many, but a systematic review found that 10-34% of patients report unfavourable long-term pain outcomes at between 3 months and 5 years after TKR [7] , which is associated with dissatisfaction with the outcome of surgery [8 9] .

Some patient factors are associated with poor long-term pain outcomes. Using structural equation modelling, Sayers and colleagues demonstrated that pain during the pre-surgical period, particularly on movement, is strongly associated with chronic post-surgical pain, and a potentially important target for intervention [10]. Other pre-surgical risk factors for pain identified in multivariable analyses in representative populations include: poorer physical function, body mass index ≥35 kg/m^2 and poor general mental health [11]. Associations between risk factors and chronic pain may be caused by inflammatory mechanisms [10], or partly explained by catastrophizing leading to heightened pain and emotional distress [12], or delays to recovery and rehabilitation.

With knowledge of risk factors for chronic pain, there is the potential for targeting care to those at high risk, or for risk factor modification [11 13]. While many pre-surgical interventions focus on preparing the patient for the procedure and hospital stay, reducing peri-operative pain and facilitating early mobilisation and recovery [11], their impact on the key outcome of long-term pain remains to be established. This is achievable through high quality, adequately powered randomised controlled trials with long-term follow up for patient related pain outcomes and ultimately through well-conducted systematic reviews with thorough consideration of sources of bias that may influence study results.

In this systematic review and meta-analysis, we assessed the effectiveness of pre-operative interventions in preventing chronic pain in patients receiving TKR. Previous reviews have largely focused on specific interventions but we have opted for a temporal framework and sought to include any intervention conducted before surgery, as part of a broad suite of reviews considering the timing of intervention throughout the TKR care trajectory [14 15].

For peer review only - http://bmjopen.bmj.com/site/about/guidelines.xhtml

Methods

The protocol for this systematic review was registered with PROSPERO (CRD42017041382) [15]. We formulated the research question according to the PICO principle [16] and used methods based on those recommended by Cochrane [17]. Reporting was in accordance with Preferred Reporting Items for Systematic Reviews and Meta-Analyses (PRISMA) guidelines [18] and a checklist is included in the Appendix.

Patient and public involvement (PPI)

Before starting the review, we held a meeting with stakeholders and patient representatives and discussed inclusion criteria and outcomes. Areas of potential relevance for pre-surgical intervention were highlighted, specifically exercise, education, psychological therapies, weight management, nutritional supplements, management of co-morbidities, and pain management with intra-articular injections. The importance of patient-reported pain as an outcome after TKR was emphasised. A musculoskeletal PPI forum has discussed the review and will advise on plain language summaries and dissemination[19].

Types of studies

To limit selection bias, we included only randomised controlled trials. Studies reported only as abstracts or unobtainable as full text copies were excluded. Language was not an exclusion criterion.

Participants

"Pre-surgical" adult patients (defined as those on waiting lists for knee replacement, up until the point of admission for surgery) who consented to trial participation.

Interventions

Preventive interventions (pharmacological or non-pharmacological) delivered during the pre-surgical period were included. Studies where delivery of the intervention extended into the postoperative period were excluded.

Comparator group

Comparator group participants included those who had received usual care or an alternative intervention.

Outcomes

In preference, we considered patient-reported joint-specific pain intensity, typically measured using pain domains of the Oxford Knee Score (OKS) [20], Western Ontario and McMaster Universities Osteoarthritis Index (WOMAC) [21] or Knee injury and Osteoarthritis Outcome

For peer review only - http://bmjopen.bmj.com/site/about/guidelines.xhtml

Score (KOOS) [22] at six months or longer after knee replacement. We selected these outcomes in preference to pain components of Knee Society's Clinical and Functional Scoring System (KSS) [23] and Hospital for Special Surgery Knee Score (HSS) [24] in which pain assessment is limited to one or two questions on intensity [25] and relies on clinician assessment which may not reflect the views of patients [26] . The choice of outcome timing reflects the time when, for many, pain levels have optimised [27]. If joint specific measures were not available, then we planned to use pain dimensions from quality of life measures including Short Form 36 Health Survey (SF-36) [28] and Short Form 12 Health Survey (SF-12) [29]. Had neither joint-specific nor generic pain outcomes been available, we planned to use data for pain measured using a visual analogue scale (VAS) or numerical rating scale (NRS). When no patient-reported outcome measure was reported, we used pain data from surgeon-assessed scores. When discrete pain data were unavailable, we report overall data that included those relating to pain, typically in combination with function and other factors.

Where data were available, we planned to report proportions of patients with a favourable pain outcome (for example "no worse than mild pain") as recommended in clinical trials [30] and systematic reviews [31].

Data on all adverse events were extracted and summarised narratively. Our focus was on adverse events related to the intervention, although we also report those related to surgery.

Study design

Randomised controlled trials of interventions commencing in the pre-surgical period, with follow up at six months or longer after surgery for total knee replacement, were identified and assessed for eligibility. Authors reporting long-term outcomes of any type (typically, function only) were contacted to check whether pain outcome data were also available; those reporting aggregated data for hip replacements together with knee replacements were also contacted. Studies reporting pain data or composite scores including pain were included.

Database searches

We established a database of all randomised controlled in knee replacement, in preparation for a suite of systematic reviews in chronic pain after TKR [14 15]. Relevant trials were identified through searches (last updated on 18th December 2018) in: MEDLINE, Embase and PsycINFO on OvidSP; CINAHL on EBSCOhost; and *The Cochrane Library*. The search strategy as applied in MEDLINE is shown in Appendix 1. Within this resource, we searched for interventions conducted in the pre-surgical setting.

Citations and reference lists of key reviews and randomised trials were checked in ISI Web of Science. The ISRTN registry on BioMed Central was searched for ongoing randomised

For peer review only - http://bmjopen.bmj.com/site/about/guidelines.xhtml

trials which were then checked for subsequent publication. No language restrictions were applied, and data from potentially relevant non-English language articles were extracted and translated to establish eligibility. Studies reported only as abstracts or that we were unable to acquire as full text copies using inter-library loans or author contact were excluded.

Study screening and data extraction

Records identified by searches were imported into Endnote X9 (Thomson Reuters). An initial screen for potential eligibility was undertaken by one review author (JD) with articles excluded only if clearly irrelevant. Typically, these were studies in osteoarthritis with no surgery, arthroscopy only, or surgery involving joints other than the knee. Subsequently, abstracts and full text articles were screened independently by two review authors. After consensus, reasons for exclusion were recorded.

Data from relevant randomised trials were extracted by one review author onto forms which were tested and refined before use. Microsoft Excel was used to record abbreviated information, specifically on: country where study was conducted; dates of recruitment; participant characteristics (indication, age, sex); inclusion and exclusion criteria; intervention and control content; setting, timing, duration and intensity of intervention; follow up data collection (including loss to follow up); instruments to collect data on pain; outcome data including pain and adverse events. Data extracted were checked against source material by a second review author.

Group means and standard deviations of pain outcome measures were recorded at the longest follow up interval. Proportions of patients with a poor outcome, however defined, were sought. Authors were contacted for unpublished data. Details of adverse events of any type were extracted.

Risk of bias within included studies

Potential sources of bias were assessed using the Cochrane risk of bias tool [32] by two review authors (JD and ADB) working independently; disagreements were adjudicated by a third (VW). We present analyses where possible showing data from all relevant studies for a given outcome, but also conducted sensitivity analyses excluding studies assessed to be at overall high risk of bias.

Data analysis

When studies reported pain outcome data, these were presented and pooled where possible, using Review Manager 5.0 [33]. As the control group in one study [34] was shared by two active interventions, the sample size in meta-analysis was halved for the control group, to preserve independence of findings [17]. Results are presented for one comparison in the

For peer review only - http://bmjopen.bmj.com/site/about/guidelines.xhtml

form of standardised mean differences (SMD), as instruments used to measure outcomes varied.

A random effects model was used. In the event of substantial statistical heterogeneity, it was planned to use a fixed effect model as a sensitivity analysis, but this was not required. A sensitivity analysis excluding those comparisons for which we had imputed data and/or studies we had assessed as being at overall high risk of bias was conducted. At protocol stage, we planned to generate funnel plots, if ten or more studies with similar interventions and controls were identified, as a method of estimating the risk of publication bias. In the event, data were not sufficient.

Heterogeneity between studies (considering both magnitude and direction of effect) was assessed using visual inspection of the graph and the I^2-statistic. We anticipated sources of heterogeneity might include format and intensity of intervention and planned subgroup analyses accordingly, but these were not required.

GRADE approach to evidence

We used the Grading of Recommendations Assessment, Development and Evaluation (GRADE) approach to assess the quality of the evidence [35], specifically study limitations, consistency of effect, imprecision, indirectness and publication bias.

Results

Conduct of the review is summarised in Figure 1. Searches identified 10,968 articles. Of these, 302 potentially relevant reports were considered in detail, of which 101 were identified as RCTs. After detailed evaluation, eight RCTs involving data from 960 participants randomised to nine eligible comparisons were identified.

1. Exercise and education

We identified 76 studies evaluating pre-operative exercise and education. 68 were excluded because investigators had not followed up participants for a minimum of six months after surgery, leaving eight RCTs [34 36-42] involving nine comparisons [34a 34b 36-42].

Seven publications assessed the impact of a single intervention (education (1), exercise (5) or a combination of both exercise and education (1)) against a no-treatment or attentional control. One publication reported relative effects of two different physical interventions (cardio-based exercise or physiotherapy) against a no treatment control. We analysed data from the latter publication as two separate studies, dividing the control group during analysis so as to preserve independence of findings. Details of studies and intervention characteristics are shown in Table 1; risk of bias assessment in Figure 2; meta-analysis and

For peer review only - http://bmjopen.bmj.com/site/about/guidelines.xhtml

sensitivity analyses in Figures 3 to 6; GRADE assessment of quality of evidence for this class of intervention for the outcomes of pain and serious adverse events in Table 2.

Study setting, sample sizes; foci

Included studies were published between 1996 and 2018. Two studies were conducted in Canada[36 42] , two in the USA[34 41], and one each in Belgium[39], Denmark[37], Switzerland[38] and Thailand[40]. Exercise intervention was delivered within clinics or within the home; education was delivered within clinics or online.

Samples at recruitment ranged from 30 to 416. The total number of participants involved in studies of exercise alone was 373; education alone, 416; and exercise and education together, 131.

Sample size calculations were reported by most investigators - but only one was based on the primary outcome used within this review (pain at six months). Other studies had as their main focus satisfaction with TKR[42]; a short-term reduction of 10 points on the HSS and the reduction of hospital stay by one day[34]; the Chair Stand test[38]; a difference of 10 degrees in passive flexion[39]; an 8 point difference in the WOMAC[41]; a 10 point difference on the KOOS ADL subscale[37]. The variability in foci amongst studies makes it difficult for us to estimate a reasonable optimal information size for the review, particularly as some investigators revised their sample size estimates upwards as part of considering limitations of their studies[36 38].

Participants

Five studies exclusively recruited participants undergoing TKR for osteoarthritis[36 38-40 42]; one permitted those with rheumatoid arthritis[34]; and two included participants undergoing either hip or knee replacement for osteoarthritis, with long-term data reported separately[41] or in aggregate[37]. Mean age of participants ranged from 63 to 70 across studies. Gender, where reported, varied greatly, with studies recruiting 17%[40] to 60% women[34].

Interventions

Seven interventions in six studies[34a34b37-41] were exercise-based; one featured an online educational component alone[42]; and one was multifactorial with exercise and an education component focusing on walking with crutches, bed mobility and postoperative range of movement [36].

All exercise interventions included strengthening[34a34b36 38-41], with additional components targeting gait re-education[36], functional exercise[36 38], range of movement[34 36], neuromuscular exercise[38], cardiovascular conditioning[34a41], stretching[34a], flexibility[39 41] and pool-based exercises[41]. The majority of exercise-based interventions required participants to travel to

For peer review only - http://bmjopen.bmj.com/site/about/guidelines.xhtml

clinics or community-based exercise centres but two[39 40] involved exclusively home-based exercise. Staff delivering interventions were, in the majority of cases, physiotherapists.

Participants in the study evaluating both exercise and education received a single session of education (reinforced by provision of a written materials) concerning crutch walking on level ground and stairs, bed mobility and transfers, and post-operative range of motion routines[36]. Those in the study focussing solely on education[42] received a 31 page guide as well as access to an online learning tool during their preadmission consultation, followed by subsequent reminders of their access. The customised e-learning tool included 32 brief (1-2 minute) professionally filmed educational videos presented by either surgeons, therapists, or previous recipients of TKRs, in which expectations related to pain, functional outcomes, limitations, and restrictions were addressed, as well as animations allowing them to visualise the surgery itself.

Intensity, duration, measures of adherence / compliance

Exercise interventions were delivered for between 3 and 12 weeks, 2-7 times a week. The shortest duration of intervention was a three-week home-based intervention[40] which featured the most intense dose (3 sessions per day, 7 days a week). The longest period of intervention (8-12 weeks) took place in the two trials delivering the clinic-based NEMEX intervention[37 38]. Here, variability in duration could be determined by how close to the surgery a patient was at time of recruitment.

With regard to exercise interventions conducted within clinics or at home, investigators typically assessed adherence via logbooks[36-39] recording attendance and sometimes details of achievement and effort. One study of a home-based intervention without formal reporting of adherence featured daily telephone calls to participants[40]; two clinic-based interventions [34a 34b] reported that all participants completed all sessions. The study focussing solely on education[42] assessed the number of overall 'hits' to different educational videos concerning different aspects of the TKR pathway.

Outcomes and outcome data

Six studies reported a joint- or condition- specific patient reported pain outcome measure[36 38 40 41]. In two publications (one reporting data for two separate comparisons), the outcomes reported were surgeon-assessed[34 39].

The latest pain assessment was reported at about six months[34a 34b36 38 39] or 12 months after surgery[40-42]. Measures used were WOMAC pain[36 40], KOOS pain[38 42], SF-36 bodily pain[36 38 41], VAS pain[40], HSS pain score[34a 34b], and KSS[39].

For peer review only - http://bmjopen.bmj.com/site/about/guidelines.xhtml

Data suitable for meta-analysis without imputation on our part were available for three studies[36]. We made robust attempts to obtain data through contact with investigators from studies in which data were provided within graphs without standard deviations[34a 34b 38] in tables with means but without standard deviations[42], as 'non significant' [n.s.] only[39], or as part of aggregated hip and knee data[37]. In three studies, investigators generously provided relevant data in the form of group means and SDs and these data were used[37 38 42]. Following information that data for one study had been permanently lost[34], we measured WOMAC pain data from a graph[34] and, in the absence of standard deviations, used values from a cohort of patients with the same outcome measure reported after knee replacement but converted from a 50 point to a 30 point scale[43]. The first author of exercise-based study which had reported pain findings as simply 'n.s.', responded to our enquiry to indicate that he could no longer access study data.

Risk of bias

Four studies were assessed as being at an overall low risk of bias[36 38 39]. In other studies, we had concerns about potential biases due to differences in baseline patient characteristics in two comparisons [34a 34b] and in one study each, large losses to follow up[41], and issues regarding sequence generation[40].

Effects of intervention: exercise alone

<u>Pain at six months or longer (Figure 3)</u>

Combined results of six eligible studies[34a34b37 38 40 41] indicate no clear effect of intervention on long-term pain (SMD 0.20, 95% CI -0.06 to 0.47; participants = 229). There was no heterogeneity across studies, $Chi^2 = 3.39$, $I^2 = 0\%$.

In a sensitivity analysis restricted to two studies with low risk of bias[36 38], group results provided even less suggestion of difference between groups (SMD 0.08, 95% CI -0.29 to 0.45; participants = 110; $I^2 = 0\%$) (see Figure 4).

Results for one study (n = 122) within this category for which no data were suitable for meta-analysis are in line with the results above, with 'non significant'[39] findings.

Effects of intervention: exercise combined with education

<u>Pain at 6 months or longer (Figure 5)</u>

Results of a single study of exercise with the additional component of one session of education (n = 109) suggest no clear difference between intervention and standard care on long-term pain as assessed by the WOMAC pain scale (MD 2.00, 95% CI -3.45 to 7.45).

For peer review only - http://bmjopen.bmj.com/site/about/guidelines.xhtml

Effects of intervention: education alone

Pain at 6 months or longer (Figure 6)

Results of a single study of education provided by an e-learning tool (n = 319) suggest no clear difference between intervention and standard care in terms of long-term pain as assessed on the KOOS (MD -2.55, 95% CI -6.35 to 1.24) [42].

Adverse events

Peri and post-surgical complications including infections were assessed in most studies; see Table 1. Where reported, these do not appear to have differed between groups. Two studies reported pre-surgical events we judged likely to be intervention-related[38 39]; in each case, participants in an exercise intervention group reported increased pain. No data for this outcome are reported in the study focussing on education alone[42].

2. Oral and injected drugs and iron supplements

Searches identified 12 RCTs of pre-operative oral and injected drugs and iron supplements, of which two reported long-term follow up[44 45]. One evaluated testosterone injections[44] and the other epoetin-α injections[45]. However, a pain outcome was not reported in either study, and thus, we can draw no meaningful conclusions for this class of intervention.

3. Smoking cessation

Searches identified one RCT of smoking cessation with a one year follow up, but outcomes only related to smoking abstinence[46].

4. Bacterial screening and treatment

A screening protocol for staphylococcus aureus and decolonisation with mupirocin nasal ointment and chlorhexidine soap was evaluated in one RCT trial including patients with hip or knee replacement[47]. Participants were followed up at one year for prosthetic joint infection only.

Discussion

Summary of main findings

In people with osteoarthritis receiving TKR, the potential value of interventions to manage pre-operative risk factors or to target care has frequently been assessed, most often focussing on interventions this review has identified (exercise or 'prehabilitation' and/or education with the aim of managing expectations). We found low to moderate quality evidence for no effect on our primary outcome (pain assessed at six months or longer) of either exercise, education or education combined with exercise, and we identified no

For peer review only - http://bmjopen.bmj.com/site/about/guidelines.xhtml

evidence of the effects of any other eligible intervention (e.g. pre-operative weight loss programmes or smoking cessation.

Strengths and limitations of this study

A limitation of our review concerns our focus on prevention of long-term pain only. This decision was based on guidelines which emphasise that osteoarthritic pain which is not controlled by conservative treatments is a primary reason that people undergo TKR[48]. Prevention of long-term pain is a key determinant of patient satisfaction with TKR[49]. Although the primary outcome of many studies we identified was function, pain severity was an important secondary outcome. We cannot draw conclusions on the value of the interventions we have examined from the point of view of (for example) the benefit to individuals or society of quicker short term recovery, as proposed by some triallists[37].

The secondary outcome of this review was adverse events, but reporting was. The poor quality of adverse event reporting in surgical trials is recognised[50], and standardisation is required to improve quality and reduce heterogeneity, particularly in orthopaedics. Although patient reported outcomes are frequently considered to be at high risk of bias, we considered them to be appropriate in this review where questionnaires were completed over six months after a presurgical intervention.

We also excluded studies which continued beyond the period of the TKR procedure; a separate review considering studies including interventions which combined pre-habilitation and post-TKR rehabilitation may well be justified.

This review in context

This is the first review in nearly a decade[51] which takes as its focus the preoperative period itself, rather than aiming at a class of interventions; the evidence base, however, remains limited.

Numerous reviews have appeared focusing on outcomes relating to pre-operative function and pain, the in-hospital experience, and long-term recovery, specifically following pre-operative exercise and/or education programmes involving joint replacement [52 53 54-67 68 69] Findings vary based on the outcomes and timeframes prioritised by review authors. In general, those concentrating on short term function and/or outcomes related to discharge report more positive findings as regards implications for both practice and future research [68] Those considering the longer term are less sanguine. One review which evaluated the effects of 'prehabilitation' across *all* surgical patients concluded that in "*prehabilitation studies are not recommended in patients with osteoarthritis for whom arthroplasty is planned"* [63]. Subsequently, amongst the most comprehensive of systematic reviews

For peer review only - http://bmjopen.bmj.com/site/about/guidelines.xhtml

conducted in arthroplasty alone is that by Wang and colleagues. They concluded that the effects of prehabilitation on long-term and indeed many short-term outcomes were too small to be considered clinically important [65].

Our review adds to the existing evidence base as it includes two trials not included in others [38 42]. With broad inclusion criteria we aimed to identify diverse interventions conducted in the pre-operative period. Important issues that our review benefits from are preregistration of the study protocol to limit reporting bias, conduct of study according to PRISMA guidelines and comprehensive up to date searches of a range of appropriate literature databases. Experienced systematic reviewers undertook screening of articles, data extraction and risk of bias assessment in duplicate. Author contact was extensive and with the help of study investigators, we included data from studies which had been excluded from previous meta-analyses or had been included with estimates for variances. Furthermore, use of GRADE methodology allowed us to assess the overall quality of the evidence.

Implications for practice and research

There are several possible explanations for the observed lack of a convincing effect of pre-operative interventions on long-term pain after TKR reported within this review. All pre-operative interventions conducted within an undifferentiated general population may appear ineffective in the long-term, as for many patients, TKR surgery is highly effective in reducing long-term pain. To detect an effect of intervention for the small but important minority of patients for whom TKR is not highly effective in reducing long-term pain, studies may need either to be much larger, or more focussed on particular patient subgroups. Despite the fact that patients report that appropriate and realistic support before surgery can help them achieve a long-term positive experience of surgery[70], it is acknowledged that it is difficult to recruit patients to randomised controlled trials before surgery and participants in existing trials may have been highly selected. Patients may have exhausted conservative strategies before surgery and, in evaluating new approaches, there may be an unwillingness among patients to participate in randomised trials[11]. Intervention content may have focused on improving pre-operative physical function and preparing patients for their hospital admission and recovery without specific reference or ‘flags’ to help identify participants whose recovery did not follow the norm. Interventions that we included may have focused on aspects of care that do not have a sustained effect on pain. For example, it could be argued that the only outcome necessary in an evaluation of bacterial screening and targeted treatment is the incidence of surgical site infections; however, superficial and particularly deep infections after knee replacement are associated with high levels of morbidity including pain. Patient reported pain and function are acknowledged as key outcomes in studies of infection in joint

For peer review only - http://bmjopen.bmj.com/site/about/guidelines.xhtml

replacement. Studies may have led to other benefits (including quicker recovery) which are not reported here, with our focus on long-term pain.

Aspects of pre-surgical care with a possible link with chronic pain which are as yet unsupported by relevant randomised trials include comorbidity management, pain management, nutritional supplements, and weight loss. Targets for psychological support are also merit research; e.g. to reduce pain catastrophizing, which has been implicated as a risk factor for chronic pain[71]; however, a recent trial where intervention began in the pre-surgical period and extended well beyond it found that pain coping skills training for people with high pain catastrophizing did not improve WOMAC pain scores at 12 months after TKR [72]. For other pre-operative strategies, such as smoking cessation, weight loss and glucose management, there may be tenuous, if any, mechanisms to link with long-term pain outcomes. However, they share a common aim in preparing patients for surgery, recovery and rehabilitation, and preventing adverse events that may limit the potential benefit of knee replacement, so we believe it right to advocate that studies investigating their effects incorporate long term follow-up.

Conclusion

Pre-surgical interventions have been evaluated in patients receiving TKR. We found low to moderate quality evidence to suggest that neither exercise, education nor a combination of both is effective in preventing long-term pain in adults scheduled to receive total knee replacement and it is difficult to recommend further research except of novel approaches and/or in specific populations. However, studies of pre-operative interventions with long-term follow up after surgery are feasible in other areas and for them, adequately powered, randomised controlled trials, planned and reported according to CONSORT standards, should be conducted to identify clinically effective pre-operative treatments to help patients achieve a long-term pain-free outcome after knee replacement.

Acknowledgements

We gratefully acknowledge unpublished outcome data from the following investigators: Dr Sharon Culliton, Dr Erika Huber and Dr Allan Villadsen. We also are thankful for confirmation concerning other study data from Professor Darryl D'Lima and Dr Fabrizio Matassi. We are also thankful for translation of Yunfei Li, University of Bristol, for his help in translating a paper from Chinese.

Author contributions

All authors contributed to the concept and design of the study. JD, ADB and VW contributed to the acquisition and analysis of data. JD and ADB drafted the article and VW, RG-H and

For peer review only - http://bmjopen.bmj.com/site/about/guidelines.xhtml

AWB revised it critically for important intellectual content. JD and ADB take responsibility for the integrity of the work as a whole, from inception to finished article.

Funding

This study is funded by the National Institute for Health Research (NIHR) [NIHR Programme Grant for Applied Research (Grant Reference Number RP-PG-0613-20001)]. This study was also supported by the NIHR Biomedical Research Centre at the University Hospitals Bristol NHS Foundation Trust and the University of Bristol. The views expressed in this publication are those of the authors and not necessarily those of the NHS, the National Institute for Health Research or the Department of Health and Social Care.

Disclaimer

The views expressed are those of the authors and not necessarily those of the NHS, the NIHR or the Department of Health. The funder had no involvement in the study design, data collection, data analysis, interpretation of data or writing of the manuscript.

Competing interests

No author has any direct conflict of interest with the published work above. Outside of this work, VW and AWB are co-applicants on an institutional grant from Stryker for a study evaluating the outcomes of the Triathlon knee replacement.

Patient consent

Not required.

Data sharing statement

Not applicable

For peer review only

For peer review only - http://bmjopen.bmj.com/site/about/guidelines.xhtml

Table 1. Characteristics of studies evaluating pre-operative exercise and education

Publication<br>Location<br>Date of study	Indication<br>Number randomised (intervention: control)<br>Mean age (% female)	Primary focus of intervention<br>Study setting<br>Timing, duration and intensity<br>Control group care	Longest follow up after surgery<br>Losses to follow up (intervention: control)<br>Risk of bias<br>Outcomes
Beaupre et al, 2004<br>Canada<br>Date not specified	Non-inflammatory arthritis<br>N= 131 (65:66)<br>67 years (55%)	Multifactorial: exercise (gait re-education, functional, ROM, strengthening) and education on crutch walking, bed mobility and postoperative ROM<br>Community physical therapy clinic group<br>3-times per week for 4-weeks, 6 weeks before surgery<br>Control: no intervention	6 and 12 months<br>16 (10:6) patients cancelled surgery, 6 (4:2) patients lost to 12 month follow up<br>Low overall risk of bias<br>No difference in WOMAC pain or SF-36 bodily pain at 6 and 12 months (p>0.05)<br>General complications, “similarly dispersed across both groups”: Pulmonary embolism (2:2), DVT (3:6), Superficial infection (2:3), Deep infection (1:0), Manipulation (1:2)
Culliton et al. 2018<br>Canada<br>2013-2014	Osteoarthritis<br>N= 416 (207:209)<br>64 years (43%)	Education: access to an online e-learning tool during pre-admission visit + same 31-page guide provided to the control group. Access to e-learning tool remained active until the study end (1 yr postop). Tool consisted of custom-made 32 videos (1-2 mins) addressing patient expectations, presented by surgeons, physical therapists and patients<br>Control: Booklet alone	12 months post-operative<br>Data analysed are 76.6 % of original sample randomised. Reasons include (post PAC meeting) TKR not taking place or lack of email address. Later exclusions due to failure to submit data.<br>Low overall risk of bias<br>1 year postoperative, significant between-group differences in favour of the control group for the new KOOS symptoms score (P= 0.04) overall. Pain scores obtained from authors directly were not significantly different between groups<br>AEs apparently not sought
D’Lima et al. 1996<br>Cardiovascular conditioning<br>USA<br>Date not specified	Rheumatoid or osteoarthritis<br>N=20 (10:10)<br>70.6 years (35%)	Exercise (stretching, strengthening, cardiovascular conditioning)<br>Hospital. Individualised<br>45 minute sessions, 3 times a week for 18 weeks commencing 6 weeks before surgery	24 and 48 weeks<br>No losses to follow up reported<br>High risk of bias: small numbers of patients; baseline demographic differences; failure to report data other than graphically<br>Hospital for Special Surgery Knee HSS pain score improved in intervention group compared with

For peer review only - http://bmjopen.bmj.com/site/about/guidelines.xhtml

		Control: one meeting with physical therapist	control but not statistically significant<br>Sleep apnoea (1:0), Manipulation (1:0), Deep infection (0:1), Intestinal pseudoobstruction (0:1)
D'Lima et al. 1996<br>Physical therapy<br>USA<br>Date not specified	Rheumatoid or osteoarthritis<br>N=20 (10:10)<br>69.0 years (60%)	Exercise (strengthening, ROM)<br>Hospital. 1 on 1 programme<br>45 minute sessions, 3 times a week for 18 weeks commencing 6 weeks before surgery<br>Controls: one meeting with physical therapist	24 and 48 weeks<br>No losses to follow up reported<br>High risk of bias: small numbers of patients; baseline demographic differences<br>Hospital for Special Surgery Knee Rating pain score improved in intervention group compared with control but not statistically significant<br>Atrial fibrillation (1:0), Paroxysmal tachycardia (1:0), Deep infection (0:1), Intestinal pseudo-obstruction (0:1)
Fernandes et al. 2017<br>Denmark<br>2010-2011<br>1 hospital	Osteoarthritis<br>165 (84; 81) hip or knee replacement<br>Mean 67.9 (SD 8.6); 66.9 (8.3)<br>(56%)	Exercise (neuromuscular focusing on lower extremity muscular control and quality of movement)<br>Hospital: group-based, physiotherapist led<br>Controls: standard care<br>Mean 13.1 one-hour sessions twice per week during 8 weeks before surgery.	61 weeks<br>2; 5<br>Low risk of bias<br>Mean differences at 61 weeks favoured the exercise group for all KOOS subscales, but only significantly for quality of life subscale. Disaggregated data no longer available for as in previous papers; acquired by personal contact<br>0; 2 deep infection
Huber et al. 2015<br>Switzerland<br>2009-2012	Osteoarthritis<br>N=45 (22:23)<br>70.4 years (47%)	Exercise (neuromuscular including functional and strengthening)<br>Hospital: group based<br>8 or more sessions commencing 6-12 weeks before surgery.<br>Controls: all patients attended "knee school" on 3 occasions from about 4 weeks before surgery	12 months<br>9 (5:4) lost to follow up<br>Low risk of bias<br>No difference in KOOS pain or SF-36 bodily pain between groups at 12 months<br>1 intervention patient had a joint specific adverse event
Matassi et al. 2014<br>Belgium<br>2005-2006	Osteoarthritis<br>N=122 (61:61)<br>66.5 years (48%)	Exercise (muscle strength and flexibility)<br>Home<br>5 days per week for 6 weeks commencing 6 weeks before surgery<br>Controls: maintained regular activities	1 year<br>No losses to follow up reported<br>High risk of bias due to SOR; outcome also not PROM<br>'n.s' effect of exercise on the Knee Society Clinical Rating System knee score or patient function score

For peer review only - http://bmjopen.bmj.com/site/about/guidelines.xhtml

			Manipulation (5:3). In the intervention group 1 patient developed ipsilateral adductor tendinitis and 1 patient stopped exercises due to increased knee pain
Rooks et al. 2006<br>USA<br>2001-2003	Osteoarthritis<br>N=45 (22:23)<br>67.0 years (53%)	Exercise (strengthening, flexibility, cardiovascular, pool exercises)<br>Community fitness facility<br>3 times per week for 6 weeks 30-60 mins each increasing intensity from 4-6 weeks<br>Controls: 2 education mailings and 3 telephone calls	26 weeks<br>16 (8:8) lost to follow up<br>High risk of bias: large loss to follow up<br>Statistically significant greater improvement in SF-36 bodily pain at 26 weeks ($p<0.05$) but no difference for WOMAC pain<br>Perioperative complications (3:4). No serious adverse events in either group requiring hospital admission
Tungtrongjit et al. 2012<br>Thailand<br>2011-2012	Osteoarthritis<br>N=68 (30:30 in analyses)<br>64.5 years (17%)	Exercise (quadriceps strengthening)<br>Home<br>3 times per day for 3 weeks commencing 3 weeks before surgery<br>Controls: asked to continue normal activities	6 months<br>8 patients excluded from analyses due to adverse events and loss to follow up<br>Possible risk of bias unclear sequence generation; : data provided only o patients followed up<br>WOMAC pain improved in intervention compared with controls ($p= 0.029$) but not VAS pain ($p=0.137$)<br>Across all randomised patients there was 1 surgical wound infection, 1 post-operative knee trauma, and 2 post-operative wound dehiscences

WOMAC Western Ontario and McMaster Universities Osteoarthritis Index; SF-36 Short Form 36; VAS Visual analogue scale; KOOS Knee disability and Osteoarthritis Outcome Score; EQ-5D European Quality of Life-5 Dimensions

For peer review only

Table 2

Summary of findings: Exercise alone compared to standard care for knee replacement (long-term outcome only)

Patient or population: Adult patients scheduled for total knee replacement
Intervention: 3- 12 weeks exercise conducted in the presurgical period
Setting: Clinic-supervised exercise (5 studies); home-based exercise (2 studies)
Comparison: Standard care

Outcome<br>№ of participants<br>(studies)	Relative effect (95%CI)	**Illustrative comparative risks (95% CI)**		Certainty	Comments
**Long-term pain**<br><br>Assessed with: Various scales (WOMAC[a], KOOS[b], HSSK[c])<br>Follow up: range 6 months to 12 months<br><br>№ of participants in metaanalysis: 229 (6 RCTs)<br><br>№ of participants not in metaanalysis: 122 (1 RCT).	**Metaanalysis**:<br><br>SMD **0.2 higher** (-0.06 - 0.47)<br><br>**Narrative**<br><br>"Non significant difference"	Mean KOOS scores in the untreated group ranged from 73.2 to 85.95 in the control group; mean HSSK scores in the control group were 27; mean WOMAC scores in control group were<br><br>-2.3 to -4.8	The mean level of pain after exercise was 0.20 standard deviations lower (-0.06 to 0.47 lower).	⊕⊕⊕◯<br>MODERATE [d,e]	There was no clear evidence of a difference between exercise and standard care for long-term pain either from data from the metaanalysis or from a comparatively large study not included within the metaanalysis.<br><br>We rescaled the SMD finding to a commonly used instrument in the field - the KOOS, using estimations based on the mean of a representative study within the analysis (Huber 2015) and our pooled SMD of 0.02 (-0.06 to 0.47 higher). This indicates that exercise led to a mean increase of 2.7 units/points on the KOOS (95% CI -0.8 to 6.29) in the treated group.<br><br>Sensitivity analysis of two studies at low risk of bias suggested a smaller effect (mean increase of 1.46 points on the KOOS (95% CI-4.58 to 7.50)
**Adverse events**	See comment	See comment	See comment	⊕⊕◯◯<br>LOW [d,f]	Two studies reported treatment-related adverse events (increase in pain) separately from the main pain outcomes, which occurred during the period of intervention. Information on perioperative complications e.g. superficial and deep infections, were reported in all studies. Events were few and attribution to treatment status difficult

CI: Confidence interval; **MD:** Mean difference; **SMD:** Standardised mean difference

For peer review only - http://bmjopen.bmj.com/site/about/guidelines.xhtml

Summary of findings: Exercise alone compared to standard care for knee replacement (long-term outcome only)

Patient or population: Adult patients scheduled for total knee replacement
Intervention: 3- 12 weeks exercise conducted in the presurgical period

Setting: Clinic-supervised exercise (5 studies); home-based exercise (2 studies)
Comparison: Standard care

Outcome<br>№ of participants<br>(studies)	Relative effect (95%CI)	Illustrative comparative risks (95% CI)		Certainty	Comments

GRADE Working Group grades of evidence
High certainty: We are very confident that the true effect lies close to that of the estimate of the effect
Moderate certainty: We are moderately confident in the effect estimate: The true effect is likely to be close to the estimate of the effect, but there is a possibility that it is substantially different
Low certainty: Our confidence in the effect estimate is limited: The true effect may be substantially different from the estimate of the effect
Very low certainty: We have very little confidence in the effect estimate: The true effect is likely to be substantially different from the estimate of effect

Explanations

a. WOMAC = The Western Ontario and McMaster Universities Osteoarthritis Index (WOMAC) (Bellamy et al 1988). The original pain scale runs from 0 to 20 and a higher number indicates a greater pain. There are several modifications. Some triallists also reverse the polarity and/or transform responses into a 0-100 scale where a high number indicates less pain. We standardised to this latter method as it was most common and was similar to the KOOS and the HSSK.
b. KOOS = Knee Injury and Osteoarthritis Outcome Score (Roos et al 1998) . The scale ranges from 0 – 100 and a higher number indicates less pain
c. HSSK = Hospital Society Score (Insall et al 1976). Pain is measured on a scale of 0 to 30, with a high number indicating less pain.
d. Downgraded due to risk of bias associated with lack of blinding of both personnel and participants in all studies also concerns about sequence generation /allocation concealment in three studies.
e. We did not downgrade for imprecision as findings of the one large study not included in metaanalysis were very similar to the six studies included in metaanalysis
f. We downgraded the quality of evidence for study limitations, in particular imprecision: estimate based on few events

For peer review only

For peer review only - http://bmjopen.bmj.com/site/about/guidelines.xhtml

Summary of findings: Exercise & education compared to standard care for knee replacement (long-term outcome only)

Patient or population: Adult patients scheduled for total knee replacement
Intervention: 1 session of education combined with 4 weeks' exercise in the pre-operative period
Setting: Clinic-supervised exercise Clinics (1 study)
Comparison: TAU

Outcome<br>№ of participants<br>(studies)	Relative effect (95%CI)	**Illustrative comparative risks (95% CI)**		Certainty	Comments
**Long-term pain**<br>Assessed with: WOMAC[a] scale<br>Follow up: 12 months<br>№ of participants: 109<br>(1 RCT)	**Single study results**:<br>MD **2 units higher**<br>(3.45 lower to 7.45 higher) -	Mean score of untreated control group was 80 on the WOMAC (sd = 16)	Exercise and education resulted in a mean score of 82 on the WOMAC (sd= 13)	⊕⊕◯◯<br>LOW [b,c,d]	)
**Adverse events**	See comment	See comment	See comment	⊕⊕⊕◯<br>VERY LOW [b,c,d,e]	General complications, "similarly dispersed across both groups": Pulmonary embolism (2:2), DVT (3:6), Superficial infection (2:3), Deep infection (1:0), Manipulation (1:2)

CI: Confidence interval; **MD:** Mean difference

GRADE Working Group grades of evidence
High certainty: We are very confident that the true effect lies close to that of the estimate of the effect
Moderate certainty: We are moderately confident in the effect estimate: The true effect is likely to be close to the estimate of the effect, but there is a possibility that it is substantially different
Low certainty: Our confidence in the effect estimate is limited: The true effect may be substantially different from the estimate of the effect
Very low certainty: We have very little confidence in the effect estimate: The true effect is likely to be substantially different from the estimate of effect

Explanations

a. WOMAC = The Western Ontario and McMaster Universities Osteoarthritis Index (WOMAC) (Bellamy et al 1988). The original pain scale runs from 0 to 20 and a higher number indicates a greater pain. There are several modifications. Some triallists also reverse the polarity and/or transform responses into a 0-100 scale where a high number indicates less pain. We standardised to this latter method as it was most common and was similar to the KOOS and the HSSK.
b. Downgraded due to risk of bias due to lack of blinding of participants or personnel.
c. Downgraded for imprecision due to being one study with less than an optimal information sample size
d. There were too few studies to reliably assess risk of publication bias or other sources of small study bias
e. We downgraded the quality of evidence for study limitations, in particular imprecision: estimate based on few events

For peer review only - http://bmjopen.bmj.com/site/about/guidelines.xhtml

Summary of findings: Exercise & education compared to standard care for knee replacement (long-term outcome only)

Patient or population: Adult patients scheduled for total knee replacement
Intervention: 1 session of education combined with 4 weeks' exercise in the pre-operative period
Setting: Clinic-supervised exercise Clinics (1 study)
Comparison: TAU

Outcome № of participants (studies)	Relative effect (95%CI)	Illustrative comparative risks (95% CI)		Certainty	Comments

Summary of findings: Education compared to standard care for knee replacement (long term outcome only)

Patient or population: Adult patients scheduled for total knee replacement
Intervention: Access to online elearning tool with active demonstration at presurgical visit followed by email prompts
Setting: Clinic followed by online access (1 study)
Comparison: TAU

Outcome № of participants (studies)	Relative effect (95%CI)	Illustrative comparative risks (95% CI)		Certainty	Comments
**Longterm pain**<br>Assessed with: KOOS scale[a]<br>Follow up: 12 months<br>№ of participants: 319<br>(1 RCT)	**Single study results**:<br>MD **2.55 lower**<br>(6.35 lower to 1.24 higher)	Mean score of untreated control group was 82.62 on the KOOS (sd = 15.4)	Exercise and education resulted in a decrease on the KOOS, leading to a mean score in the treated group of 80.01(sd= 19)	⊕⊕⊕◯<br>MODERATE [b,c]	
**Adverse events**	See comment	See comment	See comment		No data on adverse events appear to have been sought within the one study within this comparison

CI: Confidence interval; **MD:** Mean difference

GRADE Working Group grades of evidence
High certainty: We are very confident that the true effect lies close to that of the estimate of the effect
Moderate certainty: We are moderately confident in the effect estimate: The true effect is likely to be close to the estimate of the effect, but there is a possibility that it is substantially different
Low certainty: Our confidence in the effect estimate is limited: The true effect may be substantially different from the estimate of the effect
Very low certainty: We have very little confidence in the effect estimate: The true effect is likely to be substantially different from the estimate of effect

For peer review only - http://bmjopen.bmj.com/site/about/guidelines.xhtml

Summary of findings: Exercise & education compared to standard care for knee replacement (long-term outcome only)

Patient or population: Adult patients scheduled for total knee replacement
Setting: Clinic-supervised exercise Clinics (1 study)
Intervention: 1 session of education combined with 4 weeks' exercise in the pre-operative period
Comparison: TAU

Outcome<br>№ of participants<br>(studies)	Relative effect (95%CI)	Illustrative comparative risks (95% CI)		Certainty	Comments

Explanations
a. KOOS = Knee Injury and Osteoarthritis Outcome Score (Roos et al 1998) . The scale ranges from 0 – 100 and a higher number indicates less pain
b. Downgraded due to risk of bias due to lack of blinding of participants or personnel and high loss to followup (24%)
c. There were too few studies to reliably assess risk of publication bias or other sources of small study bias

For peer review only - http://bmjopen.bmj.com/site/about/guidelines.xhtml

References

1. McAlindon TE, Bannuru RR, Sullivan MC, et al. OARSI guidelines for the non-surgical management of knee osteoarthritis. *Osteoarthritis and cartilage* 2014;22(3):363-88. doi: 10.1016/j.joca.2014.01.003 [published Online First: 2014/01/28]
2. Osteoarthritis: care and management. NICE clinical guideline [CG177]. London: National Institute for Health and Clinical Excellence, 2014.
3. National Joint Registry for England, Wales, Northern Ireland and the Isle of Man: 15th Annual Report. Hemel Hempstead: NJR Centre 2018.
4. Scottish Arthroplasty Project. Annual report 2018. Edinburgh: NHS National Services Scotland 2018.
5. Maradit Kremers H, Larson DR, Crowson CS, et al. Prevalence of total hip and knee replacement in the United States. *The Journal of bone and joint surgery American volume* 2015;97(17):1386-97. doi: 10.2106/jbjs.N.01141 [published Online First: 2015/09/04]
6. Löfvendahl S, Bizjajeva S, Ranstam J, et al. Indications for hip and knee replacement in Sweden. *J Eval Clin Pract* 2011;17(2):251-60. doi: 10.1111/j.1365-2753.2010.01430.x
7. Beswick AD, Wylde V, Gooberman-Hill R, et al. What proportion of patients report long-term pain after total hip or knee replacement for osteoarthritis? A systematic review of prospective studies in unselected patients. *BMJ Open* 2012;2(1):e000435.
8. Nilsdotter AK, Toksvig-Larsen S, Roos EM. Knee arthroplasty: are patients' expectations fulfilled? A prospective study of pain and function in 102 patients with 5-year follow-up. *Acta Orthop* 2009;80(1):55-61. doi: http://dx.doi.org/10.1080/17453670902805007
9. Scott CE, Howie CR, MacDonald D, et al. Predicting dissatisfaction following total knee replacement: a prospective study of 1217 patients. *The Journal of bone and joint surgery British volume* 2010;92(9):1253-8. doi: http://dx.doi.org/10.1302/0301-620x.92b9.24394 [published Online First: 2010/08/28]
10. Sayers A, Wylde V, Lenguerrand E, et al. Rest pain and movement-evoked pain as unique constructs in hip and knee replacements. *Arthritis Care Res* 2016;68(2):237-45.
11. Blom AW, Artz N, Beswick AD, et al. Improving patients' experience and outcome of total joint replacement: The RESTORE programme. *Programme Grants Appl Res* 2016;4(12) [published Online First: in press]
12. Sullivan MJ, Thorn B, Haythornthwaite JA, et al. Theoretical perspectives on the relation between catastrophizing and pain. *The Clinical journal of pain* 2001;17(1):52-64. [published Online First: 2001/04/06]
13. McDonald S, Hetrick S, Green S. Pre-operative education for hip or knee replacement. *Cochrane Database Syst Rev* 2004(1):CD003526.
14. Wylde V, Dennis J, Gooberman-Hill R, et al. Effectiveness of postdischarge interventions for reducing the severity of chronic pain after total knee replacement: systematic review of randomised controlled trials. *BMJ Open* 2018;8(2):e020368. doi: https://dx.doi.org/10.1136/bmjopen-2017-020368
15. Beswick A, Dennis J, Gooberman-Hill R, et al. The effectiveness of pre-, peri- and post-operative interventions in preventing chronic pain in patients receiving primary total knee replacement: a systematic review. *PROSPERO* 2017
16. Richardson WS, Wilson MC, Nishikawa J, et al. The well-built clinical question: a key to evidence-based decisions. *ACP journal club* 1995;123(3):A12-3. [published Online First: 1995/11/01]
17. Higgins JPT, Green S. Cochrane Handbook for Systematic Reviews of Interventions Version 5.1.0 [Updated March 2011]: The Cochrane Collaboration, 2011.
18. Moher D, Liberati A, Tetzlaff J, et al. Preferred reporting items for systematic reviews and meta-analyses: the PRISMA statement. *J Clin Epidemiol* 2009;62(10):1006-12.

For peer review only - http://bmjopen.bmj.com/site/about/guidelines.xhtml

19. Gooberman-Hill R, Burston A, Clark E, et al. Involving patients in research: considering good practice. *Musculoskeletal Care* 2013;11(4):187-90. doi: 10.1002/msc.1060 [published Online First: 2013/12/07]
20. Murray DW, Fitzpatrick R, Rogers K, et al. The use of the Oxford hip and knee scores. *J Bone Joint Surg Br* 2007;89(8):1010-4. doi: http://dx.doi.org/10.1302/0301-620X.89B8.19424 [published Online First: 2007/09/06]
21. Bellamy N, Buchanan W, Goldsmith C, et al. Validation study of WOMAC: a health status instrument for measuring clinically-important patient-relevant outcomes following total hip or knee arthroplasty in osteoarthritis. *J Orthop Rheumatol* 1988;1
22. Roos EM, Roos HP, Lohmander LS, et al. Knee Injury and Osteoarthritis Outcome Score (KOOS)--development of a self-administered outcome measure. *J Orthop Sports Phys Ther* 1998;28(2):88-96.
23. Insall JN, Dorr LD, Scott RD, et al. Rationale of the Knee Society clinical rating system. *Clin Orthop Relat Res* 1989(248):13-4.
24. Insall JN, Ranawat CS, Aglietti P, et al. A comparison of four models of total knee-replacement prostheses. *J Bone Joint Surg Am* 1976;58(6):754-65. [published Online First: 1976/09/01]
25. Wylde V, Bruce J, Beswick A, et al. The assessment of chronic post-surgical pain after knee replacement: A systematic review. *Arthritis Care Res* 2013;65(11):1795-803. doi: http://dx.doi.org/10.1002/acr.22050 [published Online First: 2013/06/07]
26. Hewlett SA. Patients and clinicians have different perspectives on outcomes in arthritis. *J Rheumatol* 2003;30(4):877-9.
27. Bachmeier CJM, March LM, Cross MJ, et al. A comparison of outcomes in osteoarthritis patients undergoing total hip and knee replacement surgery. *Osteoarthritis and cartilage* 2001;9(2):137-46.
28. Ware JE, Jr., Sherbourne CD. The MOS 36-item short-form health survey (SF-36). I. Conceptual framework and item selection. *Med Care* 1992;30(6):473-83.
29. Ware J, Jr., Kosinski M, Keller SD. A 12-Item Short-Form Health Survey: construction of scales and preliminary tests of reliability and validity. *Med Care* 1996;34(3):220-33.
30. Dworkin RH, Turk DC, Farrar JT, et al. Core outcome measures for chronic pain clinical trials: IMMPACT recommendations. *Pain* 2005;113(1-2):9-19. doi: 10.1016/j.pain.2004.09.012
31. Moore RA, Eccleston C, Derry S, et al. "Evidence" in chronic pain--establishing best practice in the reporting of systematic reviews. *Pain* 2010;150(3):386-9. doi: 10.1016/j.pain.2010.05.011 [published Online First: 2010/07/16]
32. Higgins JPT, Altman DG, Gøtzsche PC, et al. The Cochrane Collaboration's tool for assessing risk of bias in randomised trials. *BMJ* 2011;343:d5928. doi: 10.1136/bmj.d5928 %J BMJ
33. Review Manager (RevMan) [Computer program]. Version 5.3 [program]. Copenhagen: The Nordic Cochrane Centre: The Cochrane Collaboration, 2014.
34. D'Lima DD, Colwell CW, Jr., Morris BA, et al. The effect of preoperative exercise on total knee replacement outcomes. *Clin Orthop Relat Res* 1996(326):174-82.
35. Guyatt G, Oxman AD, Akl EA, et al. GRADE guidelines: 1. Introduction-GRADE evidence profiles and summary of findings tables. *J Clin Epidemiol* 2011;64(4):383-94. doi: 10.1016/j.jclinepi.2010.04.026 [published Online First: 2011/01/05]
36. Beaupre LA, Lier D, Davies DM, et al. The effect of a preoperative exercise and education program on functional recovery, health related quality of life, and health service utilization following primary total knee arthroplasty. *J Rheumatol* 2004;31(6):1166-73.
37. Fernandes L, Roos E, Overgaard S, et al. Supervised neuromuscular exercise prior to hip and knee replacement: 12-month clinical effect and cost-utility analysis alongside a randomised controlled trial. *BMC Musculoskelet Disord* 2017;18(1):5. doi: 10.1186/s12891-016-1369-0
38. Huber EO, Roos EM, Meichtry A, et al. Effect of preoperative neuromuscular training (NEMEX-TJR) on functional outcome after total knee replacement: an assessor-blinded randomized controlled trial. *BMC Musculoskelet Disord* 2015;16(101)

For peer review only - http://bmjopen.bmj.com/site/about/guidelines.xhtml

39. Matassi F, Duerinckx J, Vandenneucker H, et al. Range of motion after total knee arthroplasty: the effect of a preoperative home exercise program. *Knee Surg Sports Traumatol Arthrosc* 2014;22(3):703-9.
40. Tungtrongjit Y, Weingkum P, Saunkool P. The effect of preoperative quadriceps exercise on functional outcome after total knee arthroplasty. *J Med Assoc Thai* 2012;95 Suppl 10:S58-66.
41. Rooks DS, Huang J, Bierbaum BE, et al. Effect of preoperative exercise on measures of functional status in men and women undergoing total hip and knee arthroplasty. *Arthritis Rheum* 2006;55(5):700-8.
42. Culliton SE, Bryant DM, MacDonald SJ, et al. Effect of an e-Learning Tool on Expectations and Satisfaction Following Total Knee Arthroplasty: A Randomized Controlled Trial. *Journal of Arthroplasty* 2018;33(7):2153-58. doi: http://dx.doi.org/10.1016/j.arth.2018.02.040
43. Jacobs CA, Christensen CP. Correlations between Knee Society function scores and functional force measures. *Clin Orthop Relat Res* 2009;467(9):2414-19. doi: 10.1007/s11999-009-0811-0
44. Amory JK, Chansky HA, Chansky KL, et al. Preoperative supraphysiological testosterone in older men undergoing knee replacement surgery. *J Am Geriatr Soc* 2002;50(10):1698-701.
45. Moonen AFCM, Thomassen BJW, Knoors NT, et al. Pre-operative injections of epoetin-alpha versus post-operative retransfusion of autologous shed blood in total hip and knee replacement: a prospective randomised clinical trial. *The Journal of bone and joint surgery British volume* 2008;90(8):1079-83.
46. Villebro NM, Pedersen T, Moller AM, et al. Long-term effects of a preoperative smoking cessation programme. *Clin Respir J* 2008;2(3):175-82.
47. Sousa RJG, Barreira PMB, Leite PTS, et al. Preoperative Staphylococcus aureus Screening/Decolonization Protocol Before Total Joint Arthroplasty-Results of a Small Prospective Randomized Trial. *J Arthroplasty* 2016;31(1):234-9.
48. Gademan MGJ, Hofstede SN, Vliet Vlieland TPM, et al. Indication criteria for total hip or knee arthroplasty in osteoarthritis: a state-of-the-science overview. *BMC Musculoskelet Disord* 2016;17(1):463.
49. Hamilton DF, Lane JV, Gaston P, et al. What determines patient satisfaction with surgery? A prospective cohort study of 4709 patients following total joint replacement. 2013;3(4):e002525. doi: 10.1136/bmjopen-2012-002525 %J BMJ Open
50. Wylde V, Dennis J, Beswick AD, et al. Systematic review of management of chronic pain after surgery. *Br J Surg* 2017;104(10):1293-306. doi: 10.1002/bjs.10601 [published Online First: 2017/07/07]
51. Wallis JA, Taylor NF. Pre-operative interventions (non-surgical and non-pharmacological) for patients with hip or knee osteoarthritis awaiting joint replacement surgery--a systematic review and meta-analysis. *Osteoarthritis Cartilage* 2011;19(12):1381-95. doi: 10.1016/j.joca.2011.09.001 [published Online First: 2011/10/01]
52. Ackerman IN, Bennell KL. Does pre-operative physiotherapy improve outcomes from lower limb joint replacement surgery? A systematic review. *Aust J Physiother* 2004;50(1):25-30. [published Online First: 2004/02/28]
53. Valkenet K, van de Port IG, Dronkers JJ, et al. The effects of preoperative exercise therapy on postoperative outcome: a systematic review. *Clin Rehabil* 2011;25(2):99-111. doi: 10.1177/0269215510380830 [published Online First: 2010/11/10]
54. Hoogeboom TJ, Oosting E, Vriezekolk JE, et al. Therapeutic validity and effectiveness of preoperative exercise on functional recovery after joint replacement: a systematic review and meta-analysis. *PLoS One* 2012;7(5):e38031. doi: 10.1371/journal.pone.0038031 [published Online First: 2012/06/08]
55. Silkman Baker C, McKeon JM. Does preoperative rehabilitation improve patient-based outcomes in persons who have undergone total knee arthroplasty? A systematic review. *PM R* 2012;4(10):756-67. doi: 10.1016/j.pmrj.2012.06.005 [published Online First: 2012/10/25]

For peer review only - http://bmjopen.bmj.com/site/about/guidelines.xhtml

56. Gill SD, McBurney H. Does exercise reduce pain and improve physical function before hip or knee replacement surgery? A systematic review and meta-analysis of randomized controlled trials. *Arch Phys Med Rehabil* 2013;94(1):164-76. doi: 10.1016/j.apmr.2012.08.211 [published Online First: 2012/09/11]
57. Shoemaker MJ, Gibson C, Saagman S. Preoperative Exercise in Individuals Undergoing Total Knee Arthroplasty State of the Evidence. *Top Geriatr Rehabil* 2013;29(1):2-16. doi: 10.1097/TGR.0b013e318275c214
58. Simmons L ST. Effectiveness of pre-operative physiotherapy-based programmes on outcomes following total knee arthroplasty: a systematic review and meta-analysis. *Physical Therapy Reviews* 2013;18(1):1-10.
59. Jordan RW, Smith NA, Chahal GS, et al. Enhanced education and physiotherapy before knee replacement; is it worth it? A systematic review. *Physiotherapy* 2014;100(4):305-12. doi: 10.1016/j.physio.2014.03.003 [published Online First: 2014/06/03]
60. McDonald S, Page MJ, Beringer K, et al. Preoperative education for hip or knee replacement. *Cochrane Database Syst Rev* 2014(5):CD003526. doi: 10.1002/14651858.CD003526.pub3 [published Online First: 2014/05/14]
61. Santa Mina D, Clarke H, Ritvo P, et al. Effect of total-body prehabilitation on postoperative outcomes: a systematic review and meta-analysis. *Physiotherapy* 2014;100(3):196-207. doi: 10.1016/j.physio.2013.08.008 [published Online First: 2014/01/21]
62. Aydin D, Klit J, Jacobsen S, et al. No major effects of preoperative education in patients undergoing hip or knee replacement--a systematic review. *Dan Med J* 2015;62(7) [published Online First: 2015/07/18]
63. Cabilan CJ, Hines S, Munday J. The effectiveness of prehabilitation or preoperative exercise for surgical patients: a systematic review. *JBI Database System Rev Implement Rep* 2015;13(1):146-87. doi: 10.11124/jbisrir-2015-1885 [published Online First: 2015/10/09]
64. Kwok IH, Paton B, Haddad FS. Does Pre-Operative Physiotherapy Improve Outcomes in Primary Total Knee Arthroplasty? - A Systematic Review. *J Arthroplasty* 2015;30(9):1657-63. doi: 10.1016/j.arth.2015.04.013 [published Online First: 2015/04/29]
65. Wang L, Lee M, Zhang Z, et al. Does preoperative rehabilitation for patients planning to undergo joint replacement surgery improve outcomes? A systematic review and meta-analysis of randomised controlled trials. *BMJ Open* 2016;6(2):e009857. doi: 10.1136/bmjopen-2015-009857 [published Online First: 2016/02/04]
66. Chesham RA, Shanmugam S. Does preoperative physiotherapy improve postoperative, patient-based outcomes in older adults who have undergone total knee arthroplasty? A systematic review. *Physiother Theory Pract* 2017;33(1):9-30. doi: 10.1080/09593985.2016.1230660 [published Online First: 2016/10/14]
67. Peer MA, Rush R, Gallacher PD, et al. Pre-surgery exercise and post-operative physical function of people undergoing knee replacement surgery: A systematic review and meta-analysis of randomized controlled trials. *J Rehabil Med* 2017;49(4):304-15. doi: 10.2340/16501977-2210 [published Online First: 2017/03/30]
68. Zhang H, Hao X, Shuai T, et al. Efficacy of preoperative rehabilitation for total knee replacement: a systematic review. *Chinese journal of evidence-based medicine* 2017;17(9):1060-65. doi: 10.7507/1672-2531.201701093
69. Chen H, Li S, Ruan T, et al. Is it necessary to perform prehabilitation exercise for patients undergoing total knee arthroplasty: meta-analysis of randomized controlled trials. *Physician and Sportsmedicine* 2018;46(1):36-43. doi: http://dx.doi.org/10.1080/00913847.2018.1403274
70. Goldsmith LJ, Suryaprakash N, Randall E, et al. The importance of informational, clinical and personal support in patient experience with total knee replacement: a qualitative investigation. *BMC Musculoskelet Disord* 2017;18(1):127. doi: 10.1186/s12891-017-1474-8 [published Online First: 2017/03/28]

For peer review only - http://bmjopen.bmj.com/site/about/guidelines.xhtml

71. Edwards RR, Haythornthwaite JA, Smith MT, et al. Catastrophizing and depressive symptoms as prospective predictors of outcomes following total knee replacement. *Pain Res Manag* 2009;14(4):307-11.
72. Riddle DL, Keefe FJ, Ang DC, et al. Pain Coping Skills Training for Patients Who Catastrophize About Pain Prior to Knee Arthroplasty: A Multisite Randomized Clinical Trial. *J Bone Joint Surg Am* 2019;101(3):218-27. doi: 10.2106/JBJS.18.00621 [published Online First: 2019/02/08]

For peer review only

For peer review only - http://bmjopen.bmj.com/site/about/guidelines.xhtml

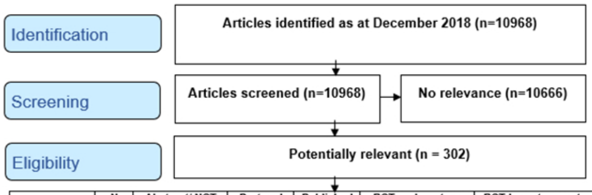

	N	Abstract/ NCT record only	Protocol only	Published RCT	RCT-no long term follow up	RCT Long term outcome but no pain score	Intervention had post-operative component	Eligible	Paper associated with included study	Review
Exercise/ education	203	8	13	76	68	0	11	8	14	49
Drug treatments	43	1	2	**12**	14	3	0	0	0	9
Weight loss	15	1	0	3	1	0	2	0	0	8
Electrical stimulation	5	2	0	**2**	2	0	0	0	0	1
Smoking cessation	13	0	0	**1**	0	1	1	0	3	4
Bacterial screening	8	3	0	**1**	0	1	0	0	0	2
Glucose testing	2	0	0	0	0	0	0	0	0	2
Supplements	13	4	2	6	3	0	3	0	0	1
Total	302	19	17	101	88	5	17	8	17	76

Figure 1

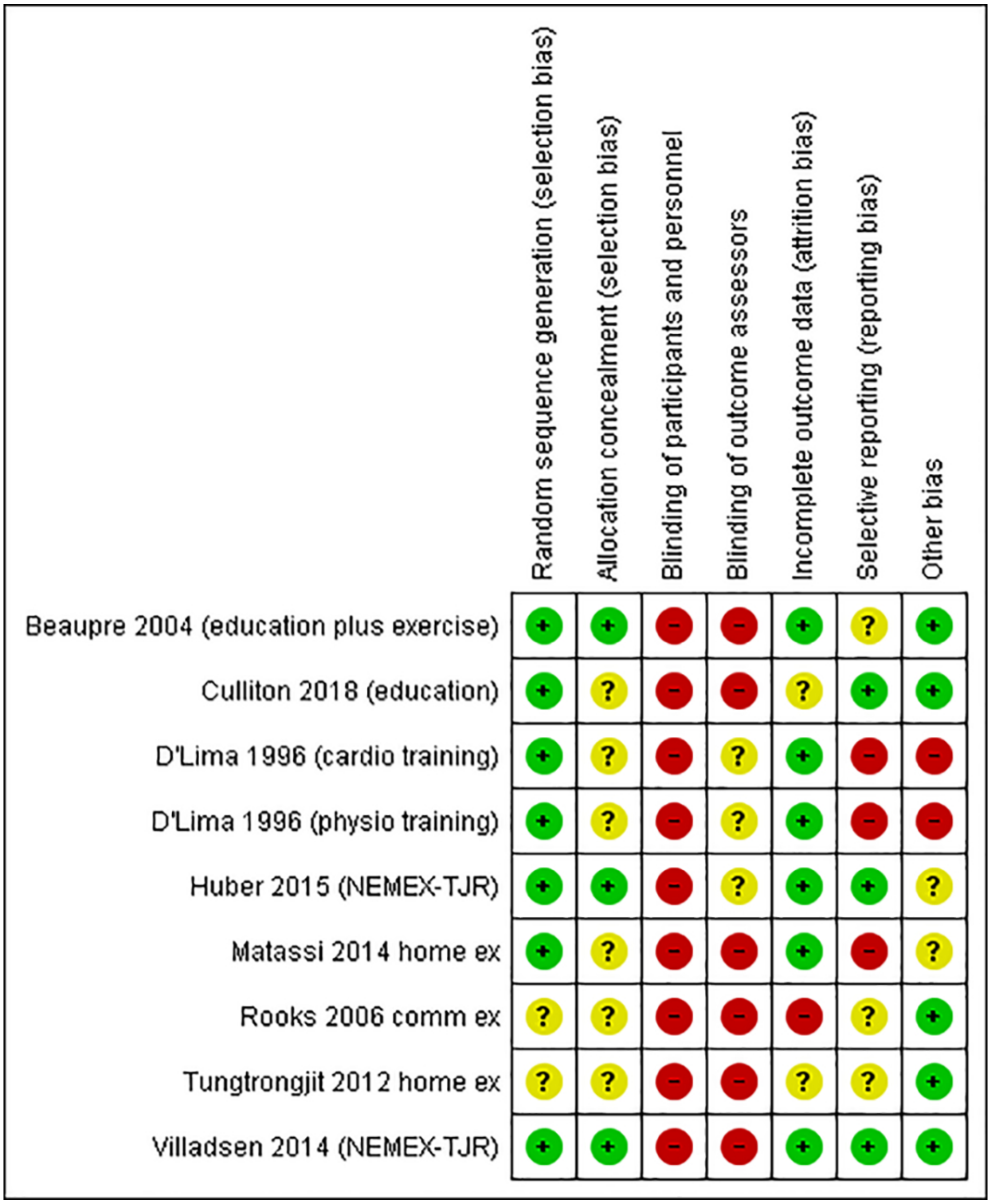
Random sequence generation (selection bias)
Allocation concealment (selection bias)
Blinding of participants and personnel
Blinding of outcome assessors
Incomplete outcome data (attrition bias)
Selective reporting (reporting bias)
Other bias
Beaupre 2004 (education plus exercise)
Culliton 2018 (education)
D'Lima 1996 (cardio training)
D'Lima 1996 (physio training)
Huber 2015 (NEMEX-TJR)
Matassi 2014 home ex
Rooks 2006 comm ex
Tungtrongjit 2012 home ex
Villadsen 2014 (NEMEX-TJR)

Figure 2

Study or Subgroup	Exercise Mean	Exercise SD	Exercise Total	Standard care Mean	Standard care SD	Standard care Total	Weight	Std. Mean Difference IV, Random, 95% CI
**1.1.1 Pain at 6 - 12 months (various scales)**								
D'Lima 1996 (cardio training)	28.7	16.02	10	27	16.02	5	6.0%	0.10 [-0.97, 1.17]
D'Lima 1996 (physio training)	29	16.02	10	27	16.03	5	6.0%	0.12 [-0.96, 1.19]
Rooks 2006 comm ex	-2.4	2.7	14	-2.3	2	15	13.0%	-0.04 [-0.77, 0.69]
Fernandes 2017 NEMEX-TJR	74.2	18.6	37	73.2	21.7	37	33.3%	0.05 [-0.41, 0.50]
Huber 2015 NEMEX-TJR	87.76	11.09	17	85.95	13.39	19	16.1%	0.14 [-0.51, 0.80]
Tungtrongjit 2012 home ex	-2.5	3.1	30	-4.8	4.2	30	25.7%	0.62 [0.10, 1.13]
**Subtotal (95% CI)**			**118**			**111**	**100.0%**	**0.20 [-0.06, 0.47]**

Heterogeneity: $Tau^2 = 0.00$; $Chi^2 = 3.39$, df = 5 (P = 0.64); $I^2 = 0\%$
Test for overall effect: Z = 1.53 (P = 0.13)

Test for subgroup differences: Not applicable

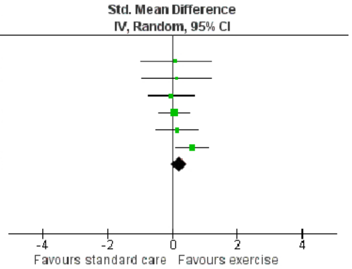

Figure 3

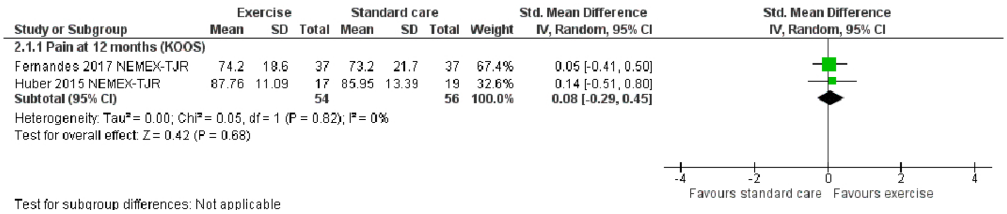
Exercise
Standard care
Std. Mean Difference
Std. Mean Difference
Study or Subgroup
Mean SD Total Mean SD Total Weight IV, Random, 95% CI
IV, Random, 95% CI
2.1.1 Pain at 12 months (KOOS)
Fernandes 2017 NEMEX-TJR 74.2 18.6 37 73.2 21.7 37 67.4% 0.05 [-0.41, 0.50]
Huber 2015 NEMEX-TJR 87.76 11.09 17 85.95 13.39 19 32.6% 0.14 [-0.51, 0.80]
Subtotal (95% CI) 54 56 100.0% 0.08 [-0.29, 0.45]
Heterogeneity: Tau² = 0.00; Chi² = 0.05, df = 1 (P = 0.82); I² = 0%
Test for overall effect: Z = 0.42 (P = 0.68)
-4
-2
0
2
4
Favours standard care
Favours exercise
Test for subgroup differences: Not applicable

Figure 4

	Exercise & education			Standard care				Std. Mean Difference
**Study or Subgroup**	**Mean**	**SD**	**Total**	**Mean**	**SD**	**Total**	**Weight**	**IV, Random, 95% CI**
**3.1.2 Pain at 12 months (WOMAC)**								
Beaupre 2004 (education plus exercise)	82	13	51	80	16	58	100.0%	0.14 [-0.24, 0.51]
**Subtotal (95% CI)**			**51**			**58**	**100.0%**	**0.14 [-0.24, 0.51]**

Heterogeneity: Not applicable
Test for overall effect: Z = 0.70 (P = 0.48)

Test for subgroup differences: Not applicable

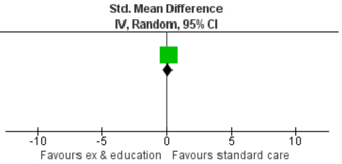

Figure 5

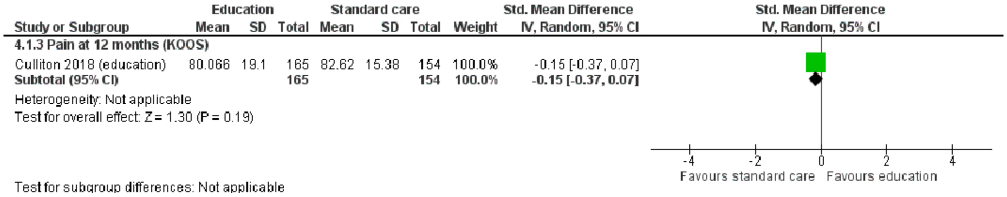
Education
Standard care
Std. Mean Difference
Std. Mean Difference
Study or Subgroup
Mean SD Total Mean SD Total Weight
IV, Random, 95% CI
IV, Random, 95% CI
4.1.3 Pain at 12 months (KOOS)
Culliton 2018 (education) 80.066 19.1 165 82.62 15.38 154 100.0% -0.15 [-0.37, 0.07]
Subtotal (95% CI) 165 154 100.0% -0.15 [-0.37, 0.07]
Heterogeneity: Not applicable
Test for overall effect: Z = 1.30 (P = 0.19)
-4
-2
0
2
4
Favours standard care
Favours education
Test for subgroup differences: Not applicable

Figure 6

Appendix 1. Search strategy as applied in MEDLINE (tailored as relevant to other databases)

1 randomized controlled trial/ or randomized controlled trial.pt.

2 controlled clinical trial.pt.

3 randomized.ab.

4 placebo.ab.

5 randomly.ab

6 trial.ab

7 randomised.tw

8 1 or 2 or 3 or 4 or 5 or 6 or 7

9 review/

10 'systematic review$'.mp

11 9 or 10

12 8 or 11

13 Arthroplasty, Replacement, Knee/

14 Knee Prosthesis/

15 (arthoplast$ adj3 knee$).mp. [mp=title, abstract, original title, name of substance word, subject heading word, keyword heading word, protocol supplementary concept word, rare disease supplementary concept word, unique identifier]

16 (knee$ adj3 replac$).mp. [mp=title, abstract, original title, name of substance word, subject heading word, keyword heading word, protocol supplementary concept word, rare disease supplementary concept word, unique identifier]

17 (knee adj3 implant$).mp. [mp=title, abstract, original title, name of substance word, subject heading word, keyword heading word, protocol supplementary concept word, rare disease supplementary concept word, unique identifier]

18 13 or 14 or 15 or 16 or 17

19 12 and 18

For peer review only

Reporting checklist for systematic review and meta-analysis.

Based on the PRISMA guidelines.

Instructions to authors

Complete this checklist by entering the page numbers from your manuscript where readers will find each of the items listed below.

Your article may not currently address all the items on the checklist. Please modify your text to include the missing information. If you are certain that an item does not apply, please write "n/a" and provide a short explanation.

Upload your completed checklist as an extra file when you submit to a journal.

In your methods section, say that you used the PRISMAreporting guidelines, and cite them as:

Moher D, Liberati A, Tetzlaff J, Altman DG, The PRISMA Group. Preferred Reporting Items for Systematic Reviews and Meta-Analyses: The PRISMA Statement

For peer review only

		Reporting Item	Page Number
**Title**			
	#1	Identify the report as a systematic review, meta-analysis, or both.	1
**Abstract**			

Structured summary	#2	Provide a structured summary including, as applicable: background; objectives; data sources; study eligibility criteria, participants, and interventions; study appraisal and synthesis methods; results; limitations; conclusions and implications of key findings; systematic review registration number	2
**Introduction**			
Rationale	#3	Describe the rationale for the review in the context of what is already known.	4
Objectives	#4	Provide an explicit statement of questions being addressed with reference to participants, interventions, comparisons, outcomes, and study design (PICOS).	5-6
**Methods**			
Protocol and registration	#5	Indicate if a review protocol exists, if and where it can be accessed (e.g., Web address) and, if available, provide registration information including the registration number.	1 and 5
Eligibility criteria	#6	Specify study characteristics (e.g., PICOS, length of follow-up) and report characteristics (e.g., years considered, language, publication status) used as criteria for eligibility, giving rational	5-6

For peer review only

Information sources	#7	Describe all information sources in the search (e.g., databases with dates of coverage, contact with study authors to identify additional studies) and date last searched.	1 and 6
Search	#8	Present full electronic search strategy for at least one database, including any limits used, such that it could be repeated.	Appendix 1
Study selection	#9	State the process for selecting studies (i.e., for screening, for determining eligibility, for inclusion in the systematic review, and, if applicable, for inclusion in the meta-analysis).	7
Data collection process	#10	Describe the method of data extraction from reports (e.g., piloted forms, independently by two reviewers) and any processes for obtaining and confirming data from investigators.	7
Data items	#11	List and define all variables for which data were sought (e.g., PICOS, funding sources), and any assumptions and simplifications made.	7
Risk of bias in individual studies	#12	Describe methods used for assessing risk of bias in individual studies (including specification of whether this was done at the study or outcome level, or both), and how this information is to be used in any data synthesis.	7
Summary measures	#13	State the principal summary measures (e.g., risk ratio, difference in means).	7-8

For peer review only

Planned methods of analyis	#14	Describe the methods of handling data and combining results of studies, if done, including measures of consistency (e.g., I2) for each meta-analysis.	7-8
Risk of bias across studies	#15	Specify any assessment of risk of bias that may affect the cumulative evidence (e.g., publication bias, selective reporting within studies).	7
Additional analyses	#16	Describe methods of additional analyses (e.g., sensitivity or subgroup analyses, meta-regression), if done, indicating which were pre-specified.	7-8
**Results**			
Study selection	#17	Give numbers of studies screened, assessed for eligibility, and included in the review, with reasons for exclusions at each stage, ideally with a flow diagram.	8 and Figure 1
Study characteristics	#18	For each study, present characteristics for which data were extracted (e.g., study size, PICOS, follow-up period) and provide the citation.	17-19
Risk of bias within studies	#19	Present data on risk of bias of each study and, if available, any outcome-level assessment (see Item 12).	Figure 2 & Table 2
Results of individual studies	#20	For all outcomes considered (benefits and harms), present, for each study: (a) simple summary data for each intervention group and (b) effect estimates and confidence intervals, ideally with a forest plot.	Figures 3 to 6

For peer review only

Synthesis of results	#21	Present the main results of the review. If meta-analyses are done, include for each, confidence intervals and measures of consistency.	11-12
Risk of bias across studies	#22	Present results of any assessment of risk of bias across studies (see Item 15).	12 and Figure 2
Additional analysis	#23	Give results of additional analyses, if done (e.g., sensitivity or subgroup analyses, meta-regression [see Item 16]).	Figure 4
**Discussion**			
Summary of Evidence	#24	Summarize the main findings, including the strength of evidence for each main outcome; consider their relevance to key groups (e.g., health care providers, users, and policy makers	12-13
Limitations	#25	Discuss limitations at study and outcome level (e.g., risk of bias), and at review level (e.g., incomplete retrieval of identified research, reporting bias).	13
Conclusions	#26	Provide a general interpretation of the results in the context of other evidence, and implications for future research.	13-14
**Funding**			
Funding	#27	Describe sources of funding or other support (e.g., supply of data) for the systematic review; role of funders for the systematic review.	16

Notes:

For peer review only

- Appendix 1
- 17: 8 and Figure 1
- 20: Figures 3 to 6
- 22: 11 and Figure 2 The PRISMA checklist is distributed under the terms of the Creative Commons Attribution License CC-BY. This checklist was completed on 26. July 2019 using https://www.goodreports.org/, a tool made by the EQUATOR Network in collaboration with Penelope.ai

For peer review only

BMJ Open

The effects of pre-surgical interventions on chronic pain after total knee replacement: A systematic review and meta-analysis of randomised controlled trials

Journal:	*BMJ Open*
Manuscript ID	bmjopen-2019-033248.R1
Article Type:	Original research
Date Submitted by the Author:	30-Oct-2019
Complete List of Authors:	Dennis, Jane; University of Bristol, Musculoskeletal Research Unit<br>Wylde, Vikki; University of Bristol, Musculoskeletal Research Unit<br>Gooberman-Hill, Rachael; University of Bristol, Bristol Medical School<br>Blom, AW; University of Bristol,<br>Beswick, Andrew; University of Bristol, Musculoskeletal Research Unit
**Primary Subject Heading**:	Surgery
Secondary Subject Heading:	Rehabilitation medicine
Keywords:	chronic post-surgical pain, prevention, systematic review, total knee replacement

SCHOLARONE™
Manuscripts

The effects of pre-surgical interventions on chronic pain after total knee replacement: A systematic review and meta-analysis of randomised controlled trials

Jane Dennis[1]

Vikki Wylde[1,2]

Rachael Gooberman-Hill[1,2]

Ashley W Blom[1,2]

Andrew D Beswick[1]

[1] Musculoskeletal Research Unit, Translational Health Sciences, Bristol Medical School, University of Bristol

[2] National Institute for Health Research Bristol Biomedical Research Centre, University Hospitals Bristol NHS Foundation Trust and University of Bristol

jane.dennis@bristol.ac.uk,v.wylde@bristol.ac.uk,r.gooberman-hill@bristol.ac.uk,ashley.blom@bristol.ac.uk,andy.beswick@bristol.ac.uk

Corresponding author: Jane Dennis

Word count: 5003

Musculoskeletal Research Unit
Translational Health Sciences
Bristol Medical School
University of Bristol
Learning and Research Building, Level 1
Southmead Hospital
Bristol
BS10 5NB

Keywords: chronic post-surgical pain; prevention; systematic review; total knee replacement

For peer review only - http://bmjopen.bmj.com/site/about/guidelines.xhtml

Abstract

Objective

Nearly 100,000 primary total knee replacements [TKR] are performed in the UK annually. The primary aim of TKR is pain relief, but 10-34% of patients report chronic pain. This aim of this systematic review was to evaluate the effectiveness of pre-surgical interventions in preventing chronic pain after TKR.

Design

MEDLINE, Embase, CINAHL, *The Cochrane Library* and PsycINFO were searched from inception to December 2018. Screening and data extraction were performed by two authors. Meta-analysis was conducted using a random effects model. Risk of bias was assessed using the Cochrane tool and quality of evidence assessed by GRADE

Primary and secondary outcomes

Pain at six months or longer; adverse events.

Interventions

Presurgical interventions aimed at improving TKR outcomes.

Results

Eight RCTs with data from 960 participants were included. The studies involved nine eligible comparisons. We found moderate quality evidence of no effect of exercise programmes on chronic pain after TKR, based on a meta-analysis of 5 studies with 229 participants (SMD 0.20, 95% CI -0.06 to 0.47, $I^2 = 0\%$). Sensitivity analysis restricted to studies at overall low risk of bias confirmed findings. Studies evaluating exercise (n=1), combined exercise and education intervention (n=1) and education alone (n=1) suggested similar findings. Adverse event data were reported by most studies, but events were too few to draw conclusions.

Conclusions

We found low to moderate quality evidence to suggest that neither pre-operative exercise, education nor a combination of both is effective in preventing chronic pain after TKR. This review also identified a lack of evaluation of other pre-operative interventions, such as multi-modal pain management, which may improve long-term pain outcomes after TKR.

PROSPERO REGISTRATION NUMBER: CRD42017041382.

For peer review only - http://bmjopen.bmj.com/site/about/guidelines.xhtml

Strengths and limitations of this study

- This systematic review evaluates recent evidence of the effectiveness of presurgical interventions in preventing chronic pain after total knee replacement
- Synthesis of adverse events data was not possible
- We only included studies that completed intervention delivery during the pre-operative period and did not include studies that evaluated interventions that began in the pre-operative period but extended into the peri-operative and post-operative period

For peer review only

For peer review only - http://bmjopen.bmj.com/site/about/guidelines.xhtml

Background

Osteoarthritis is the most common condition that affects the knee joint and causes considerable pain and disability. Effective treatments noted in guidelines include pain medications, exercise, and weight loss when appropriate[1, 2]. If symptoms do not respond to pharmacological and conservative treatments, people may receive a total knee replacement (TKR). Annually in the UK, nearly 100,000 primary TKRs are performed[3, 4] and in the USA in 2010, about five million people were estimated to be living with a TKR[5]. Outcomes are good for many, but a systematic review found that 10-34% of patients report unfavourable long-term pain outcomes at between three months and five years after TKR[6] which is associated with dissatisfaction with the outcome of surgery[7, 8].

Some patient factors are associated with poor long-term pain outcomes. Using structural equation modelling, Sayers and colleagues demonstrated that pain during the pre-surgical period, particularly on movement, is strongly associated with chronic post-surgical pain, and a potentially important target for intervention[9]. Other pre-surgical risk factors for pain identified in multivariable analyses in representative populations include: poorer physical function, body mass index ≥35 kg/m^2 and poor general mental health[10]. With knowledge of risk factors for chronic pain, there is the potential for targeting care to those at high risk, or for risk factor modification[10, 11]. While many pre-surgical interventions focus on preparing the patient for the procedure and hospital stay, reducing peri-operative pain and facilitating early mobilisation and recovery[10], their impact on the key outcome of chronic pain remains to be established. This is achievable through high quality, adequately powered randomised controlled trials with long-term follow up for patient related pain outcomes and ultimately through well-conducted systematic reviews with thorough consideration of sources of bias that may influence study results.

Numerous systematic reviews have been reported in this area. Twelve of these had searches conducted more than five years ago[11-22], and five exclusively reported short-term outcomes (three months or less after surgery)[23-27]. Two reviews considered the outcome of long-term pain[28, 29]. Chesham and Shanmugam identified two randomised controlled trials in their search window (2004-2014) that reported no difference in pain at six months after surgery in patients randomised to exercise or control[28]. Wang and colleagues identified three randomised controlled trials published before November 2015 that reported long-term pain[29]. They noted a small difference in pain at four weeks after surgery but this finding was not durable in the longer term.

In this systematic review and meta-analysis, we assessed the effectiveness of pre-operative interventions in preventing chronic pain in patients receiving TKR. Previous reviews have

For peer review only - http://bmjopen.bmj.com/site/about/guidelines.xhtml

largely focused on specific interventions but we have opted for a temporal framework and sought to include any intervention conducted before surgery. This review is part of a broad suite of reviews considering the timing of intervention throughout the TKR care trajectory[30, 31].

Methods

The protocol for this systematic review was registered with PROSPERO (CRD42017041382)[32]. We formulated the research question according to the PICO principle[33] and used methods based on those recommended by Cochrane[34]. Reporting was in accordance with Preferred Reporting Items for Systematic Reviews and Meta-Analyses (PRISMA) guidelines[35] and a checklist is included in the Appendix.

Patient and public involvement (PPI)

Before starting the review, we held a meeting with stakeholders and patient representatives and discussed inclusion criteria and outcomes. Areas of potential relevance for pre-surgical intervention were highlighted, specifically exercise, education, psychological therapies, weight management, nutritional supplements, management of co-morbidities, and pain management with intra-articular injections. The importance of patient-reported pain as an outcome after TKR was emphasised. A musculoskeletal PPI forum has discussed the review and will advise on plain language summaries and dissemination[36].

Types of studies

To limit selection bias, we included only randomised controlled trials. Studies reported only as abstracts or unobtainable as full text copies were excluded. Language was not an exclusion criterion.

Participants

Eligible participants were adults on the waiting lists for total knee replacement, up until the point of admission for surgery.

Interventions

Preventive interventions (pharmacological or non-pharmacological) delivered during the pre-surgical period were included. Studies where delivery of the intervention extended into the postoperative period were excluded.

Comparator group

Comparator group participants included those who had received usual care or an alternative intervention.

For peer review only - http://bmjopen.bmj.com/site/about/guidelines.xhtml

For peer review only

Outcomes

In preference, we considered patient-reported joint-specific pain intensity, typically measured using pain domains of the Oxford Knee Score (OKS)[37], Western Ontario and McMaster Universities Osteoarthritis Index (WOMAC)[38] or Knee injury and Osteoarthritis Outcome Score (KOOS)[39] at six months or longer after knee replacement. We selected these outcomes in preference to pain components of Knee Society's Clinical and Functional Scoring System (KSS)[40] and Hospital for Special Surgery Knee Score (HSS)[41] in which pain assessment is limited to one or two questions on intensity[42] and relies on clinician assessment which may not reflect the views of patients[43]. The choice of outcome timing reflects the time when, for many, pain levels have optimised[44]. If joint specific measures were not available, then we planned to use pain dimensions from quality of life measures including Short Form 36 Health Survey (SF-36)[45] and Short Form 12 Health Survey (SF-12)[46]. Had neither joint-specific nor generic pain outcomes been available, we planned to use data for pain measured using a visual analogue scale (VAS) or numerical rating scale (NRS). When no patient-reported outcome measure was reported, we used pain data from surgeon-assessed scores. When discrete pain data were unavailable, we report overall data that included those relating to pain, typically in combination with function and other factors.

Where data were available, we planned to report proportions of patients with a favourable pain outcome (for example "no worse than mild pain") as recommended in clinical trials[47] and systematic reviews[48].

Data on all adverse events were extracted and summarised narratively. Our focus was on adverse events related to the intervention, although we also report those related to surgery.

Study design

Randomised controlled trials of interventions commencing and completing within the pre-surgical period, with follow up at six months or longer after surgery for total knee replacement, were identified and assessed for eligibility. Authors reporting long-term outcomes of any type (typically, function only) were contacted to check whether pain outcome data were also available; those reporting aggregated data for hip replacements together with knee replacements were also contacted. Studies meeting these criteria and reporting pain data or composite scores including pain were included.

Database searches

We established a database of all randomised controlled trials in knee replacement, in preparation for a suite of systematic reviews in chronic pain after TKR[30, 31]. Relevant trials were identified through searches (last updated on 18th December 2018) in: MEDLINE,

For peer review only - http://bmjopen.bmj.com/site/about/guidelines.xhtml

Embase and PsycINFO on OvidSP; CINAHL on EBSCOhost; and *The Cochrane Library*. The search strategy as applied in MEDLINE is shown in Appendix 1. Within this resource, we searched for interventions conducted in the pre-surgical setting.

Citations and reference lists of key reviews and randomised trials were checked in ISI Web of Science. The ISRTN registry on BioMed Central was searched for ongoing randomised trials which were then checked for subsequent publication. No language restrictions were applied, and data from potentially relevant non-English language articles were extracted and translated to establish eligibility. Studies reported only as abstracts or that we were unable to acquire as full text copies using inter-library loans or author contact were excluded.

Study screening and data extraction

Records identified by searches were imported into Endnote X9 (Thomson Reuters). An initial screen for potential eligibility was undertaken by one review author (JD) with articles excluded only if clearly irrelevant. Typically, these were studies in osteoarthritis with no surgery, arthroscopy only, or surgery involving joints other than the knee. Subsequently, abstracts and full text articles were screened independently by two review authors. After consensus, reasons for exclusion were recorded.

Data from relevant randomised trials were extracted by one review author onto forms which were tested and refined before use. Microsoft Excel was used to record abbreviated information, specifically on: country where study was conducted; dates of recruitment; participant characteristics (indication, age, sex); inclusion and exclusion criteria; intervention and control content; setting, timing, duration and intensity of intervention; follow up data collection (including loss to follow up); instruments used to collect data on pain; outcome data including pain and adverse events. Data extracted were checked against source material by a second review author.

Group means and standard deviations of pain outcome measures were recorded at the longest follow up interval. Data with which to calculate proportions of patients with a poor outcome, however defined, were sought. Authors were contacted for unpublished data. Details of adverse events of any type were extracted.

Risk of bias within included studies

Potential sources of bias were assessed using the Cochrane risk of bias tool[49] by two review authors (JD and ADB) working independently; disagreements were adjudicated by a third (VW). We present analyses where possible showing data from all relevant studies for a given outcome, but also conducted sensitivity analyses excluding studies assessed to be at overall high risk of bias.

For peer review only - http://bmjopen.bmj.com/site/about/guidelines.xhtml

Data analysis

When studies reported pain outcome data, these were presented and pooled where possible, using Review Manager 5.0[50]. As the control group in one study was shared by two active interventions, the sample size in meta-analysis was halved for the control group, to preserve independence of findings[34]. Results are presented for one comparison in the form of standardised mean differences (SMD), as instruments used to measure outcomes varied.

A random effects model was planned, due to anticipated heterogeneity as described below. A sensitivity analysis excluding those comparisons for which we had imputed data and/or studies we had assessed as being at overall high risk of bias was conducted. At protocol stage[32], we planned to generate funnel plots, if ten or more studies with similar interventions and controls were identified, as a method of estimating the risk of publication bias. In the event, data were not sufficient to do so.

Heterogeneity between studies (considering both magnitude and direction of effect) was assessed using visual inspection of the graph and the I^2-statistic. We anticipated sources of heterogeneity justifying subgroup analyses might include format and intensity of intervention; however, statistical heterogeneity proved to be unimportant and data were insufficient to conduct such analyses.

GRADE approach to evidence

We used the Grading of Recommendations Assessment, Development and Evaluation (GRADE) approach to assess the quality of the evidence[51], specifically study limitations, consistency of effect, imprecision, indirectness and publication bias.

Results

Conduct of the review is summarised in Figure 1. Searches identified 10,968 articles. Of these, 302 potentially relevant reports were considered in detail, of which 100 were identified as RCTs (some with multiple publications). Eighty-eight did not follow up participants to a minimum of six months. Four trials involving different interventions (smoking cessation[52], testosterone injections[53], epoetin-α injections[54], and bacterial screening[55] followed by decolonisation) did meet eligibility criteria for long term follow-up, but none of these included outcome data related to pain.

After detailed evaluation, ten articles reporting data from eight RCTs[56-63] were included in the review. These ten RCTs included data from 960 participants randomised to nine eligible comparisons. The interventions evaluated in the included studies were exercise, education, or a combination of both.

For peer review only - http://bmjopen.bmj.com/site/about/guidelines.xhtml

Seven publications assessed the impact of a single intervention (exercise (5), education (1), or a combination of both exercise and education (1)) against a no-treatment or attentional control. One publication reported relative effects of two different physical interventions (cardio-based exercise or physiotherapy) against a no treatment control[56]. We analysed data from the latter publication as two separate studies, dividing the control group during analysis so as to preserve independence of findings. Details of studies, including intervention characteristics, are shown in Table 1; risk of bias assessments in Figure 2; meta-analysis and sensitivity analyses in Figures 3 to 6. Finally, GRADE assessments of the quality of evidence for these classes of intervention for the outcomes of pain and serious adverse events are shown in Table 2.

Overview of study settings, sample sizes and interventions

Included studies were published between 1996 and 2018. Two studies were conducted in Canada[57, 63], two in the USA[56, 62], and one each in Belgium[60], Denmark[58], Switzerland[59] and Thailand[61]. Exercise interventions were delivered within clinics or within the home; education interventions were delivered within clinics in the studies in which it formed part of a composite intervention, or online when it was assessed in isolation.

Samples at recruitment ranged from 30 to 416 participants. The total number of participants involved in studies of exercise alone was 373; exercise and education together, 131; and education alone, 416.

Sample size calculations were reported by most investigators - but only one was based on the primary outcome used within this review (pain at six months). Other studies had as their main focus: satisfaction with TKR[63]; a short-term reduction of 10 points on the HSS and the reduction of hospital stay by one day[56]; the Chair Stand test[59]; a difference of 10 degrees in passive flexion[60]; an 8 point difference in WOMAC[62]; and a 10 point difference on the KOOS ADL subscale[58]. The variability in foci amongst studies makes it difficult for us to estimate a reasonable optimal information size for the review, particularly as some investigators revised their sample size estimates upwards as part of considering limitations of their studies[56, 57].

Participants

Five studies exclusively recruited participants undergoing TKR for osteoarthritis[57, 59-61, 63]; one permitted those with rheumatoid arthritis[56]; and two included participants undergoing either hip or knee replacement for osteoarthritis, with long-term data reported separately[62] or in aggregate[58]. Mean age of participants ranged from 63 to 70 across studies. Gender, where reported, varied greatly, with studies recruiting 17%[61] to 60% women[56].

Interventions

For peer review only - http://bmjopen.bmj.com/site/about/guidelines.xhtml

Seven interventions assessed within six studies[56, 58-62] were exercise-based; one was multifactorial with exercise and an education component focusing on walking with crutches, bed mobility and postoperative range of movement[57]; and one featured an online educational component alone[63].

All exercise interventions included strengthening[56-62], with additional components targeting gait re-education[57], functional exercise[57, 59], range of movement[56, 57, 59] cardiovascular conditioning[56, 59, 62] stretching[56] and flexibility[59, 60, 62]. Two studies evaluated a particular manualised programme, 'NEuroMuscular Exercise training program for patients with knee or hip osteoarthritis assigned for total joint replacement' (NEMEX-TJR)[64] which is described as a series of neuromuscular exercises combined with biomechanical training. One study included a series of pool-based exercises as well as standard indoor exercises[62]. The majority of exercise-based interventions required participants to travel to clinics or community-based exercise centres but two[60, 61] involved exclusively home-based exercise. Staff delivering interventions were, in the majority of cases, physiotherapists; exceptions were one trial in which the intervention was delivered by an exercise physiologist[56] and one in which it was unclear who delivered the intervention[60].

Participants in the study evaluating both exercise and education received a single session of education (reinforced by provision of a written materials) concerning crutch walking on level ground and stairs, bed mobility and transfers, and post-operative range of motion routines[57]. Those in the study focussing solely on education[63] received a 31 page guide as well as access to an online learning tool during their preadmission consultation, followed by subsequent reminders of their access. The customised e-learning tool included 32 brief (1-2 minute) professionally filmed educational videos presented by either surgeons, therapists, or previous recipients of TKRs, in which expectations related to pain, functional outcomes, limitations, and restrictions were addressed, as well as animations allowing them to visualise the surgery itself.

Intensity, duration, measures of adherence / compliance

Exercise interventions were delivered for between 3 and 12 weeks, 2-7 times a week. The shortest duration of intervention was a three-week home-based intervention[61] which featured the most intense dose (3 sessions per day, 7 days a week). The longest period of intervention (8-12 weeks) took place in the two trials[58, 59] delivering the clinic-based NEMEX-TJR intervention[64]. Here, variability in duration could be determined by how close to the surgery a patient was at time of recruitment.

With regard to exercise interventions conducted within clinics or at home, investigators typically assessed adherence via logbooks[57-60] recording attendance and sometimes details

For peer review only - http://bmjopen.bmj.com/site/about/guidelines.xhtml

of achievement and effort. One study of a home-based intervention without formal reporting of adherence featured daily telephone calls to participants[61]; two clinic-based interventions[56] reported that all participants completed all sessions. The study focussing solely on education[63] assessed the number of overall 'hits' to different educational videos concerning different aspects of the TKR pathway.

Comparator groups

Amongst the trials assessing the effects of exercise or exercise combined with education, detail and nature of comparator groups varied. Five trials described comparator groups as 'usual care'. For three trials the descriptions of usual care were brief but indicated that participants continued with regular activities until surgery[57, 60, 61]. In one trial of cardiovascular exercise, usual care was described as participants having a single meeting with a physiotherapist before their operation for 45 minutes and being provided with printed material about post-operative exercise regimens[56]. In another trial, usual care involved all participants being offered a "standard preoperative educational package" comprising written information on the operation, 'expected postoperative progression', and recommended exercises, and a 3-hour in-clinic information session led by health professionals one week prior to surgery[58].

In the two other studies assessing exercise, the comparator groups were designed as attentional controls. In one, participants were sent information on joint replacement, recommended home modifications, and on preparing for surgery, reinforced by three telephone calls[62]. The most intensive control condition was 'knee school' offered alongside an exercise intervention but also offered to the control group participants[59]. The knee school included formal teaching and peer discussions, was led by an experienced and specially-trained physiotherapist over three weekly individual or group sessions. Participants were provided with information on anatomy (including photos, diagrams and models), recommended activities after surgery, post-operative pain management, and rehabilitation.

In the trial evaluating online education, the control group were provided with a 31 page copy of 'My Guide to Total Knee Joint Replacement'[63].

Outcomes and outcome data

Six studies reported a joint- or condition- specific patient reported pain outcome measure[57-59, 61-63]. In two publications (one reporting data for two separate comparisons), the outcomes reported were surgeon-assessed[56, 60].

For peer review only - http://bmjopen.bmj.com/site/about/guidelines.xhtml

The latest pain assessment was reported at about six months[56, 57, 59, 60] or 12 months after surgery[61-63]. Measures used were WOMAC pain[57, 61], KOOS pain[59, 63], SF-36 bodily pain[57, 59, 63], VAS pain[61], HSS pain score[56], and KSS[60].

We made robust attempts to obtain data through contact with investigators from studies in which data were provided within graphs without standard deviations[56, 59], in tables with means but without standard deviations[63], as 'non significant' only[60], or as part of aggregated hip and knee data[58]. In three studies, investigators generously provided relevant data in the form of group means and SDs and these data were used[58, 59, 63]. Following information that data for one study had been permanently lost[56], we measured WOMAC pain data from a graph and, in the absence of standard deviations, used values from a cohort of patients with the same outcome measure reported after total knee replacement but converted from a 50 point to a 30 point scale[65]. The first author of an exercise-based study which had reported pain findings as simply 'n.s.' (non-significant), responded to our enquiry to indicate that they could no longer access study data.

Risk of bias

Four studies were assessed as being at an overall low risk of bias[57, 59, 60]. In other studies, we had concerns about potential biases due to differences in baseline patient characteristics in two comparisons[56] and in one study each, large losses to follow up[62], and issues regarding sequence generation[61].

Effects of intervention: exercise alone

<u>Pain at six months or longer (Figure 3)</u>

Combined results of six eligible interventions reported in 5 studies [56, 58, 59, 61, 63] indicate no clear effect of intervention on long-term pain (SMD 0.20, 95% CI -0.06 to 0.47; participants = 229). There was no heterogeneity across studies, $Chi^2 = 3.39$, $I^2 = 0\%$.

In a sensitivity analysis restricted to two studies with low risk of bias[57, 59], group results provided even less suggestion of difference between groups (SMD 0.08, 95% CI -0.29 to 0.45; participants = 110; $I^2 = 0\%$) (see Figure 4).

Results for one study (n = 122) within this category for which no data were suitable for meta-analysis are in line with the results above, with 'non significant'[60] findings.

Effects of intervention: exercise combined with education

<u>Pain at 6 months or longer (Figure 5)</u>

Results of a single study of exercise with the additional component of one session of education (n = 109 followed up) suggest no clear difference between intervention and

For peer review only - http://bmjopen.bmj.com/site/about/guidelines.xhtml

standard care on long-term pain as assessed by the WOMAC pain scale (MD 2.00, 95% CI -3.45 to 7.45)[57].

Effects of intervention: education alone

Pain at 6 months or longer (Figure 6)

Results of a single study of education provided by an e-learning tool (n = 319) suggest no clear difference between intervention and standard care in terms of long-term pain as assessed on the KOOS (MD -2.55, 95% CI -6.35 to 1.24)[63].

Adverse events

Peri and post-surgical complications including infections were assessed in most studies; see Table 1. Where reported, these do not appear to have differed between groups. Two studies reported pre-surgical events we judged likely to be intervention-related[59, 60]; in each case, participants in an exercise intervention group reported increased pain. No data for this outcome were reported in the study focussing on education alone[63].

Discussion

Summary of main findings

In people with osteoarthritis receiving TKR, the potential value of interventions to manage pre-operative risk factors or to target care has frequently been assessed, most often focussing on interventions this review has identified (exercise or 'prehabilitation' and/or education with the aim of managing expectations). We found low to moderate quality evidence for no effect on our primary outcome (pain assessed at six months or longer) of either exercise, education or education combined with exercise, and we identified no eligible studies assessing the effects of any other eligible pre-operative interventions (e.g. weight loss programmes or smoking cessation).

Strengths and limitations of this study

A limitation of our review concerns our focus on prevention of long-term pain only. This decision was based on guidelines which emphasise that osteoarthritic pain which is not controlled by conservative treatments is a primary reason that people undergo TKR[66]. Prevention of long-term pain is a key determinant of patient satisfaction with TKR[67]. Although the primary outcome of many studies we identified was function, pain severity was an important secondary outcome. We cannot draw conclusions on the value of the interventions we have examined from the point of view of (for example) the benefit to individuals or society of quicker short term recovery, as proposed by some triallists[58].

For peer review only - http://bmjopen.bmj.com/site/about/guidelines.xhtml

For peer review only

The secondary outcome of this review was adverse events, but reporting was lacking. The poor quality of adverse event reporting in surgical trials is recognised[68], and standardisation is required to improve quality and reduce heterogeneity, particularly in orthopaedics. Although patient reported outcomes are frequently considered to be at high risk of bias, we considered them to be appropriate in this review where questionnaires were completed over six months after a presurgical intervention.

We also excluded studies in which the intervention continued beyond the period of the TKR procedure; a separate review considering studies including interventions which combined prehabilitation and post-TKR rehabilitation may well be justified.

This review in context

This is the first review in nearly a decade[14] which takes as its focus the preoperative period itself, rather than aiming at a class of interventions; the evidence base, however, remains limited.

Numerous reviews have appeared focusing on outcomes relating to pre-operative function and pain, the in-hospital experience, and long-term recovery, specifically following pre-operative exercise and/or education programmes involving joint replacement[12, 13, 15-29, 69]. Findings vary based on the outcomes and timeframes prioritised by review authors. In general, those concentrating on short term function and/or outcomes related to discharge report more positive findings as regards implications for both practice and future research[26]. Those considering the longer term are less sanguine. One review which evaluated the effects of ‘prehabilitation’ across *all* surgical patients concluded that “*prehabilitation studies are not recommended in patients with osteoarthritis for whom arthroplasty is planned*”[22]. Subsequently, amongst the most comprehensive of systematic reviews conducted in arthroplasty alone is that by Wang and colleagues[29]. They concluded that the effects of prehabilitation on long-term and indeed many short-term outcomes were too small to be considered clinically important.

Our review adds to the existing evidence base as it includes two trials not included in others[59, 63]. With broad inclusion criteria we aimed to identify diverse interventions conducted in the pre-operative period. Important issues that our review benefits from are preregistration of the study protocol to limit reporting bias, conduct of study according to PRISMA guidelines and comprehensive up to date searches of a range of appropriate literature databases. Experienced systematic reviewers undertook screening of articles, data extraction and risk of bias assessment in duplicate. Author contact was extensive and with the help of study investigators, we included data from studies which had been excluded from previous meta-

For peer review only - http://bmjopen.bmj.com/site/about/guidelines.xhtml

analyses or had been included with estimates for variances. Furthermore, use of GRADE methodology allowed us to assess the overall quality of the evidence.

Implications for practice and research

There are several possible explanations for the observed lack of a convincing effect of pre-operative interventions on long-term pain after TKR reported within this review. All pre-operative interventions conducted within an undifferentiated general population may appear ineffective in the long-term, as for many patients, TKR surgery is highly effective in reducing long-term pain. To detect an effect of intervention for the small but important minority of patients for whom TKR is not highly effective in reducing long-term pain, studies may need either to be much larger, or more focussed on particular patient subgroups. Despite the fact that patients report that appropriate and realistic support before surgery can help them achieve a long-term positive experience of surgery[70], it is acknowledged that it is difficult to recruit patients to randomised controlled trials before surgery and participants in existing trials may have been highly selected. Patients may have exhausted conservative strategies before surgery and, in evaluating new approaches, there may be an unwillingness among patients to participate in randomised trials[10]. Intervention content may have focused on improving pre-operative physical function and preparing patients for their hospital admission and recovery without specific reference or ‘flags’ to help identify participants whose recovery did not follow the norm. Interventions that we included may have focused on aspects of care that do not have a sustained effect on pain. For example, it could be argued that the only outcome necessary in an evaluation of bacterial screening and targeted treatment is the incidence of surgical site infections; however, superficial and particularly deep infections after knee replacement are associated with high levels of morbidity including pain.

An aspect of pre-surgical care with a possible link with chronic pain is lack of psychological support. As an example, pain catastrophizing has been implicated as a risk factor for chronic pain[71]. However, a recent trial where intervention began in the pre-surgical period and extended well beyond it found that pain coping skills training for people with high pain catastrophizing did not improve WOMAC pain scores at 12 months after TKR[72]. For other pre-operative strategies, such as smoking cessation, weight loss and comorbidity management, there may be tenuous, if any, mechanisms to link with long-term pain outcomes. However, they share a common aim in preparing patients for surgery and preventing adverse events that may limit the potential benefit of knee replacement, so we believe it right to advocate that studies investigating their effects incorporate long term follow-up.

Conclusion

For peer review only - http://bmjopen.bmj.com/site/about/guidelines.xhtml

Pre-surgical interventions have been evaluated in patients receiving TKR. We found low to moderate quality evidence to suggest that neither exercise, education nor a combination of both is effective in preventing long-term pain in adults scheduled to receive total knee replacement and it is difficult to recommend further research except of novel approaches and/or in specific populations. However, studies of pre-operative interventions with long-term follow up after surgery are feasible in other areas and for them, adequately powered, randomised controlled trials, planned and reported according to CONSORT standards, should be conducted to identify clinically effective pre-operative treatments to help patients achieve a long-term pain-free outcome after knee replacement.

Acknowledgements

We gratefully acknowledge unpublished outcome data from the following investigators: Dr Sharon Culliton, Dr Erika Huber and Dr Allan Villadsen. We also are thankful for confirmation concerning other study data from Professor Darryl D'Lima and Dr Fabrizio Matassi. We are also thankful for translation of Yunfei Li, University of Bristol, for his help in translating a paper from Chinese.

Author contributions

All authors contributed to the concept and design of the study. JD, ADB and VW contributed to the acquisition and analysis of data. JD and ADB drafted the article and VW, RG-H and AWB revised it critically for important intellectual content. JD and ADB take responsibility for the integrity of the work as a whole, from inception to finished article.

Funding

This study is funded by the National Institute for Health Research (NIHR) [NIHR Programme Grant for Applied Research (Grant Reference Number RP-PG-0613-20001)]. This study was also supported by the NIHR Biomedical Research Centre at the University Hospitals Bristol NHS Foundation Trust and the University of Bristol. The views expressed in this publication are those of the authors and not necessarily those of the NHS, the National Institute for Health Research or the Department of Health and Social Care.

Disclaimer

The views expressed are those of the authors and not necessarily those of the NHS, the NIHR or the Department of Health. The funder had no involvement in the study design, data collection, data analysis, interpretation of data or writing of the manuscript.

Competing interests

For peer review only - http://bmjopen.bmj.com/site/about/guidelines.xhtml

No author has any direct conflict of interest with the published work above. Outside of this work, VW and AWB are co-applicants on an institutional grant from Stryker for a study evaluating the outcomes of the Triathlon knee replacement.

Patient consent

Not required.

Data sharing statement

No additional data available.

Legend

Figure 1. PRISMA_Flow_Diagram

Figure 2. Risk of bias assessments

Figure 3. Forest plot: exercise

Figure 4. Forest plot: exercise sensitivity analysis

Figure 5. Forest plot: exercise & education

Figure 6 Forest plot: education alone

For peer review only

For peer review only - http://bmjopen.bmj.com/site/about/guidelines.xhtml

Table 1. Characteristics of studies evaluating pre-operative exercise and education

Publication<br>Location<br>Date of study	Indication<br>Number randomised (intervention : control)<br>Mean age (%female)	Primary focus of intervention	Study setting<br>Timing, duration and intensity	Control group care	Longest follow up after surgery<br>Losses to follow up (intervention: control)<br>Risk of bias	Outcomes
Beaupre et al, 2004<br>Canada<br>Date not specified	Non-inflammatory arthritis<br>N= 131<br>(65:66)<br>67 years (55%)	Multifactorial: exercise (gait re-education, functional, ROM, strengthening) and education on crutch walking, bed mobility and postoperative ROM	Community physical therapy clinic group<br>3-times per week for 4-weeks, 6 weeks before surgery	No intervention	6 and 12 months<br>16 (10:6) patients cancelled surgery, 6 (4:2) patients lost to 12 month follow up<br>Low overall risk of bias	No difference in WOMAC pain or SF-36 bodily pain at 6 and 12 months (p>0.05)<br>General complications, "similarly dispersed across both groups": Pulmonary embolism (2:2), DVT (3:6), Superficial infection (2:3), Deep infection (1:0), Manipulation (1:2)
Culliton et al. 2018<br>Canada<br>2013-2014	Osteoarthritis<br>N= 416 (207:209)<br>64 years (43%)	Education: access to an online e-learning tool during pre-admission visit and 31-page guide also provided to the control group. Tool consisted of custom-made 32 videos (1-2 minutes) addressing patient expectations, presented by surgeons, physical therapists and patients	Access to e-learning tool remained active until the study end (1 year). Tool consisted of custom-made 32 videos (1-2 mins) addressing patient expectations, presented by surgeons, physical therapists and patients	Booklet alone	12 months post-operative<br>Data analysed for 76.6 % of original sample randomised. Reasons include TKR not taking place or lack of email address. Later exclusions due to failure to submit data.<br>Low overall risk of bias	1 year postoperative, significant between-group differences in favour of the control group for the KOOS symptoms score (p= 0.04). Pain scores obtained from authors directly were not significantly different between groups.<br>Adverse events not reported.

For peer review only - http://bmjopen.bmj.com/site/about/guidelines.xhtml

D'Lima et al. 1996<br>Cardiovascular conditioning<br>USA<br>Date not specified	Rheumatoid arthritis or osteoarthritis<br>N=20 (10:10)<br>70.6 years (35%)	Exercise (stretching, strengthening, cardiovascular conditioning)	Hospital. Individualised<br>45 minute sessions, 3 times a week for 18 weeks commencing 6 weeks before surgery	One meeting with physical therapist	24 and 48 weeks<br>No losses to follow up reported<br>High risk of bias: small numbers of patients; baseline demographic differences; failure to report data other than graphically	Hospital for Special Surgery Knee HSS pain score improved in intervention group compared with control but not statistically significant<br>Sleep apnoea (1:0), Manipulation (1:0), Deep infection (0:1), Intestinal pseudo-obstruction (0:1)
D'Lima et al. 1996<br>Physical therapy<br>USA<br>Date not specified	Rheumatoid arthritis or osteoarthritis<br>N=20 (10:10)<br>69.0 years (60%)	Exercise (strengthening, range of motion)	Hospital. 1 on 1 programme<br>45 minute sessions, 3 times a week for 18 weeks commencing 6 weeks before surgery	One meeting with physical therapist	24 and 48 weeks<br>No losses to follow up reported<br>High risk of bias: small numbers of patients; baseline demographic differences	Hospital for Special Surgery Knee Rating pain score improved in intervention group compared with control but not statistically significant<br>Atrial fibrillation (1:0), Paroxysmal tachycardia (1:0), Deep infection (0:1), Intestinal pseudo-obstruction (0:1)
Fernandes et al. 2017<br>Denmark<br>2010-2011<br>1 hospital	Osteoarthritis<br>165 (84; 81) hip or knee replacement<br>Mean 67.9 (SD 8.6); 66.9 (8.3)<br>(56%)	Exercise (neuromuscular focusing on lower extremity muscular control and quality of movement)	Hospital: group-based, physiotherapist led<br>Mean 13.1 one-hour sessions twice per week during 8 weeks before surgery.	Standard care	61 weeks<br>2; 5<br>Low risk of bias	Mean differences at 61 weeks favoured the exercise group for all KOOS subscales, but only significantly for quality of life subscale. Disaggregated long-term data for knee replacement acquired by personal contact<br>0; 2 deep infection
Huber et al. 2015<br>Switzerland<br>2009-2012	Osteoarthritis<br>N=45 (22:23)<br>70.4 years (47%)	Exercise (neuromuscular including functional and strengthening)	Hospital: group based<br>8 or more sessions commencing 6-12 weeks before surgery.	All patients attended "knee school" on 3 occasions from about 4 weeks	12 months<br>9 (5:4) lost to follow up<br>Low risk of bias	No difference in KOOS pain or SF-36 bodily pain between groups at 12 months<br>1 intervention patient had a joint specific adverse event

For peer review only

For peer review only - http://bmjopen.bmj.com/site/about/guidelines.xhtml

				before surgery		
Matassi et al. 2014<br>Belgium<br>2005-2006	Osteoarthritis<br>N=122 (61:61)<br>66.5 years (48%)	Exercise (muscle strength and flexibility)	Home<br>5 days per week for 6 weeks commencing 6 weeks before surgery	Maintained regular activities	1 year<br>No losses to follow up reported<br>High risk of bias due to selective outcome reporting; outcome also not patient reported	No significant effect of exercise on the Knee Society Clinical Rating System knee score or patient function score<br>Manipulation (5:3). In the intervention group 1 patient developed ipsilateral adductor tendinitis and 1 patient stopped exercises due to increased knee pain
Rooks et al. 2006<br>USA<br>2001-2003	Osteoarthritis<br>N=45 (22:23)<br>67.0 years (53%)	Exercise (strengthening, flexibility, cardiovascular, pool exercises)	Community fitness facility<br>3 times per week for 6 weeks 30-60 mins each increasing intensity from 4-6 weeks	2 education mailings and 3 telephone calls	26 weeks<br>16 (8:8) lost to follow up<br>High risk of bias: large loss to follow up	Statistically significant greater improvement in SF-36 bodily pain at 26 weeks (p<0.05) but no difference for WOMAC pain<br>Perioperative complications (3:4). No serious adverse events in either group requiring hospital admission
Tungtrongjit et al. 2012<br>Thailand<br>2011-2012	Osteoarthritis<br>N=68 (30:30 in analyses)<br>64.5 years (17%)	Exercise (quadriceps strengthening)	Home<br>3 times per day for 3 weeks commencing 3 weeks before surgery	Asked to continue normal activities	6 months<br>8 patients excluded from analyses due to adverse events and loss to follow up<br>Possible risk of bias: unclear sequence generation; data provided only on patients followed up	WOMAC pain improved in intervention compared with controls (p= 0.029) but not VAS pain (p=0.137)<br>Across all randomised patients there was 1 surgical wound infection, 1 post-operative knee trauma, and 2 cases of post-operative wound dehiscence

WOMAC Western Ontario and McMaster Universities Osteoarthritis Index; SF-36 Short Form 36; VAS Visual analogue scale; KOOS Knee disability and Osteoarthritis Outcome Score; EQ-5D European Quality of Life-5 Dimensions

For peer review only - http://bmjopen.bmj.com/site/about/guidelines.xhtml

Table 2

Summary of findings: Exercise alone compared to standard care for knee replacement (long-term outcome only)

Patient or population: Adult patients scheduled for total knee replacement
Intervention: 3- 12 weeks exercise conducted in the presurgical period
Setting: Clinic-supervised exercise (5 studies); home-based exercise (2 studies)
Comparison: Standard care

Outcome<br>№ of participants<br>(studies)	Relative effect (95%CI)	**Illustrative comparative risks (95% CI)**		Certainty	Comments
**Long-term pain**<br><br>Assessed with: Various scales (WOMAC[a], KOOS[b], HSSK[c])<br>Follow up: range 6 months to 12 months<br><br>№ of participants in metaanalysis: 229 (6 RCTs)<br><br>№ of participants not in metaanalysis: 122 (1 RCT).	**Metaanalysis**:<br><br>SMD **0.2 higher** (-0.06 - 0.47)<br><br>**Narrative**<br><br>"Non significant difference"	Mean KOOS scores in the untreated group ranged from 73.2 to 85.95 in the control group; mean HSSK scores in the control group were 27; mean WOMAC scores in control group were<br><br>-2.3 to -4.8	The mean level of pain after exercise was 0.20 standard deviations lower (-0.06 to 0.47 lower).	⊕⊕⊕◯<br>MODERATE [d,e]	There was no clear evidence of a difference between exercise and standard care for long-term pain either from data from the metaanalysis or from a comparatively large study not included within the metaanalysis.<br><br>We rescaled the SMD finding to a commonly used instrument in the field - the KOOS, using estimations based on the mean of a representative study within the analysis (Huber 2015) and our pooled SMD of 0.02 (-0.06 to 0.47 higher). This indicates that exercise led to a mean increase of 2.7 units/points on the KOOS (95% CI -0.8 to 6.29) in the treated group.<br><br>Sensitivity analysis of two studies at low risk of bias suggested a smaller effect (mean increase of 1.46 points on the KOOS (95% CI-4.58 to 7.50)
**Adverse events**	See comment	See comment	See comment	⊕⊕◯◯<br>LOW [d,f]	Two studies reported treatment-related adverse events (increase in pain) separately from the main pain outcomes, which occurred during the period of intervention. Information on perioperative complications e.g. superficial and deep infections, were reported in all studies. Events were few and attribution to treatment status difficult

CI: Confidence interval; **MD:** Mean difference; **SMD:** Standardised mean difference

For peer review only - http://bmjopen.bmj.com/site/about/guidelines.xhtml

Summary of findings: Exercise alone compared to standard care for knee replacement (long-term outcome only)

Patient or population: Adult patients scheduled for total knee replacement
Intervention: 3- 12 weeks exercise conducted in the presurgical period
Setting: Clinic-supervised exercise (5 studies); home-based exercise (2 studies)
Comparison: Standard care

Outcome<br>№ of participants<br>(studies)	Relative effect (95%CI)	Illustrative comparative risks (95% CI)		Certainty	Comments

GRADE Working Group grades of evidence
High certainty: We are very confident that the true effect lies close to that of the estimate of the effect
Moderate certainty: We are moderately confident in the effect estimate: The true effect is likely to be close to the estimate of the effect, but there is a possibility that it is substantially different
Low certainty: Our confidence in the effect estimate is limited: The true effect may be substantially different from the estimate of the effect
Very low certainty: We have very little confidence in the effect estimate: The true effect is likely to be substantially different from the estimate of effect

Explanations

a. WOMAC = The Western Ontario and McMaster Universities Osteoarthritis Index (WOMAC) (Bellamy et al 1988). The original pain scale runs from 0 to 20 and a higher number indicates a greater pain. There are several modifications. Some triallists also reverse the polarity and/or transform responses into a 0-100 scale where a high number indicates less pain. We standardised to this latter method as it was most common and was similar to the KOOS and the HSSK.
b. KOOS = Knee Injury and Osteoarthritis Outcome Score (Roos et al 1998) . The scale ranges from 0 – 100 and a higher number indicates less pain
c. HSSK = Hospital Society Score (Insall et al 1976). Pain is measured on a scale of 0 to 30, with a high number indicating less pain.
d. Downgraded due to risk of bias associated with lack of blinding of both personnel and participants in all studies also concerns about sequence generation /allocation concealment in three studies.
e. We did not downgrade for imprecision as findings of the one large study not included in metaanalysis were very similar to the six studies included in metaanalysis
f. We downgraded the quality of evidence for study limitations, in particular imprecision: estimate based on few events

For peer review only

Summary of findings: Exercise & education compared to standard care for knee replacement (long-term outcome only)

Patient or population: Adult patients scheduled for total knee replacement
Setting: Clinic-supervised exercise Clinics (1 study)
Intervention: 1 session of education combined with 4 weeks' exercise in the pre-operative period
Comparison: TAU

Outcome<br>№ of participants<br>(studies)	Relative effect (95%CI)	Illustrative comparative risks (95% CI)		Certainty	Comments
**Long-term pain**<br>Assessed with: WOMAC[a] scale<br>Follow up: 12 months<br>№ of participants: 109<br>(1 RCT)	**Single study results**:<br>MD **2 units higher**<br>(3.45 lower to 7.45 higher) -	Mean score of untreated control group was 80 on the WOMAC (sd = 16)	Exercise and education resulted in a mean score of 82 on the WOMAC (sd= 13)	⊕⊕◯◯<br>LOW [b,c,d]	)
**Adverse events**	See comment	See comment	See comment	⊕⊕⊕◯<br>VERY LOW [b,c,d,e]	General complications, "similarly dispersed across both groups": Pulmonary embolism (2:2), DVT (3:6), Superficial infection (2:3), Deep infection (1:0), Manipulation (1:2)

CI: Confidence interval; **MD:** Mean difference

GRADE Working Group grades of evidence
High certainty: We are very confident that the true effect lies close to that of the estimate of the effect
Moderate certainty: We are moderately confident in the effect estimate: The true effect is likely to be close to the estimate of the effect, but there is a possibility that it is substantially different
Low certainty: Our confidence in the effect estimate is limited: The true effect may be substantially different from the estimate of the effect
Very low certainty: We have very little confidence in the effect estimate: The true effect is likely to be substantially different from the estimate of effect

Explanations

a. WOMAC = The Western Ontario and McMaster Universities Osteoarthritis Index (WOMAC) (Bellamy et al 1988). The original pain scale runs from 0 to 20 and a higher number indicates a greater pain. There are several modifications. Some triallists also reverse the polarity and/or transform responses into a 0-100 scale where a high number indicates less pain. We standardised to this latter method as it was most common and was similar to the KOOS and the HSSK.
b. Downgraded due to risk of bias due to lack of blinding of participants or personnel.
c. Downgraded for imprecision due to being one study with less than an optimal information sample size
d. There were too few studies to reliably assess risk of publication bias or other sources of small study bias
e. We downgraded the quality of evidence for study limitations, in particular imprecision: estimate based on few events

For peer review only - http://bmjopen.bmj.com/site/about/guidelines.xhtml

Summary of findings: Exercise & education compared to standard care for knee replacement (long-term outcome only)

Patient or population: Adult patients scheduled for total knee replacement **Setting**: Clinic-supervised exercise Clinics (1 study)
Intervention: 1 session of education combined with 4 weeks' exercise in the pre-operative period **Comparison**: TAU

Outcome<br>№ of participants<br>(studies)	Relative effect (95%CI)	Illustrative comparative risks (95% CI)		Certainty	Comments

Summary of findings: Education compared to standard care for knee replacement (long term outcome only)

Patient or population: Adult patients scheduled for total knee replacement **Setting**: Clinic followed by online access (1 study)
Intervention: Access to online elearning tool with active demonstration at presurgical visit followed by email prompts **Comparison**: TAU

Outcome<br>№ of participants<br>(studies)	Relative effect (95%CI)	Illustrative comparative risks (95% CI)		Certainty	Comments
**Longterm pain**<br><br>Assessed with: KOOS scale[a]<br>Follow up: 12 months<br>№ of participants: 319<br>(1 RCT)	**Single study results**:<br><br>MD **2.55 lower**<br>(6.35 lower to 1.24 higher)	Mean score of untreated control group was 82.62 on the KOOS (sd = 15.4)	Exercise and education resulted in a decrease on the KOOS, leading to a mean score in the treated group of 80.01(sd= 19)	⊕⊕⊕◯<br>MODERATE [b,c]	
**Adverse events**	See comment	See comment	See comment		No data on adverse events appear to have been sought within the one study within this comparison

CI: Confidence interval; **MD:** Mean difference

GRADE Working Group grades of evidence
High certainty: We are very confident that the true effect lies close to that of the estimate of the effect
Moderate certainty: We are moderately confident in the effect estimate: The true effect is likely to be close to the estimate of the effect, but there is a possibility that it is substantially different
Low certainty: Our confidence in the effect estimate is limited: The true effect may be substantially different from the estimate of the effect
Very low certainty: We have very little confidence in the effect estimate: The true effect is likely to be substantially different from the estimate of effect

For peer review only - http://bmjopen.bmj.com/site/about/guidelines.xhtml

Summary of findings: Exercise & education compared to standard care for knee replacement (long-term outcome only)

Patient or population: Adult patients scheduled for total knee replacement
Intervention: 1 session of education combined with 4 weeks' exercise in the pre-operative period
Setting: Clinic-supervised exercise Clinics (1 study)
Comparison: TAU

Outcome № of participants (studies)	Relative effect (95%CI)	Illustrative comparative risks (95% CI)		Certainty	Comments

Explanations

a. KOOS = Knee Injury and Osteoarthritis Outcome Score (Roos et al 1998) . The scale ranges from 0 – 100 and a higher number indicates less pain
b. Downgraded due to risk of bias due to lack of blinding of participants or personnel and high loss to followup (24%)
c. There were too few studies to reliably assess risk of publication bias or other sources of small study bias

For peer review only - http://bmjopen.bmj.com/site/about/guidelines.xhtml

References

1. McAlindon TE, Bannuru RR, Sullivan MC, Arden NK, Berenbaum F, Bierma-Zeinstra SM, et al. OARSI guidelines for the non-surgical management of knee osteoarthritis. Osteoarthritis Cart. 2014;22(3):363-88.

2. Osteoarthritis: care and management. London: National Institute for Health and Clinical Excellence; 2014.

3. National Joint Registry for England, Wales, Northern Ireland and the Isle of Man: 15th Annual Report. Hemel Hempstead: NJR Centre 2018.

4. Scottish Arthroplasty Project. Annual report 2018. Edinburgh: NHS National Services Scotland; 2018.

5. Maradit Kremers H, Larson DR, Crowson CS, Kremers WK, Washington RE, Steiner CA, et al. Prevalence of total hip and knee replacement in the United States. J Bone Joint Surg (Am). 2015;97(17):1386-97.

6. Beswick AD, Wylde V, Gooberman-Hill R, Blom A, Dieppe P. What proportion of patients report long-term pain after total hip or knee replacement for osteoarthritis? A systematic review of prospective studies in unselected patients. BMJ Open. 2012;2(1):e000435.

7. Nilsdotter AK, Toksvig-Larsen S, Roos EM. Knee arthroplasty: are patients' expectations fulfilled? A prospective study of pain and function in 102 patients with 5-year follow-up. Acta Orthop. 2009;80(1):55-61.

8. Scott CE, Howie CR, MacDonald D, Biant LC. Predicting dissatisfaction following total knee replacement: a prospective study of 1217 patients. J Bone Joint Surg (Br). 2010;92(9):1253-8.

9. Sayers A, Wylde V, Lenguerrand E, Beswick AD, Gooberman-Hill R, Pyke M, et al. Rest pain and movement-evoked pain as unique constructs in hip and knee replacements. Arthritis Care Res. 2016;68(2):237-45.

10. Blom AW, Artz N, Beswick AD, Burston A, Dieppe P, Elvers KT, et al. Improving patients' experience and outcome of total joint replacement: The RESTORE programme. Programme Grants Appl Res. 2016;4(12).

11. McDonald S, Hetrick S, Green S. Pre-operative education for hip or knee replacement. Cochrane Database Syst Rev. 2004(1):CD003526.

12. Ackerman IN, Bennell KL. Does pre-operative physiotherapy improve outcomes from lower limb joint replacement surgery? A systematic review. Aust J Physiother. 2004;50(1):25-30.

13. Valkenet K, van de Port IG, Dronkers JJ, de Vries WR, Lindeman E, Backx FJ. The effects of preoperative exercise therapy on postoperative outcome: a systematic review. Clin Rehabil. 2011;25(2):99-111.

For peer review only

14. Wallis JA, Taylor NF. Pre-operative interventions (non-surgical and non-pharmacological) for patients with hip or knee osteoarthritis awaiting joint replacement surgery--a systematic review and meta-analysis. Osteoarthritis Cart. 2011;19(12):1381-95.
15. Silkman Baker C, McKeon JM. Does preoperative rehabilitation improve patient-based outcomes in persons who have undergone total knee arthroplasty? A systematic review. PM R. 2012;4(10):756-67.
16. Hoogeboom TJ, Oosting E, Vriezekolk JE, Veenhof C, Siemonsma PC, de Bie RA, et al. Therapeutic validity and effectiveness of preoperative exercise on functional recovery after joint replacement: a systematic review and meta-analysis. PLoS One. 2012;7(5):e38031.
17. Gill SD, McBurney H. Does exercise reduce pain and improve physical function before hip or knee replacement surgery? A systematic review and meta-analysis of randomized controlled trials. Arch Phys Med Rehabil. 2013;94(1):164-76.
18. Shoemaker MJ, Gibson C, Saagman S. Preoperative exercise in individuals undergoing total knee arthroplasty state of the evidence. Topics in Geriatric Rehabilitation. 2013;29(1):2-16.
19. Simmons L ST. Effectiveness of pre-operative physiotherapy-based programmes on outcomes following total knee arthroplasty: a systematic review and meta-analysis. Physical Therapy Reviews. 2013;18(1):1-10.
20. Santa Mina D, Clarke H, Ritvo P, Leung YW, Matthew AG, Katz J, et al. Effect of total-body prehabilitation on postoperative outcomes: a systematic review and meta-analysis. Physiotherapy. 2014;100(3):196-207.
21. Jordan RW, Smith NA, Chahal GS, Casson C, Reed MR, Sprowson AP. Enhanced education and physiotherapy before knee replacement; is it worth it? A systematic review. Physiotherapy. 2014;100(4):305-12.
22. Cabilan CJ, Hines S, Munday J. The effectiveness of prehabilitation or preoperative exercise for surgical patients: a systematic review. JBI Database System Rev Implement Rep. 2015;13(1):146-87.
23. Aydin D, Klit J, Jacobsen S, Troelsen A, Husted H. No major effects of preoperative education in patients undergoing hip or knee replacement--a systematic review. Dan Med J. 2015;62(7).
24. Kwok IH, Paton B, Haddad FS. Does pre-operative physiotherapy improve outcomes in primary total knee arthroplasty? - A systematic review. J Arthroplasty. 2015;30(9):1657-63.
25. Peer MA, Rush R, Gallacher PD, Gleeson N. Pre-surgery exercise and post-operative physical function of people undergoing knee replacement surgery: A systematic review and meta-analysis of randomized controlled trials. J Rehabil Med. 2017;49(4):304-15.

For peer review only

26. Zhang H, Hao X, Shuai T, Deng Y, Zeng Z, Song G. Efficacy of preoperative rehabilitation for total knee replacement: a systematic review. Chinese Journal of Evidence-Based Medicine. 2017;17(9):1060-5.
27. Chen H, Li S, Ruan T, Liu L, Fang L. Is it necessary to perform prehabilitation exercise for patients undergoing total knee arthroplasty: meta-analysis of randomized controlled trials. Physician and Sportsmedicine. 2018;46(1):36-43.
28. Chesham RA, Shanmugam S. Does preoperative physiotherapy improve postoperative, patient-based outcomes in older adults who have undergone total knee arthroplasty? A systematic review. Physiother Theory Pract. 2017;33(1):9-30.
29. Wang L, Lee M, Zhang Z, Moodie J, Cheng D, Martin J. Does preoperative rehabilitation for patients planning to undergo joint replacement surgery improve outcomes? A systematic review and meta-analysis of randomised controlled trials. BMJ Open. 2016;6(2):e009857.
30. Wylde V, Dennis J, Gooberman-Hill R, Beswick AD. Effectiveness of postdischarge interventions for reducing the severity of chronic pain after total knee replacement: systematic review of randomised controlled trials. BMJ Open. 2018;8(2):e020368.
31. Beswick AD, Dennis J, Gooberman-Hill R, Blom AW, Wylde V. Are perioperative interventions effective in preventing chronic pain after primary total knee replacement? A systematic review. BMJ Open. 2019;9(9):e028093.
32. Beswick A, Dennis J, Gooberman-Hill R, Wylde V, Bertram W. The effectiveness of pre-, peri- and post-operative interventions in preventing chronic pain in patients receiving primary total knee replacement: a systematic review. PROSPERO. 2017.
33. Richardson WS, Wilson MC, Nishikawa J, Hayward RS. The well-built clinical question: a key to evidence-based decisions. ACP J Club. 1995;123(3):A12-3.
34. Higgins JPT, Green S. Cochrane Handbook for Systematic Reviews of Interventions Version 5.1.0 [Updated March 2011]: The Cochrane Collaboration; 2011. Available from: http://www.cochrane-handbook.org.
35. Moher D, Liberati A, Tetzlaff J, Altman DG. Preferred reporting items for systematic reviews and meta-analyses: the PRISMA statement. J Clin Epidemiol. 2009;62(10):1006-12.
36. Gooberman-Hill R, Burston A, Clark E, Johnson E, Nolan S, Wells V, et al. Involving patients in research: considering good practice. Musculoskeletal Care. 2013;11(4):187-90.
37. Murray DW, Fitzpatrick R, Rogers K, Pandit H, Beard DJ, Carr AJ, et al. The use of the Oxford hip and knee scores. J Bone Joint Surg (Br). 2007;89(8):1010-4.
38. Bellamy N, Buchanan W, Goldsmith C, Campbell J, Stitt L. Validation study of WOMAC: a health status instrument for measuring clinically-important patient-relevant outcomes following total hip or knee arthroplasty in osteoarthritis. J Orthop Rheumatol. 1988;1.

For peer review only

For peer review only - http://bmjopen.bmj.com/site/about/guidelines.xhtml

39. Roos EM, Roos HP, Lohmander LS, Ekdahl C, Beynnon BD. Knee Injury and Osteoarthritis Outcome Score (KOOS)--development of a self-administered outcome measure. J Orthop Sports Phys Ther. 1998;28(2):88-96.

40. Insall JN, Dorr LD, Scott RD, Scott WN. Rationale of the Knee Society clinical rating system. Clin Orthop Relat Res. 1989(248):13-4.

41. Insall JN, Ranawat CS, Aglietti P, Shine J. A comparison of four models of total knee-replacement prostheses. J Bone Joint Surg (Am). 1976;58(6):754-65.

42. Wylde V, Bruce J, Beswick A, Elvers K, Gooberman-Hill R. The assessment of chronic post-surgical pain after knee replacement: A systematic review. Arthritis Care Res. 2013;65(11):1795-803.

43. Hewlett SA. Patients and clinicians have different perspectives on outcomes in arthritis. J Rheumatol. 2003;30(4):877-9.

44. Bachmeier CJM, March LM, Cross MJ, Lapsley HM, Tribe KL, Courtenay BG, et al. A comparison of outcomes in osteoarthritis patients undergoing total hip and knee replacement surgery. Osteoarthritis Cart. 2001;9(2):137-46.

45. Ware JE, Jr., Sherbourne CD. The MOS 36-item short-form health survey (SF-36). I. Conceptual framework and item selection. Med Care. 1992;30(6):473-83.

46. Ware J, Jr., Kosinski M, Keller SD. A 12-Item Short-Form Health Survey: construction of scales and preliminary tests of reliability and validity. Med Care. 1996;34(3):220-33.

47. Dworkin RH, Turk DC, Farrar JT, Haythornthwaite JA, Jensen MP, Katz NP, et al. Core outcome measures for chronic pain clinical trials: IMMPACT recommendations. Pain. 2005;113(1-2):9-19.

48. Moore RA, Eccleston C, Derry S, Wiffen P, Bell RF, Straube S, et al. "Evidence" in chronic pain--establishing best practice in the reporting of systematic reviews. Pain. 2010;150(3):386-9.

49. Higgins JPT, Altman DG, Gøtzsche PC, Jüni P, Moher D, Oxman AD, et al. The Cochrane Collaboration's tool for assessing risk of bias in randomised trials. BMJ. 2011;343:d5928.

50. Review Manager (RevMan) [Computer program]. Version 5.3. Copenhagen: The Nordic Cochrane Centre: The Cochrane Collaboration; 2014.

51. Guyatt G, Oxman AD, Akl EA, Kunz R, Vist G, Brozek J, et al. GRADE guidelines: 1. Introduction-GRADE evidence profiles and summary of findings tables. J Clin Epidemiol. 2011;64(4):383-94.

52. Villebro NM, Pedersen T, Moller AM, Tonnesen H. Long-term effects of a preoperative smoking cessation programme. Clin Respir J. 2008;2(3):175-82.

For peer review only

53. Amory JK, Chansky HA, Chansky KL, Camuso MR, Hoey CT, Anawalt BD, et al. Preoperative supraphysiological testosterone in older men undergoing knee replacement surgery. J Am Geriatr Soc. 2002;50(10):1698-701.
54. Moonen AFCM, Thomassen BJW, Knoors NT, van Os JJ, Verburg AD, Pilot P. Pre-operative injections of epoetin-alpha versus post-operative retransfusion of autologous shed blood in total hip and knee replacement: a prospective randomised clinical trial. J Bone Joint Surg (Br). 2008;90(8):1079-83.
55. Sousa RJG, Barreira PMB, Leite PTS, Santos ACM, Ramos MHSS, Oliveira AF. Preoperative staphylococcus aureus screening/decolonization protocol before total joint arthroplasty-results of a small prospective randomized trial. J Arthroplasty. 2016;31(1):234-9.
56. D'Lima DD, Colwell CW, Jr., Morris BA, Hardwick ME, Kozin F. The effect of preoperative exercise on total knee replacement outcomes. Clin Orthop Relat Res. 1996(326):174-82.
57. Beaupre LA, Lier D, Davies DM, Johnston DBC. The effect of a preoperative exercise and education program on functional recovery, health related quality of life, and health service utilization following primary total knee arthroplasty. J Rheumatol. 2004;31(6):1166-73.
58. Fernandes L, Roos E, Overgaard S, Villadsen A, Sogaard R. Supervised neuromuscular exercise prior to hip and knee replacement: 12-month clinical effect and cost-utility analysis alongside a randomised controlled trial. BMC Musculoskelet Disord. 2017;18(1):5.
59. Huber EO, Roos EM, Meichtry A, de Bie RA, Bischoff-Ferrari HA. Effect of preoperative neuromuscular training (NEMEX-TJR) on functional outcome after total knee replacement: an assessor-blinded randomized controlled trial. BMC Musculoskelet Disord. 2015;16(101).
60. Matassi F, Duerinckx J, Vandenneucker H, Bellemans J. Range of motion after total knee arthroplasty: the effect of a preoperative home exercise program. Knee Surg Sports Traumatol Arthrosc. 2014;22(3):703-9.
61. Tungtrongjit Y, Weingkum P, Saunkool P. The effect of preoperative quadriceps exercise on functional outcome after total knee arthroplasty. J Med Assoc Thai. 2012;95 Suppl 10:S58-66.
62. Rooks DS, Huang J, Bierbaum BE, Bolus SA, Rubano J, Connolly CE, et al. Effect of preoperative exercise on measures of functional status in men and women undergoing total hip and knee arthroplasty. Arthritis Rheum. 2006;55(5):700-8.
63. Culliton SE, Bryant DM, MacDonald SJ, Hibbert KM, Chesworth BM. Effect of an e-learning tool on expectations and satisfaction following total knee arthroplasty: A randomized controlled trial. J Arthroplasty. 2018;33(7):2153-8.

For peer review only - http://bmjopen.bmj.com/site/about/guidelines.xhtml

64. Ageberg E, Link A, Roos EM. Feasibility of neuromuscular training in patients with severe hip or knee OA: The individualized goal-based NEMEX-TJR training program. BMC Musculoskelet Disord. 2010;11(1):126.
65. Jacobs CA, Christensen CP. Correlations between Knee Society function scores and functional force measures. Clin Orthop Relat Res. 2009;467(9):2414-9.
66. Gademan MGJ, Hofstede SN, Vliet Vlieland TPM, Nelissen RGHH, Marang-van de Mheen PJ. Indication criteria for total hip or knee arthroplasty in osteoarthritis: a state-of-the-science overview. BMC Musculoskelet Disord. 2016;17(1):463.
67. Hamilton DF, Lane JV, Gaston P, Patton JT, MacDonald D, Simpson AHRW, et al. What determines patient satisfaction with surgery? A prospective cohort study of 4709 patients following total joint replacement. BMJ Open. 2013;3(4):e002525.
68. Wylde V, Dennis J, Beswick AD, Bruce J, Eccleston C, Howells N, et al. Systematic review of management of chronic pain after surgery. Br J Surg. 2017;104(10):1293-306.
69. McDonald S, Page MJ, Beringer K, Wasiak J, Sprowson A. Preoperative education for hip or knee replacement. Cochrane Database Syst Rev. 2014(5):CD003526.
70. Goldsmith LJ, Suryaprakash N, Randall E, Shum J, MacDonald V, Sawatzky R, et al. The importance of informational, clinical and personal support in patient experience with total knee replacement: a qualitative investigation. BMC Musculoskelet Disord. 2017;18(1):127.
71. Edwards RR, Haythornthwaite JA, Smith MT, Klick B, Katz JN. Catastrophizing and depressive symptoms as prospective predictors of outcomes following total knee replacement. Pain Res Manag. 2009;14(4):307-11.
72. Riddle DL, Keefe FJ, Ang DC, Slover J, Jensen MP, Bair MJ, et al. Pain coping skills training for patients who catastrophize about pain prior to knee arthroplasty: A multisite randomized clinical trial. J Bone Joint Surg (Am). 2019;101(3):218-27.

For peer review only

For peer review only - http://bmjopen.bmj.com/site/about/guidelines.xhtml

PRISMA 2009 Flow Diagram

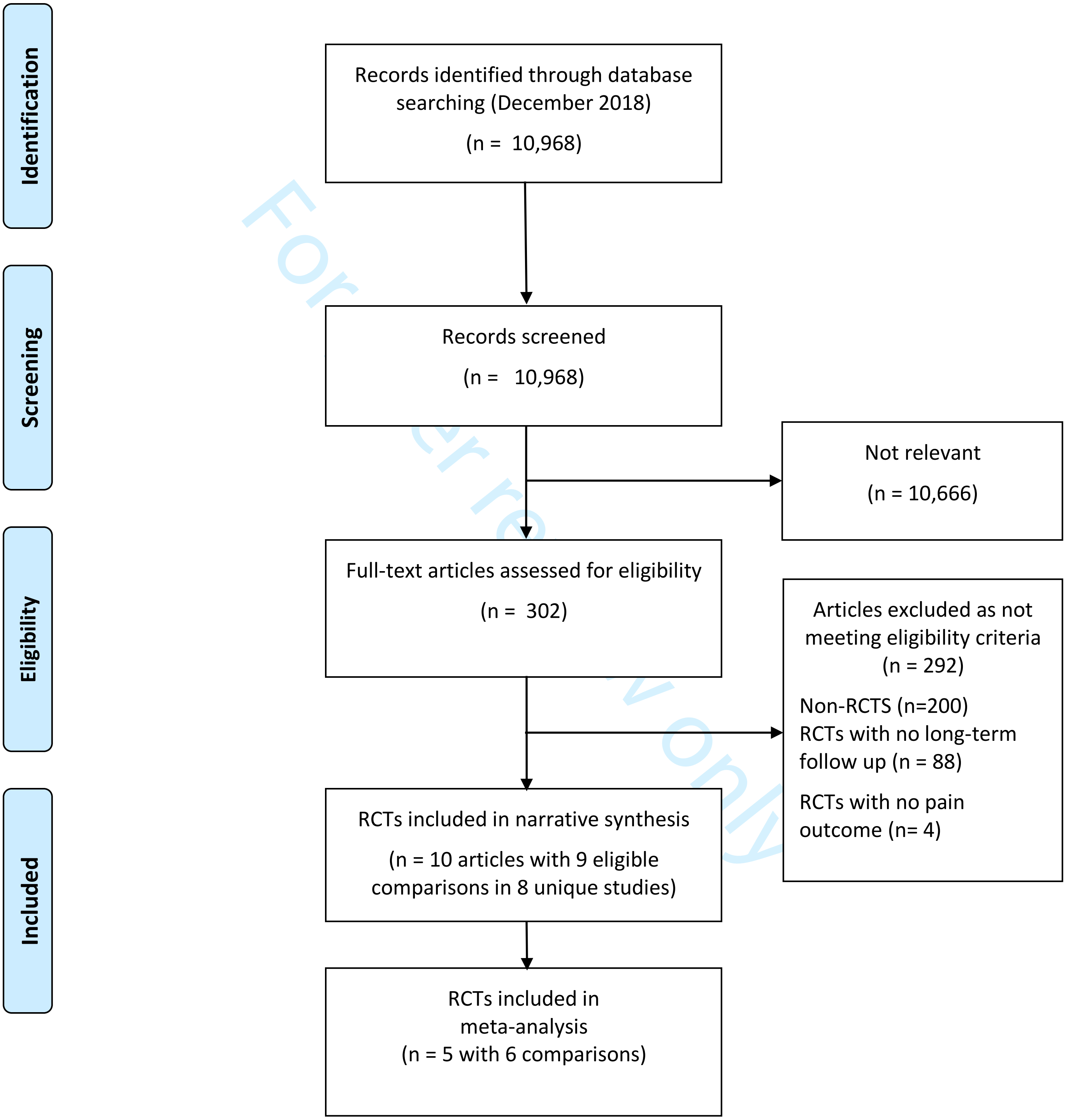

For peer review only

	Random sequence generation (selection bias)	Allocation concealment (selection bias)	Blinding of participants and personnel	Blinding of outcome assessors	Incomplete outcome data (attrition bias)	Selective reporting (reporting bias)	Other bias
Beaupre 2004 (education plus exercise)	+	+	−	−	+	?	+
Culliton 2018 (education)	+	?	−	−	?	+	+
D'Lima 1996 (cardio training)	+	?	−	?	+	−	−
D'Lima 1996 (physio training)	+	?	−	?	+	−	−
Huber 2015 (NEMEX-TJR)	+	+	−	?	+	+	?
Matassi 2014 home ex	+	?	−	−	+	−	?
Rooks 2006 comm ex	?	?	−	−	−	?	+
Tungtrongjit 2012 home ex	?	?	−	−	?	?	+
Villadsen 2014 (NEMEX-TJR)	+	+	−	−	+	+	+

	Exercise			Standard care				Std. Mean Difference
**Study or Subgroup**	**Mean**	**SD**	**Total**	**Mean**	**SD**	**Total**	**Weight**	**IV, Random, 95% CI**
**1.1.1 Pain at 6 - 12 months (various scales)**								
D'Lima 1996 (cardio training)	28.7	16.02	10	27	16.02	5	6.0%	0.10 [-0.97, 1.17]
D'Lima 1996 (physio training)	29	16.02	10	27	16.03	5	6.0%	0.12 [-0.96, 1.19]
Rooks 2006 comm ex	-2.4	2.7	14	-2.3	2	15	13.0%	-0.04 [-0.77, 0.69]
Fernandes 2017 NEMEX-TJR	74.2	18.6	37	73.2	21.7	37	33.3%	0.05 [-0.41, 0.50]
Huber 2015 NEMEX-TJR	87.76	11.09	17	85.95	13.39	19	16.1%	0.14 [-0.51, 0.80]
Tungtrongjit 2012 home ex	-2.5	3.1	30	-4.8	4.2	30	25.7%	0.62 [0.10, 1.13]
**Subtotal (95% CI)**			**118**			**111**	**100.0%**	**0.20 [-0.06, 0.47]**

Heterogeneity: $Tau^2 = 0.00$; $Chi^2 = 3.39$, df = 5 (P = 0.64); $I^2 = 0\%$

Test for overall effect: Z = 1.53 (P = 0.13)

Test for subgroup differences: Not applicable

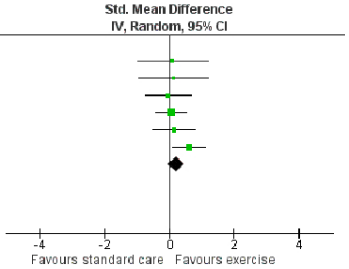

	Exercise			Standard care				Std. Mean Difference
**Study or Subgroup**	**Mean**	**SD**	**Total**	**Mean**	**SD**	**Total**	**Weight**	**IV, Random, 95% CI**
**2.1.1 Pain at 12 months (KOOS)**								
Fernandes 2017 NEMEX-TJR	74.2	18.6	37	73.2	21.7	37	67.4%	0.05 [-0.41, 0.50]
Huber 2015 NEMEX-TJR	87.76	11.09	17	85.95	13.39	19	32.6%	0.14 [-0.51, 0.80]
**Subtotal (95% CI)**			**54**			**56**	**100.0%**	**0.08 [-0.29, 0.45]**

Heterogeneity: $Tau^2 = 0.00$; $Chi^2 = 0.05$, $df = 1$ ($P = 0.82$); $I^2 = 0\%$

Test for overall effect: $Z = 0.42$ ($P = 0.68$)

Test for subgroup differences: Not applicable

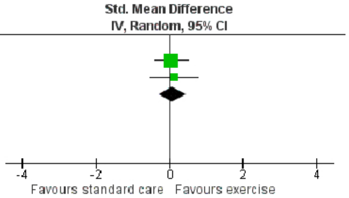

	Exercise & education			Standard care				Std. Mean Difference
**Study or Subgroup**	**Mean**	**SD**	**Total**	**Mean**	**SD**	**Total**	**Weight**	**IV, Random, 95% CI**
**3.1.2 Pain at 12 months (WOMAC)**								
Beaupre 2004 (education plus exercise)	82	13	51	80	16	58	100.0%	0.14 [-0.24, 0.51]
**Subtotal (95% CI)**			**51**			**58**	**100.0%**	**0.14 [-0.24, 0.51]**
Heterogeneity: Not applicable								
Test for overall effect: Z = 0.70 (P = 0.48)								

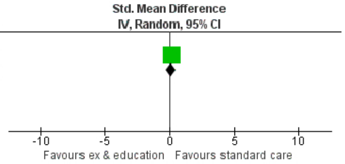

Test for subgroup differences: Not applicable

	Education			Standard care				Std. Mean Difference
Study or Subgroup	Mean	SD	Total	Mean	SD	Total	Weight	IV, Random, 95% CI
**4.1.3 Pain at 12 months (KOOS)**								
Culliton 2018 (education)	80.066	19.1	165	82.62	15.38	154	100.0%	-0.15 [-0.37, 0.07]
**Subtotal (95% CI)**			**165**			**154**	**100.0%**	**-0.15 [-0.37, 0.07]**

Heterogeneity: Not applicable

Test for overall effect: Z = 1.30 (P = 0.19)

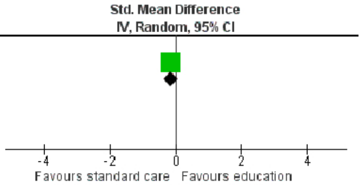

Test for subgroup differences: Not applicable

Appendix 1. Search strategy as applied in MEDLINE (tailored as relevant to other databases)

1 randomized controlled trial/ or randomized controlled trial.pt.

2 controlled clinical trial.pt.

3 randomized.ab.

4 placebo.ab.

5 randomly.ab

6 trial.ab

7 randomised.tw

8 1 or 2 or 3 or 4 or 5 or 6 or 7

9 review/

10 'systematic review$'.mp

11 9 or 10

12 8 or 11

13 Arthroplasty, Replacement, Knee/

14 Knee Prosthesis/

15 (arthoplast$ adj3 knee$).mp. [mp=title, abstract, original title, name of substance word, subject heading word, keyword heading word, protocol supplementary concept word, rare disease supplementary concept word, unique identifier]

16 (knee$ adj3 replac$).mp. [mp=title, abstract, original title, name of substance word, subject heading word, keyword heading word, protocol supplementary concept word, rare disease supplementary concept word, unique identifier]

17 (knee adj3 implant$).mp. [mp=title, abstract, original title, name of substance word, subject heading word, keyword heading word, protocol supplementary concept word, rare disease supplementary concept word, unique identifier]

18 13 or 14 or 15 or 16 or 17

19 12 and 18

For peer review only

BMJ Open

The effects of pre-surgical interventions on chronic pain after total knee replacement: A systematic review and meta-analysis of randomised controlled trials

Journal:	*BMJ Open*
Manuscript ID	bmjopen-2019-033248.R2
Article Type:	Original research
Date Submitted by the Author:	26-Nov-2019
Complete List of Authors:	Dennis, Jane; University of Bristol, Musculoskeletal Research Unit<br>Wylde, Vikki; University of Bristol, Musculoskeletal Research Unit<br>Gooberman-Hill, Rachael; University of Bristol, Bristol Medical School<br>Blom, AW; University of Bristol,<br>Beswick, Andrew; University of Bristol, Musculoskeletal Research Unit
**Primary Subject Heading**:	Surgery
Secondary Subject Heading:	Rehabilitation medicine
Keywords:	chronic post-surgical pain, prevention, systematic review, total knee replacement

SCHOLARONE™
Manuscripts

The effects of pre-surgical interventions on chronic pain after total knee replacement: A systematic review and meta-analysis of randomised controlled trials

Jane Dennis[1]

Vikki Wylde[1,2]

Rachael Gooberman-Hill[1,2]

Ashley W Blom[1,2]

Andrew D Beswick[1]

[1] Musculoskeletal Research Unit, Translational Health Sciences, Bristol Medical School, University of Bristol

[2] National Institute for Health Research Bristol Biomedical Research Centre, University Hospitals Bristol NHS Foundation Trust and University of Bristol

jane.dennis@bristol.ac.uk,v.wylde@bristol.ac.uk,r.gooberman-hill@bristol.ac.uk,ashley.blom@bristol.ac.uk,andy.beswick@bristol.ac.uk

Corresponding author: Jane Dennis

Word count: 5031

Musculoskeletal Research Unit
Translational Health Sciences
Bristol Medical School
University of Bristol
Learning and Research Building, Level 1
Southmead Hospital
Bristol
BS10 5NB

Keywords: chronic post-surgical pain; prevention; systematic review; total knee replacement

For peer review only - http://bmjopen.bmj.com/site/about/guidelines.xhtml

Abstract

Objective

Nearly 100,000 primary total knee replacements [TKR] are performed in the UK annually. The primary aim of TKR is pain relief, but 10-34% of patients report chronic pain. The aim of this systematic review was to evaluate the effectiveness of pre-surgical interventions in preventing chronic pain after TKR.

Design

MEDLINE, Embase, CINAHL, *The Cochrane Library* and PsycINFO were searched from inception to December 2018. Screening and data extraction were performed by two authors. Meta-analysis was conducted using a random effects model. Risk of bias was assessed using the Cochrane tool and quality of evidence assessed by GRADE.

Primary and secondary outcomes

Pain at six months or longer; adverse events.

Interventions

Presurgical interventions aimed at improving TKR outcomes.

Results

Eight RCTs with data from 960 participants were included. The studies involved nine eligible comparisons. We found moderate quality evidence of no effect of exercise programmes on chronic pain after TKR, based on a meta-analysis of 6 interventions with 229 participants (SMD 0.20, 95% CI -0.06 to 0.47, I^2 = 0%). Sensitivity analysis restricted to studies at overall low risk of bias confirmed findings. Another RCT of exercise with no data available for meta-analysis showed no benefit. Studies evaluating combined exercise and education intervention (n=1) and education alone (n=1) suggested similar findings. Adverse event data were reported by most studies, but events were too few to draw conclusions.

Conclusions

We found low to moderate quality evidence to suggest that neither pre-operative exercise, education nor a combination of both is effective in preventing chronic pain after TKR. This review also identified a lack of evaluations of other pre-operative interventions, such as multi-modal pain management, which may improve long-term pain outcomes after TKR.

PROSPERO REGISTRATION NUMBER: CRD42017041382.

For peer review only - http://bmjopen.bmj.com/site/about/guidelines.xhtml

Strengths and limitations of this study

- This systematic review evaluates recent evidence of the effectiveness of presurgical interventions in preventing chronic pain after total knee replacement
- Synthesis of adverse events data was not possible
- We only included studies that completed intervention delivery during the pre-operative period and did not include studies that evaluated interventions that began in the pre-operative period but extended into the peri-operative and post-operative period

For peer review only

For peer review only - http://bmjopen.bmj.com/site/about/guidelines.xhtml

Background

Osteoarthritis is the most common condition that affects the knee joint and causes considerable pain and disability. Effective treatments noted in guidelines include pain medications, exercise, and weight loss when appropriate[1,2]. If symptoms do not respond to pharmacological and conservative treatments, people may receive a total knee replacement (TKR). Annually in the UK, nearly 100,000 primary TKRs are performed[3,4] and in the USA in 2010, about five million people were estimated to be living with a TKR[5]. Outcomes are good for many, but a systematic review found that 10-34% of patients report unfavourable long-term pain outcomes at between three months and five years after TKR[6] which is associated with dissatisfaction with the outcome of surgery[7,8].

Some patient factors are associated with poor long-term pain outcomes. Using structural equation modelling, Sayers and colleagues demonstrated that pain during the pre-surgical period, particularly on movement, is strongly associated with chronic post-surgical pain, and a potentially important target for intervention[9]. Other pre-surgical risk factors for pain identified in multivariable analyses in representative populations include: poorer physical function, body mass index ≥35 kg/m^2 and poor general mental health[10]. With knowledge of risk factors for chronic pain, there is the potential for targeting care to those at high risk, or for risk factor modification[10,11]. While many pre-surgical interventions focus on preparing the patient for the procedure and hospital stay, reducing peri-operative pain and facilitating early mobilisation and recovery[10], their impact on the key outcome of chronic pain remains to be established. This is achievable through high quality, adequately powered randomised controlled trials with long-term follow up for patient related pain outcomes and ultimately through well-conducted systematic reviews with thorough consideration of sources of bias that may influence study results.

In a systematic literature search of MEDLINE, Embase, PsycINFO, CINAHL and The Cochrane Library on 18th December 2018, we identified 36 systematic reviews of pre-operative interventions in TKR. Twenty-five of these had searches conducted more than five years previously[10,12-35], and nine exclusively reported short-term outcomes (three months or less after surgery)[36-44]. Two more recent reviews considered the outcome of long-term pain[45,46] but neither had a registered protocol. Chesham and Shanmugam identified two randomised controlled trials in their search window (2004-2014) that reported no difference in pain at six months after surgery in patients randomised to exercise or control[45]. Wang and colleagues identified three randomised controlled trials of pre-operative exercise-based interventions in patients waiting for knee or hip replacement that reported long-term pain and

For peer review only - http://bmjopen.bmj.com/site/about/guidelines.xhtml

were published up to November 2015[46]. No difference in pain outcome was apparent at six months or longer in patients receiving intervention or control.

In this systematic review and meta-analysis, we assessed the effectiveness of pre-operative interventions in preventing chronic pain in patients receiving TKR. Previous reviews have largely focused on specific interventions but we have opted for a temporal framework and sought to include any intervention conducted before surgery. This review is part of a broad suite of reviews considering the timing of intervention throughout the TKR care trajectory[47,48].

Methods

The protocol for this systematic review was registered with PROSPERO (CRD42017041382)[49]. We formulated the research question according to the PICO principle[50] and used methods based on those recommended by Cochrane[51]. Reporting was in accordance with Preferred Reporting Items for Systematic Reviews and Meta-Analyses (PRISMA) guidelines[52] and a checklist is included in the Appendix.

Patient and public involvement (PPI)

Before starting the review, we held a meeting with stakeholders and patient representatives and discussed inclusion criteria and outcomes. Areas of potential relevance for pre-surgical intervention were highlighted, specifically exercise, education, psychological therapies, weight management, nutritional supplements, management of co-morbidities, and pain management with intra-articular injections. The importance of patient-reported pain as an outcome after TKR was emphasised. A musculoskeletal PPI forum has discussed the review and will advise on plain language summaries and dissemination[53].

Types of studies

To limit selection bias, we included only randomised controlled trials. Studies reported only as abstracts or unobtainable as full text copies were excluded. Language was not an exclusion criterion.

Participants

Eligible participants were adults on the waiting list for total knee replacement, up until the point of admission for surgery.

Interventions

Preventive interventions (pharmacological or non-pharmacological) delivered during the pre-surgical period were included. Studies where delivery of the intervention extended into the postoperative period were excluded.

Comparator group

For peer review only - http://bmjopen.bmj.com/site/about/guidelines.xhtml

Comparator group participants included those who had received usual care or an alternative intervention.

Outcomes

In preference, we considered patient-reported joint-specific pain intensity, typically measured using pain domains of the Oxford Knee Score (OKS)[54], Western Ontario and McMaster Universities Osteoarthritis Index (WOMAC)[55] or Knee injury and Osteoarthritis Outcome Score (KOOS)[56] at six months or longer after knee replacement. We selected these outcomes in preference to pain components of Knee Society's Clinical and Functional Scoring System (KSS)[57] and Hospital for Special Surgery Knee Score (HSS)[58] in which pain assessment is limited to one or two questions on intensity[59] and relies on clinician assessment which may not reflect the views of patients[60]. The choice of outcome timing reflects the time when, for many, pain levels have optimised[61]. If joint specific measures were not available, then we planned to use pain dimensions from quality of life measures including Short Form 36 Health Survey (SF-36)[62] and Short Form 12 Health Survey (SF-12)[63]. Had neither joint-specific nor generic pain outcomes been available, we planned to use data for pain measured using a visual analogue scale (VAS) or numerical rating scale (NRS). When no patient-reported outcome measure was reported, we used pain data from surgeon-assessed scores. When discrete pain data were unavailable, we report overall data that included those relating to pain, typically in combination with function and other factors.

Where data were available, we planned to report proportions of patients with a favourable pain outcome (for example "no worse than mild pain") as recommended in clinical trials[64] and systematic reviews[65].

Data on all adverse events were extracted and summarised narratively. Our focus was on adverse events related to the intervention, although we also report those related to surgery.

Study design

Randomised controlled trials of interventions commencing and completing within the pre-surgical period, with follow up at six months or longer after surgery for total knee replacement, were identified and assessed for eligibility. Authors reporting long-term outcomes of any type (typically, function only) were contacted to check whether pain outcome data were also available; those reporting aggregated data for hip replacements together with knee replacements were also contacted. Studies meeting these criteria and reporting pain data or composite scores including pain were included.

Database searches

For peer review only - http://bmjopen.bmj.com/site/about/guidelines.xhtml

We established a database of all randomised controlled trials in knee replacement, in preparation for a suite of systematic reviews in chronic pain after TKR[47,48]. Relevant trials were identified through searches (last updated on 18th December 2018) in: MEDLINE, Embase and PsycINFO on OvidSP; CINAHL on EBSCOhost; and *The Cochrane Library*. The search strategy as applied in MEDLINE is shown in Appendix 1. Within this resource, we searched for interventions conducted in the pre-surgical setting.

Citations and reference lists of key reviews and randomised trials were checked in ISI Web of Science. The ISRTN registry on BioMed Central was searched for ongoing randomised trials which were then checked for subsequent publication. No language restrictions were applied, and data from potentially relevant non-English language articles were extracted and translated to establish eligibility. Studies reported only as abstracts or that we were unable to acquire as full text copies using inter-library loans or author contact were excluded.

Study screening and data extraction

Records identified by searches were imported into Endnote X9 (Thomson Reuters). An initial screen for potential eligibility was undertaken by one review author (JD) with articles excluded only if clearly irrelevant. Typically, these were studies in osteoarthritis with no surgery, arthroscopy only, or surgery involving joints other than the knee. Subsequently, abstracts and full text articles were screened independently by two review authors. After consensus, reasons for exclusion were recorded.

Data from relevant randomised trials were extracted by one review author onto forms which were tested and refined before use. Microsoft Excel was used to record abbreviated information, specifically on: country where study was conducted; dates of recruitment; participant characteristics (indication, age, sex); inclusion and exclusion criteria; intervention and control content; setting, timing, duration and intensity of intervention; follow up data collection (including loss to follow up); instruments used to collect data on pain; outcome data including pain and adverse events. Data extracted were checked against source material by a second review author.

Group means and standard deviations of pain outcome measures were recorded at the longest follow up interval. Data with which to calculate proportions of patients with a poor outcome, however defined, were sought. Authors were contacted for unpublished data. Details of adverse events of any type were extracted.

Risk of bias within included studies

Potential sources of bias were assessed using the Cochrane risk of bias tool[66] by two review authors (JD and ADB) working independently; disagreements were adjudicated by a third

For peer review only - http://bmjopen.bmj.com/site/about/guidelines.xhtml

(VW). We present analyses where possible showing data from all relevant studies for a given outcome, but also conducted sensitivity analyses excluding studies assessed to be at overall high risk of bias.

Data analysis

When studies reported pain outcome data, these were presented and pooled where possible, using Review Manager 5.0[67]. As the control group in one study was shared by two active interventions, the sample size in meta-analysis was halved for the control group, to preserve independence of findings[51]. Results are presented for one comparison in the form of standardised mean differences (SMD), as instruments used to measure outcomes varied.

A random effects model was planned, due to anticipated heterogeneity as described below. A sensitivity analysis excluding those comparisons for which we had imputed data and/or studies we had assessed as being at overall high risk of bias was conducted. At protocol stage[49], we planned to generate funnel plots, if ten or more studies with similar interventions and controls were identified, as a method of estimating the risk of publication bias. In the event, data were not sufficient to do so.

Heterogeneity between studies (considering both magnitude and direction of effect) was assessed using visual inspection of the graph and the I^2-statistic. We anticipated sources of heterogeneity justifying subgroup analyses might include format and intensity of intervention; however, statistical heterogeneity proved to be unimportant and data were insufficient to conduct such analyses.

GRADE approach to evidence

We used the Grading of Recommendations Assessment, Development and Evaluation (GRADE) approach to assess the quality of the evidence[68], specifically study limitations, consistency of effect, imprecision, indirectness and publication bias.

Results

Conduct of the review is summarised in Figure 1. Searches identified 10,968 articles. Of these, 302 potentially relevant reports were considered in detail, of which 100 were identified as RCTs (some with multiple publications). Eighty-eight did not follow up participants to a minimum of six months. Four trials involving different interventions (smoking cessation[69], testosterone injections[70], epoetin-α injections[71], and bacterial screening[72] followed by decolonisation) did meet eligibility criteria for long term follow-up, but none of these included outcome data related to pain.

For peer review only - http://bmjopen.bmj.com/site/about/guidelines.xhtml

After detailed evaluation, ten articles reporting data from eight RCTs[73-80] were included in the review. These eight RCTs included data from 960 participants randomised to nine eligible comparisons. The interventions evaluated in the included studies were exercise, education, or a combination of both.

Seven publications assessed the impact of a single intervention (exercise (5), education (1), or a combination of both exercise and education (1)) against a no-treatment or attentional control. One publication reported relative effects of two different physical interventions (cardio-based exercise or physiotherapy) against a no treatment control[73]. We analysed data from the latter publication as two separate studies, dividing the control group during analysis so as to preserve independence of findings. Details of studies, including intervention characteristics, are shown in Table 1; risk of bias assessments in Figure 2; meta-analysis and sensitivity analyses in Figures 3 to 6. Finally, GRADE assessments of the quality of evidence for these classes of intervention for the outcomes of pain and serious adverse events are shown in Table 2.

Overview of study settings, sample sizes and interventions

Included studies were published between 1996 and 2018. Two studies were conducted in Canada[74,80], two in the USA[73,79], and one each in Belgium[77], Denmark[75], Switzerland[76] and Thailand[78]. Exercise interventions were delivered within clinics or within the home; education interventions were delivered within clinics in the studies in which it formed part of a composite intervention, or online when it was assessed in isolation.

Samples at recruitment ranged from 30 to 416 participants. The total number of participants involved in studies of exercise alone was 373; exercise and education together, 131; and education alone, 416.

Sample size calculations were reported by most investigators - but only one was based on the primary outcome used within this review (pain at six months). Other studies had as their main focus: satisfaction with TKR[80]; a short-term reduction of 10 points on the HSS and the reduction of hospital stay by one day[73]; the Chair Stand test[76]; a difference of 10 degrees in passive flexion[77]; an 8 point difference in WOMAC[79]; and a 10 point difference on the KOOS ADL subscale[75]. The variability in foci amongst studies makes it difficult for us to estimate a reasonable optimal information size for the review, particularly as some investigators revised their sample size estimates upwards as part of considering limitations of their studies[73,74].

Participants

Five studies exclusively recruited participants undergoing TKR for osteoarthritis[74,76-78,80]; one permitted those with rheumatoid arthritis[73]; and two included participants undergoing either

For peer review only - http://bmjopen.bmj.com/site/about/guidelines.xhtml

hip or knee replacement for osteoarthritis, with long-term data reported separately[79] or in aggregate[75]. Mean age of participants ranged from 63 to 70 across studies. Gender, where reported, varied greatly, with studies recruiting 17%[78] to 60% women[73].

Interventions

Seven interventions assessed within six studies[73,75-79] were exercise-based; one was multifactorial with exercise and an education component focusing on walking with crutches, bed mobility and postoperative range of movement[74]; and one featured an online educational component alone[80].

All exercise interventions included strengthening[73-79], with additional components targeting gait re-education[74], functional exercise[74,76], improving knee range of movement, flexibility or mobility[73,74,76,77,79] and cardiovascular conditioning[73,76,79]. Two studies evaluated a particular manualised programme, 'NEuroMuscular Exercise training program for patients with knee or hip osteoarthritis assigned for total joint replacement' (NEMEX-TJR)[81] which is described as a series of neuromuscular exercises combined with biomechanical training. One study included a series of pool-based exercises as well as standard indoor exercises[79]. The majority of exercise-based interventions required participants to travel to clinics or community-based exercise centres but two[77,78] involved exclusively home-based exercise. Staff delivering interventions were, in the majority of cases, physiotherapists; exceptions were one trial in which the intervention was delivered by an exercise physiologist[73] and one in which it was unclear who delivered the intervention[77].

Participants in the study evaluating both exercise and education received a single session of education (reinforced by provision of a written materials) concerning crutch walking on level ground and stairs, bed mobility and transfers, and post-operative range of motion routines[74]. Those in the study focussing solely on education[80] received a 31 page guide as well as access to an online learning tool during their preadmission consultation, followed by subsequent reminders of their access. The customised e-learning tool included 32 brief (1-2 minute) professionally filmed educational videos presented by surgeons, therapists, or previous recipients of TKRs, in which expectations related to pain, functional outcomes, limitations, and restrictions were addressed, as well as animations allowing them to visualise the surgery itself.

Intensity, duration, measures of adherence / compliance

Exercise interventions were delivered for between 3 and 12 weeks, 2-7 times a week. The shortest duration of intervention was a three-week home-based intervention[78] which featured the most intense dose (3 sessions per day, 7 days a week). The longest period of intervention (8-12 weeks) took place in the two trials[75,76] delivering the clinic-based NEMEX-

For peer review only - http://bmjopen.bmj.com/site/about/guidelines.xhtml

TJR intervention[81]. Here, variability in duration could be determined by how close to the surgery a patient was at time of recruitment.

With regard to exercise interventions conducted within clinics or at home, investigators typically assessed adherence via logbooks[74-77] recording attendance and sometimes details of achievement and effort. One study of a home-based intervention without formal reporting of adherence featured daily telephone calls to participants[78]; two clinic-based interventions[73] reported that all participants completed all sessions. The study focussing solely on education[80] assessed the number of overall 'hits' to different educational videos concerning different aspects of the TKR pathway.

Comparator groups

Amongst the trials assessing the effects of exercise or exercise combined with education, detail and nature of comparator groups varied. Five trials described comparator groups as 'usual care'. For three trials the descriptions of usual care were brief but indicated that participants continued with regular activities until surgery[74,77,78]. In one trial of cardiovascular exercise, usual care was described as participants having a single meeting with a physiotherapist before their operation for 45 minutes and being provided with printed material about post-operative exercise regimens[73]. In another trial, usual care involved all participants being offered a "standard preoperative educational package" comprising written information on the operation, 'expected postoperative progression', and recommended exercises, and a 3-hour in-clinic information session led by health professionals one week prior to surgery[75].

In the two other studies assessing exercise, the comparator groups were designed as attentional controls. In one, participants were sent information on joint replacement, recommended home modifications, and on preparing for surgery, reinforced by three telephone calls[79]. The most intensive control condition was 'knee school' offered alongside an exercise intervention but also offered to the control group participants[76]. The knee school included formal teaching and peer discussions, was led by an experienced and specially-trained physiotherapist over three weekly individual or group sessions. Participants were provided with information on anatomy (including photos, diagrams and models), recommended activities after surgery, post-operative pain management, and rehabilitation.

In the trial evaluating online education, the control group were provided with a 31 page copy of 'My Guide to Total Knee Joint Replacement'[80].

Outcomes and outcome data

For peer review only - http://bmjopen.bmj.com/site/about/guidelines.xhtml

Six studies reported a joint- or condition- specific patient reported pain outcome measure[74-76,78-80]. In two publications (one reporting data for two separate comparisons), the outcomes reported were surgeon-assessed[73,77].

The latest pain assessment was reported at about six months[73,74,76,77] or 12 months after surgery[78-80]. Measures used were WOMAC pain[74,78], KOOS pain[76,80], SF-36 bodily pain[74,76,80], VAS pain[78], HSS pain score[73], and KSS[77].

We made robust attempts to obtain data through contact with investigators from studies in which data were provided within graphs without standard deviations[73,76], in tables with means but without standard deviations[80], as 'non significant' only[77], or as part of aggregated hip and knee data[75]. In three studies, investigators generously provided relevant data in the form of group means and SDs and these data were used[75,76,80]. Following information that data for one study had been permanently lost[73], we measured WOMAC pain data from a graph and, in the absence of standard deviations, used values from a cohort of patients with the same outcome measure reported after total knee replacement but converted from a 50 point to a 30 point scale[82]. The first author of an exercise-based study which had reported pain findings as simply 'n.s.' (non-significant), responded to our enquiry to indicate that they could no longer access study data.

Risk of bias

Four studies were assessed as being at an overall low risk of bias[74,76,77]. In other studies, we had concerns about potential biases due to differences in baseline patient characteristics in two comparisons[73] and in one study each, large losses to follow up[79], and issues regarding sequence generation[78].

Effects of intervention: exercise alone

<u>Pain at six months or longer (Figure 3)</u>

Combined results of six eligible interventions reported in 5 studies[73,75,76,78,80] indicate no clear effect of intervention on long-term pain (SMD 0.20, 95% CI -0.06 to 0.47; participants = 229). There was no heterogeneity across studies, $Chi^2 = 3.39$, $I^2 = 0\%$.

In a sensitivity analysis restricted to two studies with low risk of bias[74,76], group results provided even less suggestion of difference between groups (SMD 0.08, 95% CI -0.29 to 0.45; participants = 110; $I^2 = 0\%$) (see Figure 4).

Results for one study (n = 122) within this category for which no data were suitable for meta-analysis are in line with the results above, with 'non significant'[77] findings.

Effects of intervention: exercise combined with education

For peer review only - http://bmjopen.bmj.com/site/about/guidelines.xhtml

Pain at 6 months or longer (Figure 5)

Results of a single study of exercise with the additional component of one session of education (n = 109 followed up) suggest no clear difference between intervention and standard care on long-term pain as assessed by the WOMAC pain scale (MD 2.00, 95% CI -3.45 to 7.45)[74].

Effects of intervention: education alone

Pain at 6 months or longer (Figure 6)

Results of a single study of education provided by an e-learning tool (n = 319) suggest no clear difference between intervention and standard care in terms of long-term pain as assessed on the KOOS (MD -2.55, 95% CI -6.35 to 1.24)[80].

Adverse events

Peri and post-surgical complications including infections were assessed in most studies; see Table 1. Where reported, these do not appear to have differed between groups. Two studies reported pre-surgical events we judged likely to be intervention-related[76,77]; in each case, participants in an exercise intervention group reported increased pain. No data for this outcome were reported in the study focussing on education alone[80].

Discussion

Summary of main findings

In people with osteoarthritis receiving TKR, the potential value of interventions to manage pre-operative risk factors or to target care has frequently been assessed, most often focussing on interventions this review has identified (exercise or 'prehabilitation' and/or education with the aim of managing expectations). We found low to moderate quality evidence for no effect on our primary outcome (pain assessed at six months or longer) of either exercise, education or education combined with exercise, and we identified no eligible studies assessing the effects of any other eligible pre-operative interventions (e.g. weight loss programmes or smoking cessation).

Strengths and limitations of this study

With broad inclusion criteria we aimed to identify diverse interventions conducted in the pre-operative period. Important issues that our review benefits from are preregistration of the study protocol to limit reporting bias, conduct of study according to PRISMA guidelines and comprehensive up to date searches of a range of appropriate literature databases. Experienced systematic reviewers undertook screening of articles, data extraction and risk of bias assessment in duplicate. Author contact was extensive and with the help of study

For peer review only - http://bmjopen.bmj.com/site/about/guidelines.xhtml

investigators, we included data from studies which had been excluded from previous meta-analyses or had been included with estimates for variances. Furthermore, use of GRADE methodology allowed us to assess the overall quality of the evidence.

A limitation of our review concerns our focus on prevention of long-term pain only. This decision was based on guidelines which emphasise that osteoarthritic pain which is not controlled by conservative treatments is a primary reason that people undergo TKR[83]. Prevention of long-term pain is a key determinant of patient satisfaction with TKR[84]. Although the primary outcome of many studies we identified was function, pain severity was an important secondary outcome. We cannot draw conclusions on the value of the interventions we have examined from the point of view of (for example) the benefit to individuals or society of quicker short term recovery, as proposed by some triallists[75].

The secondary outcome of this review was adverse events, but reporting was lacking. The poor quality of adverse event reporting in surgical trials is recognised[85], and standardisation is required to improve quality and reduce heterogeneity, particularly in orthopaedics. Although patient reported outcomes are frequently considered to be at high risk of bias, we considered them to be appropriate in this review where questionnaires were completed over six months after a presurgical intervention.

We also excluded studies in which the intervention continued beyond the period of the TKR procedure; a separate review considering studies including interventions which combined prehabilitation and post-TKR rehabilitation may well be justified.

This review in context

This is the first review in nearly a decade[35] which takes as its focus the preoperative period itself, rather than aiming at a class of interventions; the evidence base, however, remains limited.

Previous reviews have focused on outcomes relating to pre-operative function and pain, the in-hospital experience, and long-term recovery[10,12-46]. Review conclusions vary based on the outcomes and follow up times. In general, those concentrating on short term function and outcomes related to discharge are more likely to report early patient benefit and shorter hospital stay for patients receiving pre-surgical exercise and education compared with controls. Those considering longer-term post-operative outcomes show little difference in outcomes between randomised groups. One review which evaluated the effects of 'prehabilitation' across *all* surgical patients concluded that "*prehabilitation studies are not recommended in patients with osteoarthritis for whom arthroplasty is planned*"[14]. Subsequently, amongst the most comprehensive of systematic reviews conducted in arthroplasty alone is that by Wang and colleagues[46]. They concluded that the effects of

For peer review only - http://bmjopen.bmj.com/site/about/guidelines.xhtml

prehabilitation on long-term and indeed many short-term outcomes were too small to be considered clinically important.

Our review adds to the existing evidence base as it includes two trials not included in other reviews[76,80] and previously unreported data from an older trial[75] which was acquired from study authors. Inclusion of data from these three trials in our meta-analysis did not support use of pre-operative exercise or education interventions for the prevention of chronic pain but evidence supporting this was of low to moderate quality. This is in line with previous review conclusions[45,46]. Furthermore, a range of pre-operative interventions have not been evaluated in randomised trials with long-term follow up for pain outcome.

Implications for practice and research

There are several possible explanations for the observed lack of a convincing effect of pre-operative interventions on long-term pain after TKR reported within this review. All pre-operative interventions conducted within an undifferentiated general population may appear ineffective in the long-term, as for many patients, TKR surgery is highly effective in reducing long-term pain. To detect an effect of intervention for the small but important minority of patients for whom TKR is not highly effective in reducing long-term pain, studies may need either to be much larger, or more focussed on particular patient subgroups. Despite the fact that patients report that appropriate and realistic support before surgery can help them achieve a long-term positive experience of surgery[86], it is acknowledged that it is difficult to recruit patients to randomised controlled trials before surgery and participants in existing trials may have been highly selected. Patients may have exhausted conservative strategies before surgery and, in evaluating new approaches, there may be an unwillingness among patients to participate in randomised trials[10]. Intervention content may have focused on improving pre-operative physical function and preparing patients for their hospital admission and recovery without specific reference or ‘flags’ to help identify participants whose recovery did not follow the norm.

An aspect of pre-surgical health with a possible link with chronic pain is psychological distress[87]. As an example, pain catastrophizing has been implicated as a risk factor for chronic pain[88]. However, a recent trial where intervention began in the pre-surgical period and extended well beyond it found that pain coping skills training for people with high pain catastrophizing did not improve WOMAC pain scores at 12 months after TKR[89]. Other aspects of preoperative psychological distress are associated with long-term pain after TKR and evaluation of treatment strategies merits investigation[87]. For other pre-operative strategies, such as smoking cessation, weight loss and comorbidity management, there may be tenuous, if any, mechanisms to link with long-term pain outcomes. However, they share a

For peer review only - http://bmjopen.bmj.com/site/about/guidelines.xhtml

common aim in preparing patients for surgery and preventing adverse events that may limit the potential benefit of knee replacement, so we believe it right to advocate that studies investigating their effects incorporate long term follow-up.

Conclusion

Pre-surgical interventions have been evaluated in patients receiving TKR. We found low to moderate quality evidence to suggest that exercise is not effective in preventing long-term pain in adults scheduled to receive total knee replacement and it is difficult to recommend further research except of novel approaches and/or in specific populations. The evidence base for education and exercise combined with education was highly limited. However, studies of pre-operative interventions with long-term follow up after surgery are feasible and adequately powered, randomised controlled trials, planned and reported according to CONSORT standards, should be conducted to identify clinically effective pre-operative treatments to help patients achieve a long-term pain-free outcome after knee replacement.

Acknowledgements

We gratefully acknowledge unpublished outcome data from the following investigators: Dr Sharon Culliton, Dr Erika Huber and Dr Allan Villadsen. We also are thankful for confirmation concerning other study data from Professor Darryl D'Lima and Dr Fabrizio Matassi. We are also thankful for translation of Yunfei Li, University of Bristol, for his help in translating a paper from Chinese.

Author contributions

All authors contributed to the concept and design of the study. JD, ADB and VW contributed to the acquisition and analysis of data. JD and ADB drafted the article and VW, RG-H and AWB revised it critically for important intellectual content. JD and ADB take responsibility for the integrity of the work as a whole, from inception to finished article.

Funding

This study is funded by the National Institute for Health Research (NIHR) [NIHR Programme Grant for Applied Research (Grant Reference Number RP-PG-0613-20001)]. This study was also supported by the NIHR Biomedical Research Centre at the University Hospitals Bristol NHS Foundation Trust and the University of Bristol. The views expressed in this publication are those of the authors and not necessarily those of the NHS, the National Institute for Health Research or the Department of Health and Social Care.

Disclaimer

For peer review only - http://bmjopen.bmj.com/site/about/guidelines.xhtml

For peer review only

The views expressed are those of the authors and not necessarily those of the NHS, the NIHR or the Department of Health. The funder had no involvement in the study design, data collection, data analysis, interpretation of data or writing of the manuscript.

Competing interests

No author has any direct conflict of interest with the published work above. Outside of this work, VW and AWB are co-applicants on an institutional grant from Stryker for a study evaluating the outcomes of the Triathlon knee replacement.

Patient consent

Not required.

Data sharing statement

No additional data available

Legend

Figure 1. PRISMA_Flow_Diagram

Figure 2. Risk of bias assessments

Figure 3. Forest plot: exercise

Figure 4. Forest plot: exercise sensitivity analysis

Figure 5. Forest plot: exercise & education

Figure 6 Forest plot: education alone

For peer review only

Table 1. Characteristics of studies evaluating pre-operative exercise and education

Publication<br>Location<br>Date of study	Indication<br>Number randomised (intervention : control)<br>Mean age (%female)	Primary focus of intervention	Study setting<br>Timing, duration and intensity	Control group care	Longest follow up after surgery<br>Losses to follow up (intervention: control)<br>Risk of bias	Outcomes
Beaupre et al, 2004<br>Canada<br>Date not specified	Non-inflammatory arthritis<br>N= 131<br>(65:66)<br>67 years (55%)	Multifactorial: exercise (gait re-education, functional, ROM, strengthening) and education on crutch walking, bed mobility and postoperative ROM	Community physical therapy clinic group<br>3-times per week for 4-weeks, 6 weeks before surgery	No intervention	6 and 12 months<br>16 (10:6) patients cancelled surgery, 6 (4:2) patients lost to 12 month follow up<br>Low overall risk of bias	No difference in WOMAC pain or SF-36 bodily pain at 6 and 12 months (p>0.05)<br>General complications, "similarly dispersed across both groups": Pulmonary embolism (2:2), DVT (3:6), Superficial infection (2:3), Deep infection (1:0), Manipulation (1:2)
Culliton et al. 2018<br>Canada<br>2013-2014	Osteoarthritis<br>N= 416 (207:209)<br>64 years (43%)	Education: access to an online e-learning tool during pre-admission visit and 31-page guide also provided to the control group. Tool consisted of custom-made 32 videos (1-2 minutes) addressing patient expectations, presented by surgeons, physical therapists and patients	Access to e-learning tool remained active until the study end (1 year). Tool consisted of custom-made 32 videos (1-2 mins) addressing patient expectations, presented by surgeons, physical therapists and patients	Booklet alone	12 months post-operative<br>Data analysed for 76.6 % of original sample randomised. Reasons include TKR not taking place or lack of email address. Later exclusions due to failure to submit data.<br>Low overall risk of bias	1 year postoperative, significant between-group differences in favour of the control group for the KOOS symptoms score (p= 0.04). Pain scores obtained from authors directly were not significantly different between groups.<br>Adverse events not reported.

For peer review only - http://bmjopen.bmj.com/site/about/guidelines.xhtml

D'Lima et al. 1996<br>Cardiovascular conditioning<br>USA<br>Date not specified	Rheumatoid arthritis or osteoarthritis<br>N=20 (10:10)<br>70.6 years (35%)	Exercise (stretching, strengthening, cardiovascular conditioning)	Hospital. Individualised<br>45 minute sessions, 3 times a week for 18 weeks commencing 6 weeks before surgery	One meeting with physical therapist	24 and 48 weeks<br>No losses to follow up reported<br>High risk of bias: small numbers of patients; baseline demographic differences; failure to report data other than graphically	Hospital for Special Surgery Knee HSS pain score improved in intervention group compared with control but not statistically significant<br>Sleep apnoea (1:0), Manipulation (1:0), Deep infection (0:1), Intestinal pseudo-obstruction (0:1)
D'Lima et al. 1996<br>Physical therapy<br>USA<br>Date not specified	Rheumatoid arthritis or osteoarthritis<br>N=20 (10:10)<br>69.0 years (60%)	Exercise (strengthening, range of motion)	Hospital. 1 on 1 programme<br>45 minute sessions, 3 times a week for 18 weeks commencing 6 weeks before surgery	One meeting with physical therapist	24 and 48 weeks<br>No losses to follow up reported<br>High risk of bias: small numbers of patients; baseline demographic differences	Hospital for Special Surgery Knee Rating pain score improved in intervention group compared with control but not statistically significant<br>Atrial fibrillation (1:0), Paroxysmal tachycardia (1:0), Deep infection (0:1), Intestinal pseudo-obstruction (0:1)
Fernandes et al. 2017<br>Denmark<br>2010-2011<br>1 hospital	Osteoarthritis<br>165 (84; 81) hip or knee replacement<br>Mean 67.9 (SD 8.6); 66.9 (8.3)<br>(56%)	Exercise (neuromuscular focusing on lower extremity muscular control and quality of movement)	Hospital: group-based, physiotherapist led<br>Mean 13.1 one-hour sessions twice per week during 8 weeks before surgery.	Standard care	61 weeks<br>2; 5<br>Low risk of bias	Mean differences at 61 weeks favoured the exercise group for all KOOS subscales, but only significantly for quality of life subscale. Disaggregated long-term data for knee replacement acquired by personal contact<br>0; 2 deep infection
Huber et al. 2015<br>Switzerland<br>2009-2012	Osteoarthritis<br>N=45 (22:23)<br>70.4 years (47%)	Exercise (neuromuscular including functional and strengthening)	Hospital: group based<br>8 or more sessions commencing 6-12 weeks before surgery.	All patients attended "knee school" on 3 occasions from about 4 weeks	12 months<br>9 (5:4) lost to follow up<br>Low risk of bias	No difference in KOOS pain or SF-36 bodily pain between groups at 12 months<br>1 intervention patient had a joint specific adverse event

For peer review only - http://bmjopen.bmj.com/site/about/guidelines.xhtml

For peer review only

				before surgery		
Matassi et al. 2014<br>Belgium<br>2005-2006	Osteoarthritis<br>N=122 (61:61)<br>66.5 years (48%)	Exercise (muscle strength and flexibility)	Home<br>5 days per week for 6 weeks commencing 6 weeks before surgery	Maintained regular activities	1 year<br>No losses to follow up reported<br>High risk of bias due to selective outcome reporting; outcome also not patient reported	No significant effect of exercise on the Knee Society Clinical Rating System knee score or patient function score<br>Manipulation (5:3). In the intervention group 1 patient developed ipsilateral adductor tendinitis and 1 patient stopped exercises due to increased knee pain
Rooks et al. 2006<br>USA<br>2001-2003	Osteoarthritis<br>N=45 (22:23)<br>67.0 years (53%)	Exercise (strengthening, flexibility, cardiovascular, pool exercises)	Community fitness facility<br>3 times per week for 6 weeks 30-60 mins each increasing intensity from 4-6 weeks	2 education mailings and 3 telephone calls	26 weeks<br>16 (8:8) lost to follow up<br>High risk of bias: large loss to follow up	Statistically significant greater improvement in SF-36 bodily pain at 26 weeks ($p<0.05$) but no difference for WOMAC pain<br>Perioperative complications (3:4). No serious adverse events in either group requiring hospital admission
Tungtrongjit et al. 2012<br>Thailand<br>2011-2012	Osteoarthritis<br>N=68 (30:30 in analyses)<br>64.5 years (17%)	Exercise (quadriceps strengthening)	Home<br>3 times per day for 3 weeks commencing 3 weeks before surgery	Asked to continue normal activities	6 months<br>8 patients excluded from analyses due to adverse events and loss to follow up<br>Possible risk of bias: unclear sequence generation; data provided only on patients followed up	WOMAC pain improved in intervention compared with controls ($p= 0.029$) but not VAS pain ($p=0.137$)<br>Across all randomised patients there was 1 surgical wound infection, 1 post-operative knee trauma, and 2 cases of post-operative wound dehiscence

WOMAC Western Ontario and McMaster Universities Osteoarthritis Index; SF-36 Short Form 36; VAS Visual analogue scale; KOOS Knee disability and Osteoarthritis Outcome Score; EQ-5D European Quality of Life-5 Dimensions

For peer review only - http://bmjopen.bmj.com/site/about/guidelines.xhtml

Table 2

Summary of findings: Exercise alone compared to standard care for knee replacement (long-term outcome only)

Patient or population: Adult patients scheduled for total knee replacement
Intervention: 3- 12 weeks exercise conducted in the presurgical period
Setting: Clinic-supervised exercise (5 studies); home-based exercise (2 studies)
Comparison: Standard care

Outcome № of participants (studies)	Relative effect (95%CI)	Illustrative comparative risks (95% CI)		Certainty	Comments
**Long-term pain**<br>Assessed with: Various scales (WOMAC[a], KOOS[b], HSSK[c])<br>Follow up: range 6 months to 12 months<br>№ of participants in metaanalysis: 229 (6 RCTs)<br>№ of participants not in metaanalysis: 122 (1 RCT).	**Metaanalysis**:<br>SMD **0.2 higher** (-0.06 - 0.47)<br>**Narrative**<br>"Non significant difference"	Mean KOOS scores in the untreated group ranged from 73.2 to 85.95 in the control group; mean HSSK scores in the control group were 27; mean WOMAC scores in control group were<br>-2.3 to -4.8	The mean level of pain after exercise was 0.20 standard deviations lower (-0.06 to 0.47 lower).	⊕⊕⊕◯<br>MODERATE [d,e]	There was no clear evidence of a difference between exercise and standard care for long-term pain either from data from the metaanalysis or from a comparatively large study not included within the metaanalysis.<br>We rescaled the SMD finding to a commonly used instrument in the field - the KOOS, using estimations based on the mean of a representative study within the analysis (Huber 2015) and our pooled SMD of 0.02 (-0.06 to 0.47 higher). This indicates that exercise led to a mean increase of 2.7 units/points on the KOOS (95% CI -0.8 to 6.29) in the treated group.<br>Sensitivity analysis of two studies at low risk of bias suggested a smaller effect (mean increase of 1.46 points on the KOOS (95% CI-4.58 to 7.50)
**Adverse events**	See comment	See comment	See comment	⊕⊕◯◯<br>LOW [d,f]	Two studies reported treatment-related adverse events (increase in pain) separately from the main pain outcomes, which occurred during the period of intervention. Information on perioperative complications e.g. superficial and deep infections, were reported in all studies. Events were few and attribution to treatment status difficult

CI: Confidence interval; **MD:** Mean difference; **SMD:** Standardised mean difference

For peer review only - http://bmjopen.bmj.com/site/about/guidelines.xhtml

Summary of findings: Exercise alone compared to standard care for knee replacement (long-term outcome only)

Patient or population: Adult patients scheduled for total knee replacement
Intervention: 3- 12 weeks exercise conducted in the presurgical period
Setting: Clinic-supervised exercise (5 studies); home-based exercise (2 studies)
Comparison: Standard care

Outcome<br>№ of participants<br>(studies)	Relative effect (95%CI)	Illustrative comparative risks (95% CI)		Certainty	Comments

GRADE Working Group grades of evidence
High certainty: We are very confident that the true effect lies close to that of the estimate of the effect
Moderate certainty: We are moderately confident in the effect estimate: The true effect is likely to be close to the estimate of the effect, but there is a possibility that it is substantially different
Low certainty: Our confidence in the effect estimate is limited: The true effect may be substantially different from the estimate of the effect
Very low certainty: We have very little confidence in the effect estimate: The true effect is likely to be substantially different from the estimate of effect

Explanations

a. WOMAC = The Western Ontario and McMaster Universities Osteoarthritis Index (WOMAC) (Bellamy et al 1988). The original pain scale runs from 0 to 20 and a higher number indicates a greater pain. There are several modifications. Some triallists also reverse the polarity and/or transform responses into a 0-100 scale where a high number indicates less pain. We standardised to this latter method as it was most common and was similar to the KOOS and the HSSK.
b. KOOS = Knee Injury and Osteoarthritis Outcome Score (Roos et al 1998) . The scale ranges from 0 – 100 and a higher number indicates less pain
c. HSSK = Hospital Society Score (Insall et al 1976). Pain is measured on a scale of 0 to 30, with a high number indicating less pain.
d. Downgraded due to risk of bias associated with lack of blinding of both personnel and participants in all studies also concerns about sequence generation /allocation concealment in three studies.
e. We did not downgrade for imprecision as findings of the one large study not included in metaanalysis were very similar to the six studies included in metaanalysis
f. We downgraded the quality of evidence for study limitations, in particular imprecision: estimate based on few events

For peer review only - http://bmjopen.bmj.com/site/about/guidelines.xhtml

For peer review only

Summary of findings: Exercise & education compared to standard care for knee replacement (long-term outcome only)

Patient or population: Adult patients scheduled for total knee replacement
Intervention: 1 session of education combined with 4 weeks' exercise in the pre-operative period
Setting: Clinic-supervised exercise Clinics (1 study)
Comparison: TAU

Outcome № of participants (studies)	Relative effect (95%CI)	Illustrative comparative risks (95% CI)		Certainty	Comments
**Long-term pain** Assessed with: WOMAC[a] scale Follow up: 12 months № of participants: 109 (1 RCT)	**Single study results**: MD **2 units higher** (3.45 lower to 7.45 higher) -	Mean score of untreated control group was 80 on the WOMAC (sd = 16)	Exercise and education resulted in a mean score of 82 on the WOMAC (sd= 13)	⊕⊕◯◯ LOW [b,c,d]	)
**Adverse events**	See comment	See comment	See comment	⊕⊕⊕◯ VERY LOW [b,c,d,e]	General complications, "similarly dispersed across both groups": Pulmonary embolism (2:2), DVT (3:6), Superficial infection (2:3), Deep infection (1:0), Manipulation (1:2)

CI: Confidence interval; **MD:** Mean difference

GRADE Working Group grades of evidence
High certainty: We are very confident that the true effect lies close to that of the estimate of the effect
Moderate certainty: We are moderately confident in the effect estimate: The true effect is likely to be close to the estimate of the effect, but there is a possibility that it is substantially different
Low certainty: Our confidence in the effect estimate is limited: The true effect may be substantially different from the estimate of the effect
Very low certainty: We have very little confidence in the effect estimate: The true effect is likely to be substantially different from the estimate of effect

Explanations

a. WOMAC = The Western Ontario and McMaster Universities Osteoarthritis Index (WOMAC) (Bellamy et al 1988). The original pain scale runs from 0 to 20 and a higher number indicates a greater pain. There are several modifications. Some triallists also reverse the polarity and/or transform responses into a 0-100 scale where a high number indicates less pain. We standardised to this latter method as it was most common and was similar to the KOOS and the HSSK.
b. Downgraded due to risk of bias due to lack of blinding of participants or personnel.
c. Downgraded for imprecision due to being one study with less than an optimal information sample size
d. There were too few studies to reliably assess risk of publication bias or other sources of small study bias
e. We downgraded the quality of evidence for study limitations, in particular imprecision: estimate based on few events

For peer review only - http://bmjopen.bmj.com/site/about/guidelines.xhtml

Summary of findings: Exercise & education compared to standard care for knee replacement (long-term outcome only)

Patient or population: Adult patients scheduled for total knee replacement **Setting**: Clinic-supervised exercise Clinics (1 study)
Intervention: 1 session of education combined with 4 weeks' exercise in the pre-operative period **Comparison**: TAU

Outcome № of participants (studies)	Relative effect (95%CI)	Illustrative comparative risks (95% CI)		Certainty	Comments

Summary of findings: Education compared to standard care for knee replacement (long term outcome only)

Patient or population: Adult patients scheduled for total knee replacement **Setting**: Clinic followed by online access (1 study)
Intervention: Access to online elearning tool with active demonstration at presurgical visit followed by email prompts **Comparison**: TAU

Outcome № of participants (studies)	Relative effect (95%CI)	Illustrative comparative risks (95% CI)		Certainty	Comments
**Longterm pain**<br>Assessed with: KOOS scale[a]<br>Follow up: 12 months<br>№ of participants: 319<br>(1 RCT)	**Single study results**:<br>MD **2.55 lower**<br>(6.35 lower to 1.24 higher)	Mean score of untreated control group was 82.62 on the KOOS (sd = 15.4)	Exercise and education resulted in a decrease on the KOOS, leading to a mean score in the treated group of 80.01(sd= 19)	⊕⊕⊕◯<br>MODERATE [b,c]	
**Adverse events**	See comment	See comment	See comment		No data on adverse events appear to have been sought within the one study within this comparison

CI: Confidence interval; **MD:** Mean difference

GRADE Working Group grades of evidence
High certainty: We are very confident that the true effect lies close to that of the estimate of the effect
Moderate certainty: We are moderately confident in the effect estimate: The true effect is likely to be close to the estimate of the effect, but there is a possibility that it is substantially different
Low certainty: Our confidence in the effect estimate is limited: The true effect may be substantially different from the estimate of the effect
Very low certainty: We have very little confidence in the effect estimate: The true effect is likely to be substantially different from the estimate of effect

For peer review only - http://bmjopen.bmj.com/site/about/guidelines.xhtml

Summary of findings: Exercise & education compared to standard care for knee replacement (long-term outcome only)

Patient or population: Adult patients scheduled for total knee replacement
Intervention: 1 session of education combined with 4 weeks' exercise in the pre-operative period
Setting: Clinic-supervised exercise Clinics (1 study)
Comparison: TAU

Outcome<br>№ of participants<br>(studies)	Relative effect (95%CI)	Illustrative comparative risks (95% CI)		Certainty	Comments

Explanations

a. KOOS = Knee Injury and Osteoarthritis Outcome Score (Roos et al 1998) . The scale ranges from 0 – 100 and a higher number indicates less pain
b. Downgraded due to risk of bias due to lack of blinding of participants or personnel and high loss to followup (24%)
c. There were too few studies to reliably assess risk of publication bias or other sources of small study bias

For peer review only - http://bmjopen.bmj.com/site/about/guidelines.xhtml

References

1. McAlindon TE, Bannuru RR, Sullivan MC, Arden NK, Berenbaum F, Bierma-Zeinstra SM, Hawker GA, Henrotin Y, Hunter DJ, Kawaguchi H, Kwoh K, Lohmander S, Rannou F, Roos EM, Underwood M. OARSI guidelines for the non-surgical management of knee osteoarthritis. Osteoarthritis Cart 2014;22(3):363-88.
2. Osteoarthritis: care and management. London: National Institute for Health and Clinical Excellence; 2014.
3. National Joint Registry for England, Wales, Northern Ireland and the Isle of Man: 15th Annual Report. Hemel Hempstead: NJR Centre 2018.
4. Scottish Arthroplasty Project. Annual report 2018. Edinburgh: NHS National Services Scotland; 2018.
5. Maradit Kremers H, Larson DR, Crowson CS, Kremers WK, Washington RE, Steiner CA, Jiranek WA, Berry DJ. Prevalence of total hip and knee replacement in the United States. J Bone Joint Surg (Am) 2015;97(17):1386-97.
6. Beswick AD, Wylde V, Gooberman-Hill R, Blom A, Dieppe P. What proportion of patients report long-term pain after total hip or knee replacement for osteoarthritis? A systematic review of prospective studies in unselected patients. BMJ Open 2012;2(1):e000435.
7. Nilsdotter AK, Toksvig-Larsen S, Roos EM. Knee arthroplasty: are patients' expectations fulfilled? A prospective study of pain and function in 102 patients with 5-year follow-up. Acta Orthop 2009;80(1):55-61.
8. Scott CE, Howie CR, MacDonald D, Biant LC. Predicting dissatisfaction following total knee replacement: a prospective study of 1217 patients. J Bone Joint Surg (Br) 2010;92(9):1253-8.
9. Sayers A, Wylde V, Lenguerrand E, Beswick AD, Gooberman-Hill R, Pyke M, Dieppe P, Blom AW. Rest pain and movement-evoked pain as unique constructs in hip and knee replacements. Arthritis Care Res 2016;68(2):237-45.
10. Blom AW, Artz N, Beswick AD, Burston A, Dieppe P, Elvers KT, Gooberman-Hill R, Horwood J, Jepson P, Johnson E, Lenguerrand E, Marques E, Noble S, Pyke M, Sackley C, Sands G, Sayers A, Wells V, Wylde V. Improving patients' experience and outcome of total joint replacement: The RESTORE programme. Programme Grants Appl Res 2016;4(12).
11. Wylde V, Beswick A, Bruce J, Blom A, Howells N, Gooberman-Hill R. Chronic pain after total knee arthroplasty. EFORT Open Rev 2018;3(8):461-70.
12. Ackerman IN, Bennell KL. Does pre-operative physiotherapy improve outcomes from lower limb joint replacement surgery? A systematic review. Aust J Physiother 2004;50(1):25-30.

For peer review only

For peer review only - http://bmjopen.bmj.com/site/about/guidelines.xhtml

13. Bock M, Johansson T, Fritsch G, Flamm M, Hansbauer B, Mann E, Sonnichsen A. The impact of preoperative testing for blood glucose concentration and haemoglobin a1c on mortality, changes in management and complications in noncardiac elective surgery : A systematic review. Eur J Anaesthesiol 2015;32(3):152-9.
14. Cabilan CJ, Hines S, Munday J. The effectiveness of prehabilitation or preoperative exercise for surgical patients: A systematic review. JBI Database System Rev Implement Rep 2015;13(1):146-87.
15. Cheatham SW. Do patient factors and prehabilitation improve outcomes after total knee arthroplasty? Top Geriatr Rehabil 2013;29(1):17-24.
16. Coudeyre E, Jardin C, Givron P, Ribinik P, Revel M, Rannou F. Could preoperative rehabilitation modify postoperative outcomes after total hip and knee arthroplasty? Elaboration of French clinical practice guidelines. Annales de readaptation et de medecine physique : revue scientifique de la Societe francaise de reeducation fonctionnelle de readaptation et de medecine physique 2007;50(3):189-97.
17. Dauty M, Genty M, Ribinik P. Physical training in rehabilitation programs before and after total hip and knee arthroplasty. Annales de readaptation et de medecine physique : revue scientifique de la Societe francaise de reeducation fonctionnelle de readaptation et de medecine physique 2007;50(6):462-61.
18. Ellerby N, Hider S, Mallen C, Belcher J, Roddy E. Does intra-articular corticosteroid injection in the pre-operative period increase the risk of joint infection following hip or knee arthroplasty? A systematic review and meta-analysis. Rheumatology 2014;53:i132.
19. Gawel JA, Brown SE, Collins JC, McCallum C. Does pre-operative physical therapy improve post-surgical outcomes of patients undergoing a total knee and/or total hip arthroplasty? A systematic review. Physiother Pract Res 2013;34(1):9-20.
20. Gill SD, McBurney H. Does exercise reduce pain and improve physical function before hip or knee replacement surgery? A systematic review and meta-analysis of randomized controlled trials. Arch Phys Med Rehabil 2013;94(1):164-76.
21. Hoogeboom TJ, Oosting E, Vriezekolk JE, Veenhof V, Siemonsma PC, de Bie RA, van den Ende CHM, van Meeteren NLU. Therapeutic validity and effectiveness of preoperative exercise on functional recovery after joint replacement: a systematic review and meta-analysis. Nederlands Tijdschrift Voor Fysiotherapie 2012;122(4):150-60.
22. Johansson K, Nuutila L, Virtanen H, Katajisto J, Salantera S. Preoperative education for orthopaedic patients: systematic review. J Adv Nurs 2005;50(2):212-23.
23. Jordan RW, Smith NA, Chahal GS, Casson C, Reed MR, Sprowson AP. Enhanced education and physiotherapy before knee replacement; is it worth it? A systematic review. Physiotherapy 2014;100(4):305-12.

For peer review only - http://bmjopen.bmj.com/site/about/guidelines.xhtml

24. Louw A, Diener I, Butler DS, Puentedura EJ. Preoperative education addressing postoperative pain in total joint arthroplasty: review of content and educational delivery methods. Physiother Theory Pract 2013;29(3):175-94.
25. Lucas B. Does a pre-operative exercise programme improve mobility and function post-total knee replacement: a mini-review. J Orthop Nurs 2004;8(1):25-33.
26. Mak JCS, Fransen M, Jennings M, March L, Mittal R, Harris IA. Evidence-based review for patients undergoing elective hip and knee replacement. ANZ Journal of Surgery 2014;84(1-2):17-24.
27. McDonald S, Page MJ, Beringer K, Wasiak J, Sprowson A. Preoperative education for hip or knee replacement. Cochrane Database Syst Rev 2014;5:CD003526.
28. Santa Mina D, Clarke H, Ritvo P, Leung YW, Matthew AG, Katz J, Trachtenberg J, Alibhai SMH. Effect of total-body prehabilitation on postoperative outcomes: A systematic review and meta-analysis. Physiotherapy 2014;100(3):196-207.
29. Sau-Man Conny C, Wan-Yim I. The effectiveness of nurse-led preoperative assessment clinics for patients receiving elective orthopaedic surgery: A systematic review. J Perianesth Nurs 2016;31(6):465-74.
30. Shoemaker MJ, Gibson C, Saagman S. Preoperative exercise in individuals undergoing total knee arthroplasty. Top Geriatr Rehabil 2013;29(1):2-16.
31. Silkman Baker C, McKeon JM. Does preoperative rehabilitation improve patient-based outcomes in persons who have undergone total knee arthroplasty? A systematic review. PM & R 2012;4(10):756-67.
32. Simmons L, Smith T. Effectiveness of pre-operative physiotherapy-based programmes on outcomes following total knee arthroplasty: a systematic review and meta-analysis. Phys Ther Rev 2013;18(1):1-10.
33. Skoffer B, Dalgas U, Mechlenburg I. Progressive resistance training before and after total hip and knee arthroplasty: a systematic review. Clin Rehabil 2015;29(1):14-29.
34. Valkenet K, van de Port IGL, Dronkers JJ, de Vries WR, Lindeman E, Backx FJG. The effects of preoperative exercise therapy on postoperative outcome: a systematic review. Clin Rehabil 2011;25(2):99-111.
35. Wallis JA, Taylor NF. Pre-operative interventions (non-surgical and non-pharmacological) for patients with hip or knee osteoarthritis awaiting joint replacement surgery--a systematic review and meta-analysis. Osteoarthritis Cart 2011;19(12):1381-95.
36. Aydin D, Klit J, Jacobsen S, Troelsen A, Husted H. No major effects of preoperative education in patients undergoing hip or knee replacement--a systematic review. Dan Med J 2015;62(7):A5106.
37. Bay S, Kuster L, McLean N, Byrnes M, Kuster MS. A systematic review of psychological interventions in total hip and knee arthroplasty. BMC Musculoskelet Disord 2018;19(1):201.

For peer review only - http://bmjopen.bmj.com/site/about/guidelines.xhtml

38. Chen H, Li S, Ruan T, Liu L, Fang L. Is it necessary to perform prehabilitation exercise for patients undergoing total knee arthroplasty: meta-analysis of randomized controlled trials. Physician Sportsmed 2018;46(1):36-43.
39. Kwok IHY, Paton B, Haddad FS. Does pre-operative physiotherapy improve outcomes in primary total knee arthroplasty? - A systematic review. J Arthroplasty 2015;30(9):1657-63.
40. Ma J-X, Zhang L-K, Kuang M-J, Zhao J, Wang Y, Lu B, Sun L, Ma X-L. The effect of preoperative training on functional recovery in patients undergoing total knee arthroplasty: A systematic review and meta-analysis. Int J Surg 2018;51:205-12.
41. Peer MA, Rush R, Gallacher PD, Gleeson N. Pre-surgery exercise and post-operative physical function of people undergoing knee replacement surgery: A systematic review and meta-analysis of randomized controlled trials. J Rehabil Med 2017;49(4):304-15.
42. Umehara T, Tanaka R. Effective exercise intervention period for improving body function or activity in patients with knee osteoarthritis undergoing total knee arthroplasty: a systematic review and meta-analysis. Braz J Phys Ther 2018;22(4):265-75.
43. Zhang H, Hao X, Shuai T, Deng Y, Zeng Z, Song G. Efficacy of preoperative rehabilitation for total knee replacement: a systematic review. Chinese Journal of Evidence-Based Medicine 2017;17(9):1060-5.
44. Zhao Y, Jiang C, Peng H, Feng B, Li Y, Weng X. The effectiveness and safety of preoperative use of erythropoietin in patients scheduled for total hip or knee arthroplasty: A systematic review and meta-analysis of randomized controlled trials. Medicine 2016;95(27):e4122.
45. Chesham RA, Shanmugam S. Does preoperative physiotherapy improve postoperative, patient-based outcomes in older adults who have undergone total knee arthroplasty? A systematic review. Physiother Theory Pract 2017;33(1):9-30.
46. Wang L, Lee M, Zhang Z, Moodie J, Cheng D, Martin J. Does preoperative rehabilitation for patients planning to undergo joint replacement surgery improve outcomes? A systematic review and meta-analysis of randomised controlled trials. BMJ Open 2016;6(2):e009857.
47. Wylde V, Dennis J, Gooberman-Hill R, Beswick AD. Effectiveness of postdischarge interventions for reducing the severity of chronic pain after total knee replacement: systematic review of randomised controlled trials. BMJ Open 2018;8(2):e020368.
48. Beswick AD, Dennis J, Gooberman-Hill R, Blom AW, Wylde V. Are perioperative interventions effective in preventing chronic pain after primary total knee replacement? A systematic review. BMJ Open 2019;9(9):e028093.
49. Beswick A, Dennis J, Gooberman-Hill R, Wylde V, Bertram W. The effectiveness of pre-, peri- and post-operative interventions in preventing chronic pain in patients receiving primary total knee replacement: a systematic review. PROSPERO 2017.

For peer review only - http://bmjopen.bmj.com/site/about/guidelines.xhtml

50. Richardson WS, Wilson MC, Nishikawa J, Hayward RS. The well-built clinical question: a key to evidence-based decisions. ACP J Club 1995;123(3):A12-3.
51. Higgins JPT, Green S. Cochrane Handbook for Systematic Reviews of Interventions Version 5.1.0 [Updated March 2011]: The Cochrane Collaboration; 2011. Available from: http://www.cochrane-handbook.org.
52. Moher D, Liberati A, Tetzlaff J, Altman DG. Preferred reporting items for systematic reviews and meta-analyses: the PRISMA statement. J Clin Epidemiol 2009;62(10):1006-12.
53. Gooberman-Hill R, Burston A, Clark E, Johnson E, Nolan S, Wells V, Betts L, Pep R. Involving patients in research: considering good practice. Musculoskeletal Care 2013;11(4):187-90.
54. Murray DW, Fitzpatrick R, Rogers K, Pandit H, Beard DJ, Carr AJ, Dawson J. The use of the Oxford hip and knee scores. J Bone Joint Surg (Br) 2007;89(8):1010-4.
55. Bellamy N, Buchanan W, Goldsmith C, Campbell J, Stitt L. Validation study of WOMAC: a health status instrument for measuring clinically-important patient-relevant outcomes following total hip or knee arthroplasty in osteoarthritis. J Orthop Rheumatol 1988;15(12):1833-40.
56. Roos EM, Roos HP, Lohmander LS, Ekdahl C, Beynnon BD. Knee Injury and Osteoarthritis Outcome Score (KOOS)--development of a self-administered outcome measure. J Orthop Sports Phys Ther 1998;28(2):88-96.
57. Insall JN, Dorr LD, Scott RD, Scott WN. Rationale of the Knee Society clinical rating system. Clin Orthop Relat Res 1989(248):13-4.
58. Insall JN, Ranawat CS, Aglietti P, Shine J. A comparison of four models of total knee-replacement prostheses. J Bone Joint Surg (Am) 1976;58(6):754-65.
59. Wylde V, Bruce J, Beswick A, Elvers K, Gooberman-Hill R. The assessment of chronic post-surgical pain after knee replacement: A systematic review. Arthritis Care Res 2013;65(11):1795-803.
60. Hewlett SA. Patients and clinicians have different perspectives on outcomes in arthritis. J Rheumatol 2003;30(4):877-9.
61. Bachmeier CJM, March LM, Cross MJ, Lapsley HM, Tribe KL, Courtenay BG, Brooks PM. A comparison of outcomes in osteoarthritis patients undergoing total hip and knee replacement surgery. Osteoarthritis Cart 2001;9(2):137-46.
62. Ware JE, Jr., Sherbourne CD. The MOS 36-item short-form health survey (SF-36). I. Conceptual framework and item selection. Med Care 1992;30(6):473-83.
63. Ware J, Jr., Kosinski M, Keller SD. A 12-Item Short-Form Health Survey: construction of scales and preliminary tests of reliability and validity. Med Care 1996;34(3):220-33.
64. Dworkin RH, Turk DC, Farrar JT, Haythornthwaite JA, Jensen MP, Katz NP, Kerns RD, Stucki G, Allen RR, Bellamy N, Carr DB, Chandler J, Cowan P, Dionne R, Galer BS, Hertz

For peer review only

For peer review only - http://bmjopen.bmj.com/site/about/guidelines.xhtml

S, Jadad AR, Kramer LD, Manning DC, Martin S, McCormick CG, McDermott MP, McGrath P, Quessy S, Rappaport BA, Robbins W, Robinson JP, Rothman M, Royal MA, Simon L, Stauffer JW, Stein W, Tollett J, Wernicke J, Witter J. Core outcome measures for chronic pain clinical trials: IMMPACT recommendations. Pain 2005;113(1-2):9-19.

65. Moore RA, Eccleston C, Derry S, Wiffen P, Bell RF, Straube S, McQuay H. "Evidence" in chronic pain--establishing best practice in the reporting of systematic reviews. Pain 2010;150(3):386-9.

66. Higgins JPT, Altman DG, Gøtzsche PC, Jüni P, Moher D, Oxman AD, Savović J, Schulz KF, Weeks L, Sterne JAC. The Cochrane Collaboration's tool for assessing risk of bias in randomised trials. BMJ 2011;343:d5928.

67. Review Manager (RevMan) [Computer program]. Version 5.3. Copenhagen: The Nordic Cochrane Centre: The Cochrane Collaboration; 2014.

68. Guyatt G, Oxman AD, Akl EA, Kunz R, Vist G, Brozek J, Norris S, Falck-Ytter Y, Glasziou P, DeBeer H, Jaeschke R, Rind D, Meerpohl J, Dahm P, Schunemann HJ. GRADE guidelines: 1. Introduction-GRADE evidence profiles and summary of findings tables. J Clin Epidemiol 2011;64(4):383-94.

69. Villebro NM, Pedersen T, Moller AM, Tonnesen H. Long-term effects of a preoperative smoking cessation programme. Clin Respir J 2008;2(3):175-82.

70. Amory JK, Chansky HA, Chansky KL, Camuso MR, Hoey CT, Anawalt BD, Matsumoto AM, Bremner WJ. Preoperative supraphysiological testosterone in older men undergoing knee replacement surgery. J Am Geriatr Soc 2002;50(10):1698-701.

71. Moonen AFCM, Thomassen BJW, Knoors NT, van Os JJ, Verburg AD, Pilot P. Pre-operative injections of epoetin-alpha versus post-operative retransfusion of autologous shed blood in total hip and knee replacement: a prospective randomised clinical trial. J Bone Joint Surg (Br) 2008;90(8):1079-83.

72. Sousa RJG, Barreira PMB, Leite PTS, Santos ACM, Ramos MHSS, Oliveira AF. Preoperative *Staphylococcus aureus* screening/decolonization protocol before total joint arthroplasty-results of a small prospective randomized trial. J Arthroplasty 2016;31(1):234-9.

73. D'Lima DD, Colwell CW, Jr., Morris BA, Hardwick ME, Kozin F. The effect of preoperative exercise on total knee replacement outcomes. Clin Orthop Relat Res 1996(326):174-82.

74. Beaupre LA, Lier D, Davies DM, Johnston DBC. The effect of a preoperative exercise and education program on functional recovery, health related quality of life, and health service utilization following primary total knee arthroplasty. J Rheumatol 2004;31(6):1166-73.

75. Fernandes L, Roos E, Overgaard S, Villadsen A, Sogaard R. Supervised neuromuscular exercise prior to hip and knee replacement: 12-month clinical effect and cost-utility analysis alongside a randomised controlled trial. BMC Musculoskelet Disord 2017;18(1):5.

For peer review only - http://bmjopen.bmj.com/site/about/guidelines.xhtml

76. Huber EO, Roos EM, Meichtry A, de Bie RA, Bischoff-Ferrari HA. Effect of preoperative neuromuscular training (NEMEX-TJR) on functional outcome after total knee replacement: an assessor-blinded randomized controlled trial. BMC Musculoskelet Disord 2015;16:101.
77. Matassi F, Duerinckx J, Vandenneucker H, Bellemans J. Range of motion after total knee arthroplasty: the effect of a preoperative home exercise program. Knee Surg Sports Traumatol Arthrosc 2014;22(3):703-9.
78. Tungtrongjit Y, Weingkum P, Saunkool P. The effect of preoperative quadriceps exercise on functional outcome after total knee arthroplasty. J Med Assoc Thai 2012;95 Suppl 10:S58-66.
79. Rooks DS, Huang J, Bierbaum BE, Bolus SA, Rubano J, Connolly CE, Alpert S, Iversen MD, Katz JN. Effect of preoperative exercise on measures of functional status in men and women undergoing total hip and knee arthroplasty. Arthritis Rheum 2006;55(5):700-8.
80. Culliton SE, Bryant DM, MacDonald SJ, Hibbert KM, Chesworth BM. Effect of an e-learning tool on expectations and satisfaction following total knee arthroplasty: A randomized controlled trial. J Arthroplasty 2018;33(7):2153-8.
81. Ageberg E, Link A, Roos EM. Feasibility of neuromuscular training in patients with severe hip or knee OA: The individualized goal-based NEMEX-TJR training program. BMC Musculoskelet Disord 2010;11(1):126.
82. Jacobs CA, Christensen CP. Correlations between Knee Society function scores and functional force measures. Clin Orthop Relat Res 2009;467(9):2414-9.
83. Gademan MGJ, Hofstede SN, Vliet Vlieland TPM, Nelissen RGHH, Marang-van de Mheen PJ. Indication criteria for total hip or knee arthroplasty in osteoarthritis: a state-of-the-science overview. BMC Musculoskelet Disord 2016;17(1):463.
84. Hamilton DF, Lane JV, Gaston P, Patton JT, MacDonald D, Simpson AHRW, Howie CR. What determines patient satisfaction with surgery? A prospective cohort study of 4709 patients following total joint replacement. BMJ Open 2013;3(4):e002525.
85. Wylde V, Dennis J, Beswick AD, Bruce J, Eccleston C, Howells N, Peters TJ, Gooberman-Hill R. Systematic review of management of chronic pain after surgery. Br J Surg 2017;104(10):1293-306.
86. Goldsmith LJ, Suryaprakash N, Randall E, Shum J, MacDonald V, Sawatzky R, Hejazi S, Davis JC, McAllister P, Bryan S. The importance of informational, clinical and personal support in patient experience with total knee replacement: a qualitative investigation. BMC Musculoskelet Disord 2017;18(1):127.
87. Sorel JC, Veltman ES, Honig A, Poolman RW. The influence of preoperative psychological distress on pain and function after total knee arthroplasty: a systematic review and meta-analysis. Bone Joint J 2019;101-b(1):7-14.

For peer review only - http://bmjopen.bmj.com/site/about/guidelines.xhtml

88. Edwards RR, Haythornthwaite JA, Smith MT, Klick B, Katz JN. Catastrophizing and depressive symptoms as prospective predictors of outcomes following total knee replacement. Pain Res Manag 2009;14(4):307-11.

89. Riddle DL, Keefe FJ, Ang DC, Slover J, Jensen MP, Bair MJ, Kroenke K, Perera RA, Reed SD, McKee D, Dumenci L. Pain coping skills training for patients who catastrophize about pain prior to knee arthroplasty: A multisite randomized clinical trial. J Bone Joint Surg (Am) 2019;101(3):218-27.

For peer review only

For peer review only - http://bmjopen.bmj.com/site/about/guidelines.xhtml

PRISMA 2009 Flow Diagram

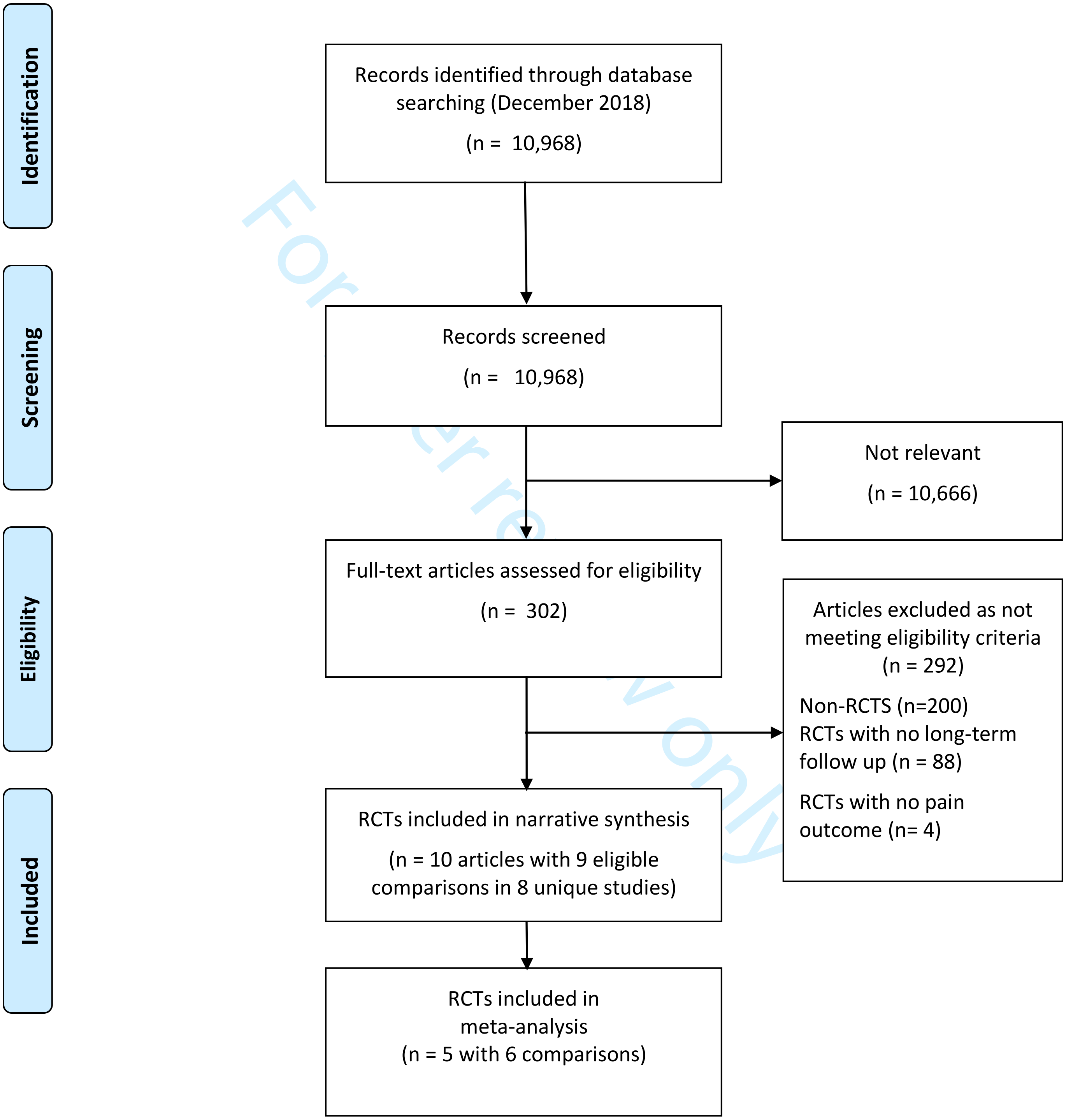

For peer review only

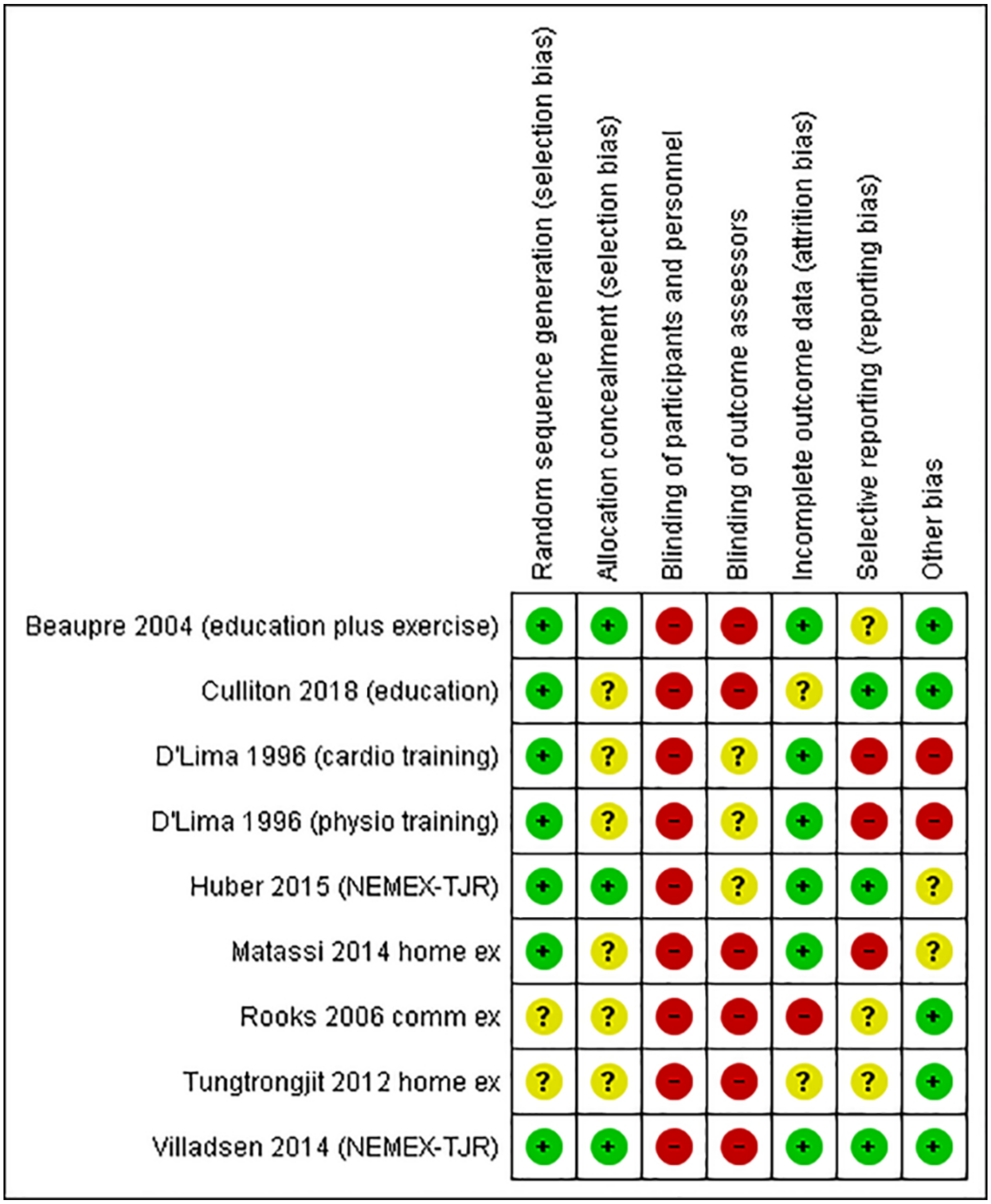
Random sequence generation (selection bias)
Allocation concealment (selection bias)
Blinding of participants and personnel
Blinding of outcome assessors
Incomplete outcome data (attrition bias)
Selective reporting (reporting bias)
Other bias
Beaupre 2004 (education plus exercise) + + − − + ? +
Culliton 2018 (education) + ? − − ? + +
D'Lima 1996 (cardio training) + ? − ? + − −
D'Lima 1996 (physio training) + ? − ? + − −
Huber 2015 (NEMEX-TJR) + + − ? + + ?
Matassi 2014 home ex + ? − − + − ?
Rooks 2006 comm ex ? ? − − − ? +
Tungtrongjit 2012 home ex ? ? − − ? ? +
Villadsen 2014 (NEMEX-TJR) + + − − + + +

	Exercise			Standard care				Std. Mean Difference
Study or Subgroup	Mean	SD	Total	Mean	SD	Total	Weight	IV, Random, 95% CI
**1.1.1 Pain at 6 - 12 months (various scales)**								
D'Lima 1996 (cardio training)	28.7	16.02	10	27	16.02	5	6.0%	0.10 [-0.97, 1.17]
D'Lima 1996 (physio training)	29	16.02	10	27	16.03	5	6.0%	0.12 [-0.96, 1.19]
Rooks 2006 comm ex	-2.4	2.7	14	-2.3	2	15	13.0%	-0.04 [-0.77, 0.69]
Fernandes 2017 NEMEX-TJR	74.2	18.6	37	73.2	21.7	37	33.3%	0.05 [-0.41, 0.50]
Huber 2015 NEMEX-TJR	87.76	11.09	17	85.95	13.39	19	16.1%	0.14 [-0.51, 0.80]
Tungtrongjit 2012 home ex	-2.5	3.1	30	-4.8	4.2	30	25.7%	0.62 [0.10, 1.13]
**Subtotal (95% CI)**			**118**			**111**	**100.0%**	**0.20 [-0.06, 0.47]**

Heterogeneity: $Tau^2 = 0.00$; $Chi^2 = 3.39$, df = 5 (P = 0.64); $I^2 = 0\%$

Test for overall effect: Z = 1.53 (P = 0.13)

Test for subgroup differences: Not applicable

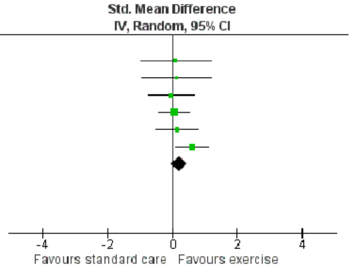

	Exercise			Standard care				Std. Mean Difference
**Study or Subgroup**	**Mean**	**SD**	**Total**	**Mean**	**SD**	**Total**	**Weight**	**IV, Random, 95% CI**
**2.1.1 Pain at 12 months (KOOS)**								
Fernandes 2017 NEMEX-TJR	74.2	18.6	37	73.2	21.7	37	67.4%	0.05 [-0.41, 0.50]
Huber 2015 NEMEX-TJR	87.76	11.09	17	85.95	13.39	19	32.6%	0.14 [-0.51, 0.80]
**Subtotal (95% CI)**			**54**			**56**	**100.0%**	**0.08 [-0.29, 0.45]**

Heterogeneity: $Tau^2 = 0.00$; $Chi^2 = 0.05$, $df = 1$ ($P = 0.82$); $I^2 = 0\%$

Test for overall effect: $Z = 0.42$ ($P = 0.68$)

Test for subgroup differences: Not applicable

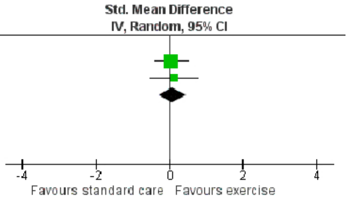

Study or Subgroup	Exercise & education Mean	SD	Total	Standard care Mean	SD	Total	Weight	Std. Mean Difference IV, Random, 95% CI
**3.1.2 Pain at 12 months (WOMAC)**								
Beaupre 2004 (education plus exercise)	82	13	51	80	16	58	100.0%	0.14 [-0.24, 0.51]
**Subtotal (95% CI)**			**51**			**58**	**100.0%**	**0.14 [-0.24, 0.51]**

Heterogeneity: Not applicable
Test for overall effect: Z = 0.70 (P = 0.48)

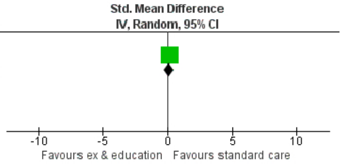

Test for subgroup differences: Not applicable

	Education			Standard care				Std. Mean Difference
**Study or Subgroup**	**Mean**	**SD**	**Total**	**Mean**	**SD**	**Total**	**Weight**	**IV, Random, 95% CI**
**4.1.3 Pain at 12 months (KOOS)**								
Culliton 2018 (education)	80.066	19.1	165	82.62	15.38	154	100.0%	-0.15 [-0.37, 0.07]
**Subtotal (95% CI)**			**165**			**154**	**100.0%**	**-0.15 [-0.37, 0.07]**
Heterogeneity: Not applicable								
Test for overall effect: Z = 1.30 (P = 0.19)								

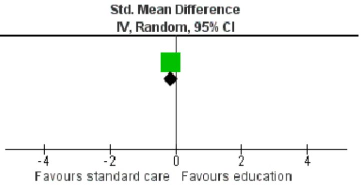

Test for subgroup differences: Not applicable

Appendix 1. Search strategy as applied in MEDLINE (tailored as relevant to other databases)

1 randomized controlled trial/ or randomized controlled trial.pt.

2 controlled clinical trial.pt.

3 randomized.ab.

4 placebo.ab.

5 randomly.ab

6 trial.ab

7 randomised.tw

8 1 or 2 or 3 or 4 or 5 or 6 or 7

9 review/

10 'systematic review$'.mp

11 9 or 10

12 8 or 11

13 Arthroplasty, Replacement, Knee/

14 Knee Prosthesis/

15 (arthoplast$ adj3 knee$).mp. [mp=title, abstract, original title, name of substance word, subject heading word, keyword heading word, protocol supplementary concept word, rare disease supplementary concept word, unique identifier]

16 (knee$ adj3 replac$).mp. [mp=title, abstract, original title, name of substance word, subject heading word, keyword heading word, protocol supplementary concept word, rare disease supplementary concept word, unique identifier]

17 (knee adj3 implant$).mp. [mp=title, abstract, original title, name of substance word, subject heading word, keyword heading word, protocol supplementary concept word, rare disease supplementary concept word, unique identifier]

18 13 or 14 or 15 or 16 or 17

19 12 and 18

For peer review only
